# Genome-wide meta-analysis uncovers novel loci influencing circulating leptin levels

**DOI:** 10.1038/ncomms10494

**Published:** 2016-02-01

**Authors:** Tuomas O. Kilpeläinen, Jayne F. Martin Carli, Alicja A. Skowronski, Qi Sun, Jennifer Kriebel, Mary F Feitosa, Åsa K. Hedman, Alexander W. Drong, James E. Hayes, Jinghua Zhao, Tune H. Pers, Ursula Schick, Niels Grarup, Zoltán Kutalik, Stella Trompet, Massimo Mangino, Kati Kristiansson, Marian Beekman, Leo-Pekka Lyytikäinen, Joel Eriksson, Peter Henneman, Jari Lahti, Toshiko Tanaka, Jian'an Luan, Fabiola Del Greco M, Dorota Pasko, Frida Renström, Sara M. Willems, Anubha Mahajan, Lynda M. Rose, Xiuqing Guo, Yongmei Liu, Marcus E. Kleber, Louis Pérusse, Tom Gaunt, Tarunveer S. Ahluwalia, Yun Ju Sung, Yolande F. Ramos, Najaf Amin, Antoinette Amuzu, Inês Barroso, Claire Bellis, John Blangero, Brendan M. Buckley, Stefan Böhringer, Yii-Der I Chen, Anton J. N. de Craen, David R. Crosslin, Caroline E. Dale, Zari Dastani, Felix R. Day, Joris Deelen, Graciela E. Delgado, Ayse Demirkan, Francis M. Finucane, Ian Ford, Melissa E. Garcia, Christian Gieger, Stefan Gustafsson, Göran Hallmans, Susan E. Hankinson, Aki S Havulinna, Christian Herder, Dena Hernandez, Andrew A. Hicks, David J. Hunter, Thomas Illig, Erik Ingelsson, Andreea Ioan-Facsinay, John-Olov Jansson, Nancy S. Jenny, Marit E. Jørgensen, Torben Jørgensen, Magnus Karlsson, Wolfgang Koenig, Peter Kraft, Joanneke Kwekkeboom, Tiina Laatikainen, Karl-Heinz Ladwig, Charles A. LeDuc, Gordon Lowe, Yingchang Lu, Pedro Marques-Vidal, Christa Meisinger, Cristina Menni, Andrew P. Morris, Richard H. Myers, Satu Männistö, Mike A. Nalls, Lavinia Paternoster, Annette Peters, Aruna D. Pradhan, Tuomo Rankinen, Laura J. Rasmussen-Torvik, Wolfgang Rathmann, Treva K. Rice, J Brent Richards, Paul M. Ridker, Naveed Sattar, David B. Savage, Stefan Söderberg, Nicholas J. Timpson, Liesbeth Vandenput, Diana van Heemst, Hae-Won Uh, Marie-Claude Vohl, Mark Walker, Heinz-Erich Wichmann, Elisabeth Widén, Andrew R. Wood, Jie Yao, Tanja Zeller, Yiying Zhang, Ingrid Meulenbelt, Margreet Kloppenburg, Arne Astrup, Thorkild I. A. Sørensen, Mark A. Sarzynski, D. C. Rao, Pekka Jousilahti, Erkki Vartiainen, Albert Hofman, Fernando Rivadeneira, André G. Uitterlinden, Eero Kajantie, Clive Osmond, Aarno Palotie, Johan G. Eriksson, Markku Heliövaara, Paul B. Knekt, Seppo Koskinen, Antti Jula, Markus Perola, Risto K. Huupponen, Jorma S. Viikari, Mika Kähönen, Terho Lehtimäki, Olli T. Raitakari, Dan Mellström, Mattias Lorentzon, Juan P. Casas, Stefanie Bandinelli, Winfried März, Aaron Isaacs, Ko W. van Dijk, Cornelia M. van Duijn, Tamara B. Harris, Claude Bouchard, Matthew A. Allison, Daniel I. Chasman, Claes Ohlsson, Lars Lind, Robert A. Scott, Claudia Langenberg, Nicholas J. Wareham, Luigi Ferrucci, Timothy M. Frayling, Peter P. Pramstaller, Ingrid B. Borecki, Dawn M. Waterworth, Sven Bergmann, Gérard Waeber, Peter Vollenweider, Henrik Vestergaard, Torben Hansen, Oluf Pedersen, Frank B. Hu, P Eline Slagboom, Harald Grallert, Tim D. Spector, J.W. Jukema, Robert J. Klein, Erik E Schadt, Paul W. Franks, Cecilia M. Lindgren, Rudolph L. Leibel, Ruth J. F. Loos

**Affiliations:** 1 The Novo Nordisk Foundation Center for Basic Metabolic Research, Section of Metabolic Genetics, Faculty of Health and Medical Sciences, University of Copenhagen, Universitetsparken 1, DIKU Building, Copenhagen 2100, Denmark; 2 MRC Epidemiology Unit, Institute of Metabolic Science, University of Cambridge, Cambridge CB2 0QQ, UK; 3 Genetics of Obesity and Related Metabolic Traits Program, Charles Bronfman Institute for Personalized Medicine, Icahn School of Medicine at Mount Sinai, New York, New York 10029, USA; 4 Department of Biochemistry and Molecular Biophysics, Columbia University, New York, New York 10032, USA; 5 Institute of Human Nutrition, Columbia University, New York, New York 10032, USA; 6 Channing Division of Network Medicine, Department of Medicine, Brigham and Women's Hospital and Harvard Medical School, Boston, Massachussetts 02115, USA; 7 Department of Nutrition, Harvard T.H. Chan School of Public Health, Boston, Massachussetts 02115, USA; 8 Research Unit of Molecular Epidemiology, Helmholtz Zentrum München - German Research Center for Environmental Health, Neuherberg 85764, Germany; 9 Institute of Epidemiology II, Helmholtz Zentrum München-German Research Center for Environmental Health, Neuherberg 85764, Germany; 10 German Center for Diabetes Research (DZD), München-Neuherberg 85764, Germany; 11 Department of Genetics, Washington University School of Medicine, St. Louis, Missouri 63110, USA; 12 Science for Life Laboratory, Uppsala University, Uppsala 750 85, Sweden; 13 Department of Medical Sciences, Molecular Epidemiology, Uppsala University, Uppsala 751 85, Sweden; 14 Wellcome Trust Centre for Human Genetics, University of Oxford, Oxford OX3 7BN, UK; 15 Cell and Developmental Biology Graduate Program, Weill Cornell Graduate School of Medical Sciences, Cornell University, New York, New York 10021, USA; 16 Icahn Institute for Genomics and Multiscale Biology, Icahn School of Medicine at Mount Sinai, New York, New York 10029, USA; 17 Divisions of Endocrinology and Genetics and Center for Basic and Translational Obesity Research, Boston Children's Hospital, Boston, Massachussetts 02115, USA; 18 Broad Institute of the Massachusetts Institute of Technology and Harvard University, Cambridge, Massachusetts 2142, USA; 19 Department of Genetics, Harvard Medical School, Boston, Massachusetts 02115, USA; 20 Department of Epidemiology Research, Statens Serum Institut, Copenhagen 2300, Denmark; 21 Institute of Social and Preventive Medicine, Lausanne University Hospital, Lausanne 1010, Switzerland; 22 Swiss Institute of Bioinformatics, Lausanne 1015, Switzerland; 23 Department of Cardiology, Leiden University Medical Center, Leiden 2333, The Netherlands; 24 Department of Gerontology and Geriatrics, Leiden University Medical Center, Leiden 2333, The Netherlands; 25 Department of Twin Research and Genetic Epidemiology, King's College London, London SE1 7EH, UK; 26 National Institute for Health Research Biomedical Research Centre at Guy's and St. Thomas' Foundation Trust, London SE1 9RT, UK; 27 Department of Health, National Institute for Health and Welfare, Helsinki FI-00271, Finland; 28 Institute for Molecular Medicine Finland, University of Helsinki, Helsinki FI-00290, Finland; 29 Department of Molecular Epidemiology, Leiden University Medical Center, Leiden 2300 RC, The Netherlands; 30 Department of Clinical Chemistry, Fimlab Laboratories, Tampere FI-33101, Finland; 31 Department of Clinical Chemistry, University of Tampere School of Medicine, Tampere FI-33014, Finland; 32 Centre for Bone and Arthritis Research, Department of Internal Medicine and Clinical Nutrition, Institute of Medicine, Sahlgrenska Academy, University of Gothenburg, Gothenburg 413 45, Sweden; 33 Department of Human Genetics, Leiden University Medical Center, Leiden 2333, The Netherlands; 34 Department of Clinical Genetics, Amsterdam Medical Center, Amsterdam 1081 HV, The Netherlands; 35 Institute of Behavioural Sciences, University of Helsinki, Helsinki FI-00014, Finland; 36 Folkhälsan Research Center, Helsinki FI-00290, Finland; 37 Translational Gerontology Branch, National Institute on Aging, Baltimore, Maryland 21225, USA; 38 Center for Biomedicine, European Academy Bozen/Bolzano (EURAC) - Affiliated Institute of the University of Lübeck, Bolzano 39100, Italy; 39 Genetics of Complex Traits, University of Exeter Medical School, University of Exeter, Exeter EX2 5DW, UK; 40 Department of Clinical Sciences, Genetic and Molecular Epidemiology Unit, Lund University, Malmö 20502, Sweden; 41 Department of Biobank Research, Umeå University, Umeå 90187, Sweden; 42 Department of Epidemiology, Erasmus MC, Rotterdam 3015 GE, The Netherlands; 43 Division of Preventive Medicine, Brigham and Women's Hospital, Boston, Massachussetts 02215, USA; 44 Department of Pediatrics, LABioMed at Harbor-UCLA Medical Center, Institute for Translational Genomics and Population Sciences, Torrance, California 90502, USA; 45 Center for Human Genetics, Division of Public Health Sciences, Wake Forest School of Medicine, Winston-Salem, North Carolina 27157, USA; 46 Medical Faculty Mannheim, Vth Department of Medicine, Heidelberg University, Mannheim 68167, Germany; 47 Department of Kinesiology, Laval University, Quebec City, Quebec, Canada G1V 0A6; 48 Institute of Nutrition and Functional Foods, Quebec City, Quebec, Canada G1V 0A6; 49 MRC Integrative Epidemiology Unit and School of Social and Community Medicine, University of Bristol, Bristol BS82BN, UK; 50 COPSAC, Copenhagen Prospective Studies on Asthma in Childhood, Herlev and Gentofte Hospital, University of Copenhagen, Ledreborg Allé, Copenhagen DK-2820, Denmark; 51 Steno Diabetes Center, Gentofte DK-2820, Denmark; 52 Division of Biostatistics, Washington University School of Medicine, St. Louis, Missouri 63108, USA; 53 Department of Psychiatry, Washington University School of Medicine, St. Louis, Missouri 63110, USA; 54 Genetic Epidemiology Unit, Department of Epidemiology, Erasmus MC, Rotterdam 3015 GE, The Netherlands; 55 Faculty of Epidemiology and Population Health, London School of Hygiene and Tropical Medicine, London WC1E 7HT, UK; 56 Wellcome Trust Sanger Institute, Hinxton CB10 1SA, UK; 57 NIHR Cambridge Biomedical Research Centre, Institute of Metabolic Science, Addenbrooke's Hospital, Cambridge CB2 0QQ, UK; 58 The University of Cambridge Metabolic Research Laboratories, Wellcome Trust-MRC Institute of Metabolic Science, Cambridge CB2 0QQ, UK; 59 Human Genetics, Genome Institute of Singapore, Agency for Science, Technology and Research of Singapore, Singapore 138672, Singapore; 60 Genomics Research Centre, Institute of Health and Biomedical Innovation, Queensland University of Technology, Brisbane, Queensland 4001, Australia; 61 Texas Biomedical Research Institute, San Antonio, Texas 78245, USA; 62 Department of Pharmacology and Therapeutics, University College Cork, Cork T12 YT57, Ireland; 63 Division of Medical Genetics, Department of Medicine, University of Washington, Seattle, Washington 98195, USA; 64 Department of Genome Sciences, University of Washington, Seattle, Washington 98195, USA; 65 Department of Human Genetics, McGill University, Montreal, Quebec, Canada H3A 0G4; 66 Robertson Center for Biostatistics, University of Glasgow, Glasgow G12 8QQ, UK; 67 National Heart, Lung, and Blood Institute, NIH, Bethesda, Maryland 2089, USA; 68 Institute of Genetic Epidemiology, Helmholtz Zentrum München, German Research Center for Environmental Health, Neuherberg 85764, Germany; 69 Department of Biostatistics and Epidemiology, School of Public Health and Health Sciences, University of Massachusetts, Amherst, Massachusetts 01003, USA; 70 Department of Epidemiology, Harvard T.H. Chan School of Public Health, Boston, Massachusetts 02115, USA; 71 Institute for Clinical Diabetology, German Diabetes Center, Leibniz Center for Diabetes Research at Heinrich Heine University Düsseldorf, Düsseldorf 40225, Germany; 72 Laboratory of Neurogenetics, National Institute on Aging, Bethesda, Maryland 20892, USA; 73 Department of Nutrition and Epidemiology, Harvard T.H. Chan School of Public Health, Boston, Massachusetts 02115, USA; 74 Hannover Unified Biobank, Hannover Medical School, Hannover 30625, Germany; 75 Institute for Human Genetics, Hannover Medical School, Hannover 30625, Germany; 76 Division of Cardiovascular Medicine, Department of Medicine, Stanford University School of Medicine, Stanford, California 94305, USA; 77 Department of Rheumatology, Leiden University Medical Center, Leiden 2333, The Netherlands; 78 Department of Physiology, Institute of Neuroscience and Physiology, Sahlgrenska Academy, University of Gothenburg, Gothenburg 41345, Sweden; 79 Laboratory for Clinical Biochemistry Research, Department of Pathology and Laboratory Medicine, University of Vermont College of Medicine, Colchester, Vermont 05405, USA; 80 Research Centre for Prevention and Health, Glostrup University Hospital, Glostrup 2600, Denmark; 81 Faculty of Medicine, University of Aalborg, Aalborg 9100, Denmark; 82 Faculty of Health and Medical Sciences, University of Copenhagen, Copenhagen 2200, Denmark; 83 Clinical and Molecular Osteoporosis Research Unit, Department of Clinical Sciences and Orthopaedic Surgery, Lund University, Skåne University Hospital, Malmö 21428, Sweden; 84 Department of Internal Medicine II - Cardiology, University of Ulm, Ulm 89081, Germany; 85 Deutsches Herzzentrum München, Technische Universität München, Munich 80636, Germany; 86 DZHK (German Centre for Cardiovascular Research), partner site Munich Heart Alliance, Munich 80539, Germany; 87 Department of Epidemiology and Biostatistics, Harvard T.H. Chan School of Public Health, Boston, Massachussetts 02115, USA; 88 Institute of Public Health and Clinical Nutrition, University of Eastern Finland, Kuopio FI-70211, Finland; 89 Hospital District of North Karelia, Joensuu FI-80210, Finland; 90 Department of Psychosomatic Medicine and Psychotherapy, Klinikum Rechts der Isar, Technische Universität München, Munich 81675, Germany; 91 Division of Molecular Genetics, Department of Pediatrics, Columbia University, New York, New York 10029, USA; 92 Institute of Cardiovascular and Medical Sciences, University of Glasgow, Glasgow G12 8QQ, UK; 93 Department of Internal Medicine, Lausanne University Hospital, Lausanne 1011, Switzerland; 94 Department of Biostatistics, University of Liverpool, Liverpool L69 3GA, UK; 95 Department of Neurology, Boston University School of Medicine, Boston, Massachussetts 02118, USA; 96 Harvard Medical School, Boston, Massachussetts 02115, USA; 97 Human Genomics Laboratory, Pennington Biomedical Research Center, Baton Rouge, Los Angeles 70808, USA; 98 Preventive Medicine, Northwestern Feinberg School of Medicine, Chicago, Illinois 60611, USA; 99 Institute for Biometrics and Epidemiology, German Diabetes Center, Leibniz Center for Diabetes Research at Heinrich Heine University Düsseldorf, Düsseldorf 40225, Germany; 100 Department of Medicine, Human Genetics and Epidemiology, McGill University, Montreal, Quebec, Canada H3A 0G4; 101 Faculty of Medicine, BHF Glasgow Cardiovascular Research Centre, Glasgow G12 8QQ, UK; 102 Department of Public Health and Clinical Medicine, Cardiology and Heart Centre, Umeå University, Umeå 90187, Sweden; 103 School of Nutrition, Laval University, Quebec City, Quebec, Canada G1V 0A6; 104 Institute of Cellular Medicine, Newcastle University, Newcastle upon Tyne NE1 7RU, UK; 105 Institute of Medical Informatics, Biometry and Epidemiology, Ludwig-Maximilians-Universität and Klinikum Grosshadern, Munich 80336, Germany; 106 Institute of Epidemiology I, Helmholtz Zentrum München-German Research Center for Environmental Health, Neuherberg 85764, Germany; 107 Institute of Medical Statistics and Epidemiology, Technical University Munich, Munich 81675, Germany; 108 German Center for Cardiovascular Research (DZHK e.V.), partner site Hamburg/Kiel/Lübeck, Hamburg 20246, Germany; 109 Clinic for General and Interventional Cardiology, University Heart Center Hamburg, Hamburg 20246, Germany; 110 Department of Clinical Epidemiology, Leiden University Medical Center, Leiden 2333, The Netherlands; 111 Faculty of Science, Department of Nutrition, Exercise, and Sports, University of Copenhagen, Copenhagen 1165, Denmark; 112 Institute of Preventive Medicine, Bispebjerg and Frederiksberg Hospitals, The Capital Region, Copenhagen 2000, Denmark; 113 Department of Internal Medicine, Erasmus MC, Rotterdam 3015 GE, The Netherlands; 114 Children's Hospital, Helsinki University Central Hospital and University of Helsinki, Helsinki FI-00014, Finland; 115 Department of Obstetrics and Gynaecology, MRC Oulu, Oulu University Central Hospital and University of Oulu, Oulu 90220, Finland; 116 MRC Lifecourse Epidemiology Unit, University of Southampton, Southampton General Hospital, Southampton SO16 6YD, UK; 117 Center for Human Genetic Research, Psychiatric and Neurodevelopmental Genetics Unit, Massachusetts General Hospital, Boston, Massachusetts 02114, USA; 118 Department of General Practice and Primary Health Care, University of Helsinki, Helsinki FI-00014, Finland; 119 University of Tartu, Estonian Genome Centre, Tartu 51010, Estonia; 120 Department of Pharmacology, Drug Development and Therapeutics, University of Turku, Turku FI-20520, Finland; 121 Unit of Clinical Pharmacology, Turku University Hospital, Turku FI-20520, Finland; 122 Division of Medicine, Turku University Hospital, Turku FI-20520, Finland; 123 Department of Medicine, University of Turku, Turku FI-20520, Finland; 124 Department of Clinical Physiology, Tampere University Hospital, Tampere FI-33521, Finland; 125 Department of Clinical Physiology, University of Tampere School of Medicine, Tampere FI-33014, Finland; 126 Department of Clinical Physiology and Nuclear Medicine, Turku University Hospital, Turku FI-2051, Finland; 127 Research Centre of Applied and Preventive Cardiovascular Medicine, University of Turku, Turku FI-20520, Finland; 128 Farr Institute of Health Informatics, University College London, London NW1 2DA, UK; 129 Geriatric Unit, Azienda Sanitaria Firenze, Florence 50122, Italy; 130 Synlab Academy, Synlab Services LLC, Mannheim 68161, Germany; 131 Clinical Institute of Medical and Chemical Laboratory Diagnostics, Medical University of Graz, Graz 8010, Austria; 132 Center of Medical Systems Biology, Leiden 2300 RC, The Netherlands; 133 Laboratory of Epidemiology and Population Science, National Institute on Aging, Bethesda, Maryland 20892, USA; 134 Family and Preventive Medicine, University of California–San Diego, La Jolla, California 92161, USA; 135 Department of Medical Sciences, Cardiovascular Epidemiology, Uppsala University, Uppsala 75185, Sweden; 136 Department of Neurology, General Central Hospital, Bolzano 39100, Italy; 137 Department of Neurology, University of Lübeck, Lübeck 23562, Germany; 138 GlaxoSmithKline, King of Prussia, Pennsylvania 19406, USA; 139 Department of Medical Genetics, University of Lausanne, Lausanne 1015, Switzerland; 140 Faculty of Health Sciences, University of Southern Denmark, Odense 5230, Denmark; 141 Interuniversity Cardiology Institute of the Netherlands, Utrecht 3511 EP, The Netherlands; 142 Durrer Center for Cardiogenetic Research, Amsterdam 1105 AZ, The Netherlands; 143 Department of Public Health and Clinical Medicine, Umeå University, Umeå 90187, Sweden; 144 Program in Medical and Population Genetics, Broad Institute, Cambridge, Massachussetts 02142, USA; 145 The Big Data Institute, University of Oxford, Oxford OX1 2JD, UK; 146 The Mindich Child Health and Development Institute, Icahn School of Medicine at Mount Sinai, New York, New York 10029, USA

## Abstract

Leptin is an adipocyte-secreted hormone, the circulating levels of which correlate closely with overall adiposity. Although rare mutations in the leptin (*LEP*) gene are well known to cause leptin deficiency and severe obesity, no common loci regulating circulating leptin levels have been uncovered. Therefore, we performed a genome-wide association study (GWAS) of circulating leptin levels from 32,161 individuals and followed up loci reaching *P*<10^−6^ in 19,979 additional individuals. We identify five loci robustly associated (*P*<5 × 10^−8^) with leptin levels in/near *LEP*, *SLC32A1*, *GCKR*, *CCNL1* and *FTO*. Although the association of the *FTO* obesity locus with leptin levels is abolished by adjustment for BMI, associations of the four other loci are independent of adiposity. The *GCKR* locus was found associated with multiple metabolic traits in previous GWAS and the *CCNL1* locus with birth weight. Knockdown experiments in mouse adipose tissue explants show convincing evidence for *adipogenin*, a regulator of adipocyte differentiation, as the novel causal gene in the *SLC32A1* locus influencing leptin levels. Our findings provide novel insights into the regulation of leptin production by adipose tissue and open new avenues for examining the influence of variation in leptin levels on adiposity and metabolic health.

## Abstract (web summary)

This meta-analysis of genome-wide association studies identifies four genetic loci associated with circulating leptin levels independent of adiposity. Examination in mouse adipose tissue explants provides functional support for the leptin-associated loci.

## Graphical abstract

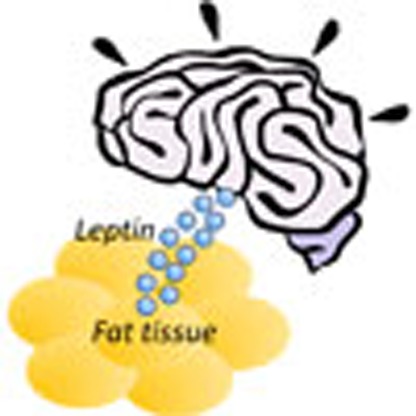

Leptin is an adipocyte-secreted hormone that influences long-term regulation of energy homeostasis by informing the brain about the amount of stored body fat^1^,^2^. Circulating leptin levels correlate closely with measures of adiposity, such as body fat mass and body mass index (BMI)^3^. Yet, at any given level of adiposity, there is substantial variation in circulating leptin levels^4^, of which estimated 30–50% is explained by genetic factors^5^,^6^,^7^.

Rare homozygous loss-of-function mutations in the leptin-encoding gene (*LEP*) cause leptin deficiency that leads to hyperphagia and severe obesity, which can be corrected by exogenous leptin administration^8^. Leptin-deficient children are born with a normal birth weight but exhibit rapid weight gain in the first few months of life. They show marked abnormalities of T-cell number and function, and have high rates of childhood infection^9^. Hypothalamic hypothyroidism is present, characterized by a low free thyroxine and high serum thyroid-stimulating hormone levels^10^. Pubertal development generally does not occur due to hypogonadotropic hypogonadism^10^. Individuals heterozygous for leptin mutations exhibit a partial leptin deficiency with higher body fat than control individuals^11^.

Candidate gene studies, typically small in size, have reported associations of two common variants (A19G (rs2167270, minor allele frequency (MAF) 35%) and G2548A (rs7799039, MAF 49%)) in the promoter or 5′-untranslated region of *LEP* with circulating leptin levels in the general population, but these results are inconclusive^12^,^13^,^14^,^15^,^16^. The same *LEP* variants have been studied for association with obesity, but a meta-analysis of the published results (*n*_A19G_=918 and *n*_G2548A_=2,174) found no evidence of such association^17^. Candidate gene studies of *LEP* were published before the human genome sequence was extensively characterized and are therefore restricted to the variants known at that time. Furthermore, although *LEP* is an obvious candidate, variants in other genes may also influence circulating leptin levels by regulating leptin production, secretion, clearance or response. Identification of such leptin-regulating genes could provide novel insights into mechanisms that regulate energy homeostasis and neuro-endocrine function^1^,^2^.

In this study, we sought to identify genetic loci associated with circulating leptin levels by a genome-wide meta-analysis. Given the strong correlation between leptin and adiposity, we also examined genome-wide associations with circulating leptin levels adjusted for BMI, to identify loci associated with leptin levels independent of BMI.

## Results

### Stage 1 genome-wide meta-analysis in 32,161 individuals

We first performed a meta-analysis of the results from genome-wide associations between ∼2.5 million genotyped and HapMap-imputed single-nucleotide polymorphisms (SNPs) and circulating leptin levels, including up to 32,161 individuals of European descent from 23 studies (Supplementary Table 1). After logarithmic transformation that normalized the distribution of leptin levels and adjusting for age and sex, we carried out association analyses within each study and subsequently meta-analysed the study-specific results. To identify loci associated with circulating leptin levels independently of adiposity, we performed a meta-analysis of genome-wide associations in which we additionally adjusted for BMI. We also performed secondary genome-wide meta-analyses in men (*n*=13,363) and women (*n*=18,698) separately, as women generally have higher leptin levels than men, primarily due to larger percentage of body fat and greater subcutaneous fat storage^18^.

Two loci, near the *LEP* and *SLC32A1* genes, reached genome-wide significance (*P*<5 × 10^−8^) in the BMI-adjusted meta-analysis of men and women combined (Table 1). To confirm these associations and to identify additional leptin-associated loci, we took forward all independent (pairwise distance >500 kb and *r*^2^<0.1) SNPs reaching *P*<10^−6^ with leptin levels with or without adjustment for BMI in meta-analyses of all individuals combined, men only or women only, for follow-up in stage 2 (Supplementary Tables 2–4).

### Stage 2 follow-up in 19,979 individuals identifies five loci

We examined the associations of the loci taken forward from stage 1 in up to 19,979 additional individuals of European descent from 13 studies (Supplementary Table 5). All studies performed the same association analyses as described in Stage 1; that is, with and without adjustment for BMI and in men and women combined, as well as separately. Finally, after performing a joint meta-analysis of the stage 1 and stage 2 results, five independent SNPs reached genome-wide significance (*P*<5 × 10^−8^) in the combined meta-analyses of men and women (Table 1). In the BMI-adjusted meta-analysis, we confirmed genome-wide significant associations for the loci near *LEP* and *SLC32A1*, and identified an additional locus in *GCKR*. In the BMI-unadjusted meta-analysis, we identified two additional loci near *CCNL1* and in *FTO*. A locus in *COBLL1*, previously identified for association with BMI-adjusted waist–hip ratio (WHR_adjBMI_)^19^, blood triglycerides^20^ and risk of type 2 diabetes^21^, reached *P*=1 × 10^−6^ with BMI-unadjusted leptin and *P*=2 × 10^−6^ with BMI-adjusted leptin levels, with the leptin-increasing allele being associated with lower WHR_adjBMI_, triglycerides and risk of type 2 diabetes.

**Figure 1:**
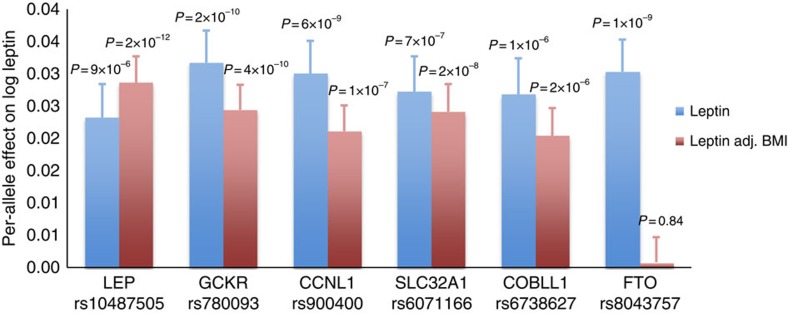
Meta-analysis results for the leptin-associated loci. The bars show the additive effect of the loci in or near *LEP, GCKR, CCNL1*, *SLC32A1, COBLL1* and *FTO* on BMI-unadjusted and BMI-adjusted leptin levels in the meta-analysis of Stage 1 and Stage 2 combined. The error bars indicate s.e.

The estimated effects of five of the six loci (in/near *LEP*, *SLC32A1*, *GCKR*, *CCNL1* or *COBLL1*) on leptin levels did not markedly differ in magnitude between the BMI-unadjusted and BMI-adjusted models, suggesting that these associations are not mediated by adiposity *per se* (Fig. 1). In contrast, the association between the *FTO* locus and leptin levels was completely abolished after adjusting for BMI, indicating that the association with leptin is entirely mediated by the well-established association between *FTO* and BMI^22^ (Fig. 1).

BMI is the most commonly used index of adiposity, but it is not a direct measure of adiposity and it does not distinguish between lean and fat body mass. To assess whether adjustment for a more direct measure of adiposity could enhance our ability to identify adiposity-independent loci, we performed secondary analyses in 13 studies that had data on both BMI and body fat percentage assessed by dual-energy X-ray absorptiometry or bioimpedance analysis (*n*=18,980 or 59% of stage 1 sample). The analysis showed no marked differences in the effect sizes between the BMI and body fat percentage-adjusted results for the leptin-associated *LEP*, *SLC32A1*, *CCNL1*, *GCKR*, *COBLL1* and *FTO* loci (Supplementary Table 6), suggesting that adjustment for BMI as compared with a more direct measure of adiposity did not compromise our ability to identify adiposity-independent leptin-associated loci.

### Effects on other traits and potential functional roles

We took forward the genome-wide significant leptin loci near *LEP*, near *SLC32A1*, in *GCKR* and near *CCNL1*, to examine their associations with obesity-related and metabolic traits and to more directly assess their putative roles in the control of circulating leptin. We also took forward the locus near *COBLL1*, given its robust association with WHR_adjBMI_^19^, even though it just missed the genome-wide significance threshold for association with BMI-adjusted and BMI-unadjusted leptin levels (Table 1). As the *FTO*-leptin association was completely accounted for by *FTO*'s association with BMI (Fig. 1), extensively described in the literature^23^, we did not include this locus in our follow-up analyses.

To examine the associations of the identified loci with obesity-related and metabolic traits, we performed look-ups in the data from relevant genetic consortia (Supplementary Table 7). To study the associations of the leptin-associated loci with the expression of nearby genes, we performed *cis*-expression quantitative trait locus (eQTL) analyses in several human tissues, including the subcutaneous (*n*=776) and omental fat (*n*=742), liver (*n*=567), lymphocytes (*n*=778), brain (*n*=193) and skin (*n*=667) (Supplementary Table 8). We also examined the regulatory functions of these loci by studying their enrichment with functional genomic elements in data from the Roadmap Epigenomics Project^24^. Finally, to identify the causal genes in the leptin-associated loci, we performed *ex vivo* knockdown studies of adipocyte-expressed genes using small interfering RNA (siRNA) in explanted mouse adipose tissue.

### Common variation near *LEP* regulates leptin levels

**Figure 2:**
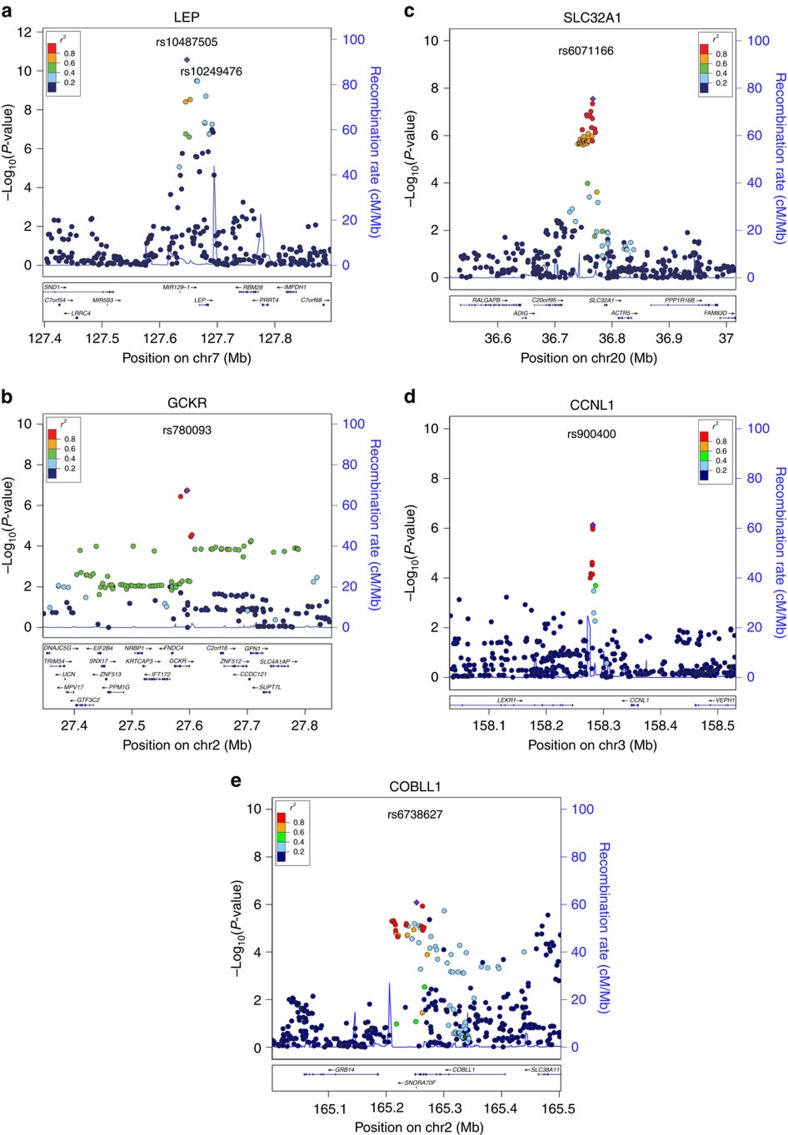
Regional plots for the leptin-associated loci. Regional plots for the loci in or near *LEP* (**a**), *GCKR* (**b**), *SLC32A1* (**c**) and *CCNL1* (**d**), which reached genome-wide significance in the combined meta-analysis of Stage 1 and Stage 2 for BMI-unadjusted or BMI-adjusted leptin levels. The *COBLL1* locus (**e**) that reached *P*=1 × 10^−6^ with BMI-unadjusted and *P*=2 × 10^−6^ with BMI-adjusted leptin levels is also shown. For the locus near *LEP* (A), the rs10249476 SNP, located in a previously identified adipocyte-specific enhancer region^26^, is indicated.

The rs10487505 variant (MAF 49%) is located 21 kb from *LEP* (Fig. 2a) and is in modest linkage disequilibrium (LD) (*r*^2^=0.4, *D*′=0.8) with the A19G (rs2167270, MAF 35%) variant that has been extensively studied in candidate gene studies but whose associations with increased levels of leptin and obesity have been inconclusive^13^,^16^. The leptin-increasing allele of the rs10487505 variant has been nominally associated with weight regain after bariatric surgery in a candidate gene-based analysis of 1,443 patients^25^. Look-ups in consortium data showed a nominally significant association for the leptin-decreasing allele of rs10487505 with higher BMI in the GIANT Consortium (*P*=0.03, *N*=221,677), as well as with increased risk of early-onset obesity (*P*=0.04, *N*=13,848) and higher birth weight (*P*=0.02, *N*=26,836) in the EGG Consortium (Supplementary Table 7). Although *LEP* is an obvious candidate gene to account for the association with circulating leptin levels, the rs10487505 variant was not associated with *LEP* messenger RNA expression in the omental or subcutaneous adipose tissue (SCAT), liver, lymphocytes, brain or skin (Supplementary Tables 8 and 9).

A variant in strong LD with rs10487505 (rs6979832, *r*^2^=0.98) overlapped with predicted enhancer elements in all three adipose cell lines of the Roadmap Epigenomics Project^24^. Further, a previous study identified a 465-bp adipocyte-specific enhancer region 4.5 kb upstream from the *LEP* transcription start site by using luciferase assays and chromatin state mapping^26^. This region harbours rs10249476 that is in modest LD with rs10487505 (*r*^2^=0.4, *D*′=0.8) and reached the second most significant association with BMI-adjusted leptin levels in stage 1 meta-analysis (*P*=3 × 10^−10^; *n*=30,810) (Fig. 2a).

Collectively, although the locus near *LEP* overlaps with predicted enhancer elements, the lack of association with *LEP* transcript expression in the fasting state suggests that other mechanisms may be involved in mediating the association of this locus with leptin levels, such as an effect on *LEP* expression in the fed state^27^ or an effect on leptin protein secretion.

To validate our knockdown strategy for subsequent analyses of candidate genes in loci other than the locus near *LEP*, we used siRNA against *Lep* in mouse adipose tissue explants. Electroporation of the perigonadal adipose tissue (PGAT) explants with siRNA against *Lep* resulted in a 92% decrease in *Lep* mRNA (*P*<1 × 10^−4^) and a 92% decrease in secreted leptin (*P*=4 × 10^−4^) (Fig. 4a,b, Supplementary Fig. 1C,D and Supplementary Table 10). In addition, to determine whether electroporation with siRNA altered other secretory function(s) of the perigonadal explants, we measured secretion of adiponectin and found no changes associated with *Lep* knockdown (Fig. 4d and Supplementary Fig. 1E).

### *ADIG* may regulate leptin expression

The intergenic rs6071166 variant, ∼20 kb from the *SLC32A1* gene (Fig. 2c), reached genome-wide significance for association with BMI-adjusted leptin levels and has not been previously identified for association with any other traits. In look-ups of genome-wide association study (GWAS) consortium data, we did not find significant association with other obesity-related or metabolic traits (Supplementary Table 7). The rs6071166 variant was not associated with the mRNA expression of nearby genes in the adipose tissue, liver, lymphocytes, brain or skin (Supplementary Tables 8 and 9).

**Figure 3:**
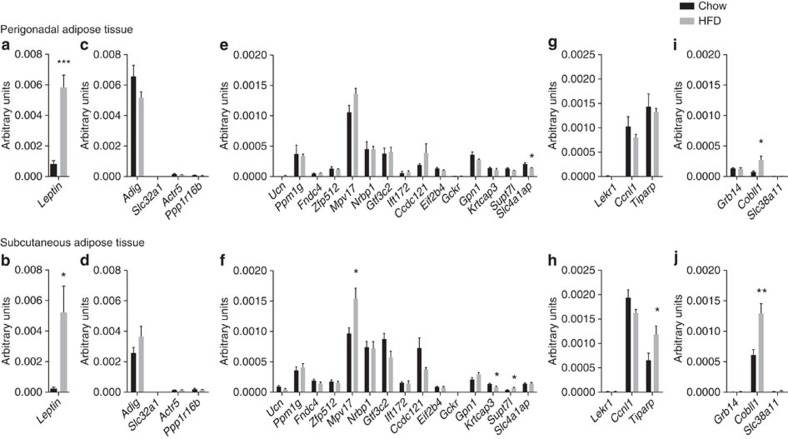
Expression of murine homologues of genes. Expression of murine homologues of genes located within *Lep* (**a**,**b**), *Slc32a1* (**c**,**d**), *Gckr* (**e**,**f**), *Ccnl1* (**g**,**h**) and *Cobll1* loci (**i**,**j**) in PGAT and SCAT from 4-month-old mice fed chow (black bars) or high-fat diet (HFD; grey bars). Quantitative PCR (qPCR) transcripts were normalized using *ActB*, *Rplp0*, *Gapdh* and *Ppia* as housekeeping genes. *N*=5 mice per group. T-test. *P<0.05, **P<0.01 and ***P<0.001.

**Figure 4:**
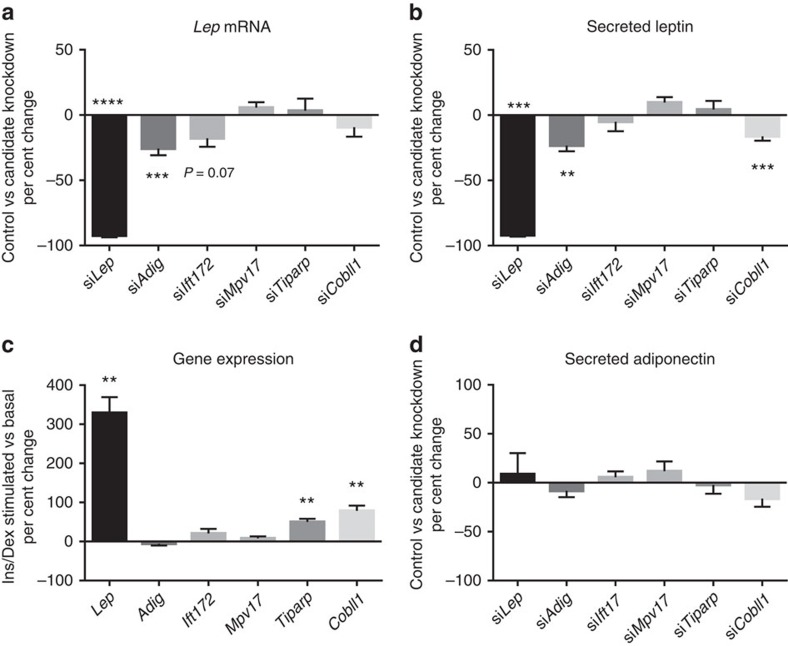
Candidate gene knockdown studies in PGAT explants. Changes in *Lep* mRNA expression (**a**) and secretion into media (**b**) following candidate gene knockdown in PGAT explants following stimulation with insulin and dexamethasone for 12 h. Gene expression induced by stimulation with insulin and dexamethasone (**c**) *N*=5–13 mice per group (3 replicates/condition/mouse). Secreted adiponectin was measured as a control for non-leptin secretory function (**d**) *N*=5 mice per group. Two-way repeated measures analysis of variance (ANOVA). *P<0.05, **P<0.01, ***P<0.001 and ****P<0.0001.

To identify the potential causal gene in this locus using the mouse PGAT explant model described above, we first measured the expression levels of murine homologues of genes surrounding the lead variant associated with circulating leptin levels. We tested PGAT and SCAT of 4-month-old C57BL/6J mice fed chow or high-fat diet (Fig. 3c,d). In addition, we analysed candidate gene expression in other tissues (liver and hypothalamus) that we predicted could play a role in circulating leptin levels via effects on leptin clearance or response (Supplementary Fig. 7). Genes were considered strong candidates if they were highly expressed in adipose tissue and/or if they were regulated by high-fat diet feeding in a manner similar to *Lep*. This analysis identified *adipogenin* (*Adig*) as a candidate gene in the *SLC32A1* locus; *Adig* is highly expressed in the adipose tissue, in contrast to other nearby genes. To test whether *Adig* affected *Lep* expression, we performed *ex vivo* knockdown studies using siRNA against *Adig* in mouse PGAT explants. We found that knockdown of *Adig* decreased *Lep* expression by 26% (*P*=4 × 10^−4^) and leptin secretion by 23% (*P*=0.003), consistent with a causal role for *ADIG* in control of circulating leptin levels (Fig. 4a,b and Supplementary Fig. 2C,D).

*ADIG* is located ∼116 kb from the rs6071166 variant and encodes a cytoplasmic adipocyte protein adipogenin, that is, similar to leptin, highly and specifically expressed in the adipose tissue^28^,^29^,^30^ and upregulated by treatment with insulin and glucose^30^. *Adig* expression is also strongly upregulated in murine 3T3-L1 preadipocytes during *in vitro* differentiation into adipocytes^28^,^29^. Two studies have investigated the effect of *Adig* knockdown on the differentiation of 3T3-L1 cells and expression of *Pparγ2*, a master regulator of adipocyte differentiation, but with conflicting results; whereas the first study found *Adig* knockdown to block adipocyte differentiation and decrease *Pparγ2* expression^28^, a later study found no similar changes^30^. When we measured *Pparγ2* expression following *Adig* knockdown in PGAT explants containing mature adipocytes, we did not see a change as compared with controls (Supplementary Fig. 2F).

### Common variation in *GCKR* regulates leptin levels

Variants (*r*^2^≥0.9 with our lead SNP rs780093) of the leptin-associated locus in *GCKR* have previously shown genome-wide significant associations with more than 25 metabolic traits; the leptin-increasing allele has been associated with increased fasting glucose and fasting insulin but decreased 2-h glucose and higher high-density lipoprotein cholesterol, and lower total cholesterol, low-density lipoprotein cholesterol, triglycerides, C-reactive protein and circulating uric acid levels, among others (Supplementary Table 11). The *GCKR* gene encodes a regulatory protein in the liver that inhibits the activity of glucokinase, the enzyme responsible for regulating the uptake, metabolism and storage of circulating glucose^31^. A putative causal variant in this gene is the common nonsynonymous Pro446Leu variant (rs1260326), for which rs780093 acts as a good proxy (*r*^2^=0.9). Carriers of the glucose-lowering Leu allele have a reduced ability to sequester and inhibit glucokinase and a blunted response to fructose 6-phosphate, both of which favour the generation of free and active cytoplasmic glucokinase^32^.

The mechanisms that might link changes in *GCKR* function to leptin levels are not known. As insulin increases leptin secretion from adipocytes^33^ and the *GCKR* locus is strongly associated with circulating levels of insulin^34^, the association of the *GCKR* locus with leptin levels could be the consequence of the GCKR locus' effect on insulin levels. The leptin-increasing allele of the rs780093 variant was significantly associated with higher levels of fasting insulin in studies included in our stage 2 meta-analyses (*P*=2 × 10^−5^, *N*=8,953). To test whether insulin mediated the association of rs780093 with circulating leptin levels, we analysed the association of rs780093 with leptin, while adjusting for fasting insulin levels. Although the effect size was somewhat attenuated, the association of rs780093 with BMI-adjusted leptin levels remained significant after adjustment for fasting insulin (*β*=0.047, *P*=2 × 10^−4^ versus *β*=0.034, *P*=0.004 before and after the adjustment, respectively), suggesting that the association of the *GCKR* locus with leptin is at least in part independent of effects on insulin levels.

Although *GCKR*'s function renders it a potential candidate among the genes in this region, *cis*-eQTL analyses showed association of the leptin-increasing allele of rs780093 with increased expression of the nearby *IFT172* in the liver (*P*=7 × 10^−30^), omental fat (*P*=6 × 10^−64^) and subcutaneous fat (*P*=3 × 10^−52^) (Supplementary Table 9). The rs780093 variant is, however, only in moderate LD (*r*^2^=0.4) with the peak SNP influencing *IFT172* expression in the region and the peak SNP remained significantly associated with *IFT172* expression after adjustment for rs780093, whereas the association of rs780093 was abolished after adjustment for the peak SNP (Supplementary Table 9).

Because of the observations in human tissues, we examined *Ift172* in the mouse explant model. *Ift172* was not highly expressed in mouse PGAT or SCAT and levels were not upregulated by high-fat diet (Fig. 3e,f). *Ift172* was, however, upregulated in the liver under high-fat diet feeding (Supplementary Fig. 7E). Knockdown of *Ift172* in PGAT explants decreased *Lep* mRNA expression by 22% (*P*=0.02), but did not decrease leptin protein secretion (*P*=0.6) (Fig. 4a,b and Supplementary Fig. 3C,D). *IFT172* is known to play a major role in assembly and maintenance of primary cilia that act as critical signalling hubs for cellular pathways during development^35^. Knockout of *Ift* genes in central neurons causes obesity in mice^36^ and obesity is a clinical feature in two human ciliopathic syndromes, the Alström and Bardet–Biedl syndromes^37^,^38^. In the hypothalamus, alterations in the function of the primary cilium lead to impaired leptin signalling^39^. Therefore, we cannot exclude a role for *IFT172* in the regulation of circulating leptin levels.

Another nearby gene, *MpV17* mitochondrial inner membrane protein, is a potential candidate in the region based on its expression in mice fed chow or high-fat diet; *Mpv17* expression was increased by high-fat diet, in a manner similar to *Lep* (Fig. 3c,d). However, knockdown of *Mpv17* did not change *Lep* mRNA expression (*P*=0.2) or leptin secretion (*P*=0.2) by PGAT explants (Fig. 4a,b and Supplementary Fig. 4C,D), suggesting that the involvement of *MPV17* in leptin regulation is unlikely.

### Locus near *CCNL1* regulates leptin levels and birth weight

The leptin-decreasing allele of rs900400, located 67 kb upstream from *CCNL1* (Fig. 2d), was previously reported for its association with lower birth weight^40^. This cross-phenotype association could indicate a mechanism that is shared between birth weight and leptin levels in adulthood. Fetal adipose tissue is capable of producing leptin^41^ and fetal leptin levels are correlated with fetal fat mass^42^,^43^. Placenta provides an additional source of leptin for the fetus, however, and it has been suggested that leptin could mediate fetal growth^44^,^45^. Assuming that leptin levels track from birth through adulthood, increased leptin levels could drive the association of the *CCNL1* locus with birth weight. Other studies suggest that leptin production is decreased in cultured adipocytes from men born with a low birth weight^46^. Therefore, the association of the *CCNL1* locus with leptin levels in adulthood could be mediated by its association with birth weight.

Although *CCNL1* is the nearest gene to rs900400, our *cis*-eQTL analyses identified rs900400 as the variant most significantly associated with the expression of another nearby gene, *TIPARP* (Supplementary Table 9). The *TIPARP* gene encodes a poly (ADP-ribose) polymerase involved in DNA repair. The leptin-increasing allele of rs900400 was associated with lower *TIPARP* expression in omental fat (3 × 10^−30^) and subcutaneous fat (*P*=7 × 10^−58^) (Supplementary Table 9). *Tiparp* was also implicated as a causal gene by our expression analysis of mouse adipose tissue and its expression was increased in SCAT and liver in mice fed with high-fat diet (Fig. 3h and Supplementary Fig. 7G). Knockdown of *Tiparp* in mouse PGAT explants did not, however, significantly alter the expression of *Lep* mRNA (*P*=0.7) or leptin secretion (*P*=0.8) (Fig. 4a,b and Supplementary Fig. 5D,E). Although we attempted to use SCAT for explant knockdown studies, high intra-depot variability compromised this approach. Interestingly, stimulation of the explants with insulin and dexamethasone increased explant expression of *Tiparp* by 50% (*P*=0.003) over incubation in basal media alone, in a manner similar to *Lep* expression (Fig. 4c and Supplementary Fig. 5A). Collectively, although *TIPARP* remains a putative causal gene within the locus near *CCNL1*, further evidence is required to confirm its role in the regulation of circulating leptin levels.

### *COBLL1* or *GRB14* may regulate leptin levels

The intronic rs6738627 variant in *COBLL1* (Fig. 2e) did not reach genome-wide significance for the association with leptin levels (Fig. 1 and Table 1). However, as previous GWAS have shown robust associations of the leptin-increasing allele with a lower WHR_adjBMI_^19^, we chose to take it forward for follow-up analyses, to examine the role of leptin levels in the previous associations.

Look-ups in data from genetic consortia showed a strong association of the leptin-increasing allele of rs6738627 with higher body fat percentage (*P*=2 × 10^−8^, *n*=76,338; Supplementary Table 7). As reported previously, the rs6738627 variant was also strongly associated with decreased WHR_adjBMI_ (*P*=2 × 10^−8^, *n*=174,672; Supplementary Table 7), suggestive of a preferential gluteal rather than abdominal fat storage, which may contribute to the association of rs6738627 with increased leptin levels^47^.

In PGAT and SCAT expression analyses in mice, we found an upregulation of *Cobll1* in high-fat diet-fed mice in both depots (Fig. 3i,j). Although knockdown of *Cobll1* in the perigonadal explants did not influence *Lep* mRNA expression (*P*=0.2), it did decrease leptin protein secretion by 16% (*P*=3 × 10^−4^, Fig. 4a,b), suggesting a potential causal role for *Cobll1*. In addition, stimulation of explants with insulin and dexamethasone increased explant expression of *Cobll1* by 78% (*P*=0.004) over incubation in basal media alone (Fig. 4c and Supplementary Fig. 6A). *COBLL1* is known to be involved in neural tube formation^48^, but its possible functions in adipose tissue are unknown.

In human eQTL analyses, the leptin-increasing allele of the *COBLL1* locus showed an association with lower expression of *GRB14* in omental fat (*P*=5 × 10^−12^) and subcutaneous fat (*P*=3 × 10^−5^) (Supplementary Table 9). We did not, however, find high expression of *Grb14* in PGAT or SCAT explants from mice and the levels were not regulated by high-fat diet feeding (Fig. 3i,j). The protein product of *GRB14* is the growth factor receptor-bound protein 14 that binds directly to the insulin receptor and inhibits insulin signalling^49^. The adipose tissue expression of *GRB14* may play a role in regulating insulin sensitivity^50^. Grb14-deficient mice exhibit improved glucose tolerance, lower circulating insulin levels and increased incorporation of glucose into glycogen in the liver and skeletal muscle^51^. Both *COBLL1* and *GRB14* are thus possible candidates to account for the association of the *COBLL1* locus with leptin levels.

### Enrichment with pathways and regulatory elements

We used the Data-driven Expression Prioritized Integration for Complex Traits (DEPICT) software^52^ to identify enrichment of gene sets and pathways across loci reaching *P*<1 × 10^−5^ for association with leptin levels. However, none of our findings reached the false discovery rate threshold of 5% (Supplementary Tables 12–17). Next, we used the Gene Relationships Across Implicated traits (GRAIL) tool^53^ to identify genes near the leptin-associated loci having similarities in the text describing them within published article abstracts. However, no statistically significant results were found in these analyses either (Supplementary Tables 18 and 19). Finally, we used the Uncovering Enrichment Through Simulation method^54^ to test for the overall enrichment of leptin-associated loci reaching *P*<10^−5^ with ChromHMM annotations for adipose and brain tissues available from the Roadmap Epigenomics Project^24^. However, we did not find significant enrichment of our leptin-associated loci in any chromatin states once corrected for multiple testing (Supplementary Table 20). The lack of significant findings may be due to the small number of loci identified and the limited knowledge available on leptin-regulating pathways in adipose tissue.

### Established adiposity loci and leptin

Circulating leptin levels correlate closely with BMI and other measures of adiposity^3^. The most recent GWAS meta-analysis for BMI, including nearly 340,000 individuals, identified 97 loci that reached genome-wide significance^22^. Of the 97 BMI-increasing loci, 89 showed a directionally concordant association with increased BMI-unadjusted leptin levels (*P*_binomal_=2 × 10^−18^), of which 25 reached nominal significance (Supplementary Table 21). Previous GWAS of extreme and early-onset obesity have identified 12 genome-wide significant loci^55^,^56^,^57^,^58^. Of these, ten showed a directionally consistent association with increased BMI-unadjusted leptin levels (*P*_binomal_=0.04), of which five reached nominal significance (Supplementary Table 21).

We also examined leptin associations for 49 loci identified in GWAS for WHR_adjBMI_, a measure of body fat distribution independent of overall adiposity^19^. Of the 49 WHR_adjBMI_-increasing loci, only 24 showed a directionally concordant association with increased BMI-adjusted leptin levels (Supplementary Table 22). As the distribution of body fat differs between men and women, we also examined the leptin associations for the 49 WHR_adjBMI_ loci in men and women separately. There was no enrichment of leptin associations in either of the sexes, with 27 loci showing a directionally concordant association with increased leptin levels in men and 20 loci in women (Supplementary Table 22).

## Discussion

In a meta-analysis of genetic association data in up to 52,126 individuals, we identified 5 common loci associated with circulating leptin levels. In addition, a locus near *COBLL1*, previously identified for association with a lower WHR_adjBMI_^19^, reached *P*=1 × 10^−6^ for association with increased leptin levels. Even though leptin correlates strongly with adiposity, we did not identify loci previously associated with BMI, other than *FTO*, despite having a sample size similar to early GWAS meta-analyses of BMI that identified multiple loci^59^. On the contrary, five of the six loci we identified were associated with leptin independently of BMI or body fat percentage. Our findings indicate that genetic mechanisms not influencing adiposity may have an important role in the regulation of circulating leptin levels.

Our strongest adiposity-independent leptin signal was near *LEP*, but we also identified leptin-associated variants in four other genomic loci, providing evidence that mechanisms other than those that involve *LEP per se* may regulate leptin production and release from adipose tissue. In one of these loci, near *SLC32A1*, our knockdown studies indicated a role for *adipogenin*, a gene involved in the regulation of adipocyte differentiation^28^,^29^. Although adipogenin was identified as a potent regulator of adipogenesis a decade ago^28^,^29^, our results provide the first evidence linking this function to leptin regulation. We anticipate that our findings will motivate and inform eventual testing of *Adig* by transgenic manipulation in mice.

No clear effect on leptin production was seen following knockdown of candidate genes in the *GCKR* and *CCNL1* loci, which may indicate that the gene implicated by position plays no role in the phenotype, or that the effect was undetectable in our experimental conditions. Alternatively, the association with leptin levels may be explained by effects of non-coding elements on other genes outside the implicated genetic interval, or by inter-species differences. Furthermore, although adipose tissue is the most direct contributor to circulating leptin levels, the effect of the causal gene may be conveyed by another tissue; leptin production and secretion are influenced by insulin, catecholamines and other hormones, as well as paracrine effects of local inflammatory cells on adipocytes^60^.

Although the locus near *SLC32A1* had not been identified previously for association with other traits, the leptin-associated loci in/near *GCKR*, *CCNL1* and *COBLL1* have been associated with multiple obesity-related and metabolic traits^19^,^20^,^34^,^40^,^61^. These cross-phenotype associations may either reflect pleiotropy, where a gene product influences multiple traits, and/or mediation effects, where one phenotype is causally related to a second phenotype. For example, the association of the pleiotropic *GCKR* locus with leptin levels may be partly mediated through *GCKR*'s role in the regulation of glucose homeostasis and insulin levels^34^,^61^, which may influence leptin production and secretion in adipose tissue^33^. The *COBLL1* locus is strongly associated with decreased WHR_adjBMI_, indicative of a preferential accumulation of gluteal subcutaneous fat, which may contribute to the observed association with circulating leptin levels^47^. The identification of the birth weight locus, *CCNL1*, as a leptin-regulating locus may provide an intriguing link between leptin regulation and fetal growth, albeit such a link remains to be more firmly established^45^.

Unravelling the polygenic basis of leptin production could provide opportunities for targeted leptin supplementation in obese individuals. Although leptin therapy is an efficient weight-loss treatment for obese individuals with congenital leptin deficiency, the beneficial effects of leptin supplementation do not translate to all obese patients^62^. Sensitivity to changes in circulating concentration of leptin may be enhanced at very low values^11^ where a relatively small increase in leptin production may be sensed by the homeostatic feedback system that controls energy balance. As a substantial minority of individuals with common forms of obesity, not associated with leptin mutations, have relatively low levels of circulating leptin^63^, augmenting leptin levels in this subgroup could be therapeutically worthwhile. Identification of leptin-regulating loci may provide new tools for identifying obese individuals with susceptibility to low leptin levels and who may benefit of leptin treatment.

In 2010, Sun *et al.*^64^ identified two common non-synonymous SNPs in the leptin receptor (*LEPR*) gene associated with leptin receptor levels. Leptin receptor plays an essential role in mediating the physiological effects of leptin. Although some studies have described abnormally high circulating leptin levels in carriers of rare *LEPR* mutations^65^, others have not^66^. We did not find association between *LEPR* variants and circulating leptin levels, suggesting that common variants in *LEPR* are not important regulators of circulating leptin levels.

Our meta-analyses were limited by the number of available studies with leptin data, imputation by HapMap reference panel for autosomal chromosomes and the fact that we examined additive effects only. In addition, we corrected for adiposity by adjusting for BMI, which is a heterogeneous measure of adiposity as it does not account for individual differences in body fat and lean mass. Future discovery efforts in extended sample sizes based on genome-wide imputation of 1000 Genomes reference panels, which include X and Y chromosomes and which also test for recessive and dominant inheritance, will allow for the discovery of more and lower-frequency variants, and for refining association signatures of already established leptin-associated loci.

In summary, we identified six genetic loci associated with circulating leptin levels, of which five showed associations independently of adiposity. Our findings represent a step forward in the understanding of biological mechanisms regulating leptin production in adipose tissue and open new avenues for examining the influence of variation in leptin levels on adiposity and metabolic health.

## Methods

### Main analyses

*Study design*. We conducted a two-stage meta-analysis to identify leptin-associated loci in adults of European ancestry. In stage 1, we performed a meta-analysis of 23 GWAS (*n*=32,161) (Supplementary Table 1) for BMI-unadjusted and BMI-adjusted circulating levels of leptin. Stage 2 included 13 additional studies (*n*=19,979), which provided either *de novo* or *in silico* data for the lead SNPs of the independent loci reaching *P*<1 × 10^−6^ in Stage 1 (Supplementary Table 5). Secondary meta-analyses were conducted in men (*n*=13,363) and women (*n*=18,698) separately, and with adjustment for body fat percentage (assessed by dual-energy X-ray absorptiometry or bioimpedance analysis) instead of BMI (*n*=18,980). The study-specific descriptive statistics are presented in Supplementary Table 23.

*Stage 1 genome-wide association analyses*. Following study-specific quality control measures, the genotype data were imputed using the HapMap Phase II CEU reference panel (Supplementary Table 24). Directly genotyped and imputed variants were then tested for association with logarithmically transformed leptin (ng ml^−1^), adjusting for age, age^2^ and any necessary study-specific covariates (for example, genotype-derived principal components) in a linear regression model. The analyses were performed with and without additional adjustment for BMI. In studies that had assessed body fat percentage with bioimpedance analysis or dual-energy X-ray absorptiometry, additional analyses were performed with adjustment for body fat percentage. The analyses were performed in men and women separately. In studies that included closely related individuals, regression coefficients were also estimated in the context of a variance component model that modelled relatedness in men and women combined, with sex as a covariate.

Before performing meta-analyses on the data from individual studies, SNPs with poor imputation quality scores (*r*^2^-hat <0.3 in MACH, proper-info <0.4 in IMPUTE, INFO <0.8 in PLINK) or with a minor allele count <6 were excluded for each study (Supplementary Table 24). The genotype data for the leptin-associated lead SNPs was of high quality with a median imputation score of ≥0.94 (Supplementary Table 26). The fifth percentile for all SNPs was ≥0.80, except for the previously established rs900400 SNP near *CCNL1*.

All individual GWAS were genomic control corrected before meta-analyses. Individual study-specific genomic control values ranged from 0.977 to 1.051. Fixed effects meta-analyses were then conducted using the inverse variance-weighted method implemented in METAL. The genomic control values for the meta-analysed results were 1.050, 1.026 and 1.022 in the BMI-unadjusted meta-analyses of all individuals, men and women, and 1.046, 1.022 and 1.015 in the BMI-adjusted meta-analyses, respectively. Using the LD score regression method^67^ in the Stage 1 meta-analyses suggests that the observed inflation is not due to population substructure. The regression intercept, which estimates inflation after removing polygenic signals, was 0.994 for BMI-unadjusted and 1.004 for BMI-adjusted meta-analyses of men and women combined.

*Selection of SNPs for follow-up*. We used a pairwise distance criterion of ±500 kb and *r*^2^<0.1 between SNPs that reached *P*<10^−6^ in the meta-analysis of BMI-adjusted or -unadjusted meta-analysis of leptin levels in Stage 1 in men and women combined or separately, to select loci forward for follow-up in Stage 2. We tested the association of the lead SNPs in up to 19,929 adults of white European ancestry in Stage 2.

*Stage 2 follow-up of the loci reaching *P*<10^−6^ in Stage 1*. Association results were obtained from 13 studies that had not been included in the Stage 1 meta-analyses (Supplementary Table 5). Samples and SNPs that did not meet the quality control criteria defined by each individual study were excluded. Minimum genotyping quality control criteria were defined as Hardy–Weinberg equilibrium *P*>10^−7^, call rate >90% and concordance >99% in duplicate samples in each of the follow-up studies.

We tested the association between the SNPs and leptin in each Stage 2 study using approaches similar to those described for the Stage 1 studies. We subsequently performed a meta-analysis of *β*-coefficients and s.e. from Stage 2 using the inverse variance fixed effects method. The final meta-analysis combined GWAS results from Stage 1 with the Stage 2 results. The conventional *P*-value threshold of <5 × 10^−8^ in the combined Stage 1 and Stage 2 meta-analysis was used to determine genome-wide significance.

### Identifying genes and biological pathways at associated loci

*Cross-trait look-ups*. To further examine the relationship between the leptin-associated loci and anthropometric and metabolic parameters, we acquired association results for the loci in or near *LEP*, *SLC32A1*, *GCKR*, *CCNL1* and *COBLL1* from nine GWAS meta-analysis consortia: ADIPOGen (BMI-adjusted adiponectin), BCGC (body fat percentage), DIAGRAM (type 2 diabetes), Early growth genetics (birth weight, early-onset obesity), ICBP (systolic and diastolic blood pressure), GIANT (height, BMI, waist–hip ratio adjusted for BMI), GLGC (circulating levels of high-density lipoprotein cholesterol, low-density lipoprotein cholesterol, triglycerides and total cholesterol), MAGIC (fasting glucose, fasting insulin) and ReproGen (age at menarche) (Supplementary Table 7).

*National Human Genome Research Institute GWAS Catalog look-ups*. To identify the associations of the leptin-associated loci in published GWAS, we extracted previously reported GWAS associations within 500 kb and *r*^2^>0.7 with any of the lead leptin-associated SNPs, from the GWAS Catalog of the National Human Genome Research Institute ( www.genome.gov/gwastudies) (Supplementary Table 11).

*Overlap with functional regulatory elements*. We used the Uncovering Enrichment Through Simulation method to combine the leptin association data with the Roadmap Epigenomics Project segmentation data^54^. The pipeline chose 10,000 sets of random SNPs among HapMap2 SNPs with a MAF>0.05 and that matched the original input SNPs based on proximity to a transcription start site and the number of LD partners (*r*^2^>0.8 in individuals of European ancestry in the 1000 Genomes Project). The LD partners were combined with their original lead SNP to create 10,000 sets of matched random SNPs and their respective LD partners. These sets were intersected with the 15-state ChromHMM data from the Roadmap Epigenomics Project and resultant co-localizations were collapsed from total SNPs down to loci, which were then used to calculate an empirical *P*-value when comparing the original SNPs with the random sets. In addition to examining overall enrichment for all leptin-associated loci combined, we examined the variant-specific overlap with regulatory elements for each of the leptin-associated index SNPs and variants in strong LD (*r*^2^>0.8).

*Expression quantitative trait loci*. We examined the *cis*-associations of the leptin-associated loci with the expression of nearby genes in the lymphocytes, skin, liver, omental fat, subcutaneous fat and brain tissue (Supplementary Table 8). Conditional analyses were performed by including both the leptin-associated SNP and the most significant *cis*-associated SNP in the association model for a given transcript. To minimize the potential for false positives, we only considered associations that reached study-specific Bonferroni-corrected significance threshold (*P*<0.05/(total number of transcripts tested)).

### Pathway analyses

*GRAIL analyses:* We used GRAIL to identify genes near the leptin-associated loci having similarities in the published scientific text using PubMed abstracts as of December 2006 (ref. 53). The leptin loci were queried against HapMap release 22 for the European panel and we controlled for gene size.

*DEPICT analyses:* We used DEPICT to identify the most probable causal gene at a given associated locus, reconstituted gene sets enriched for BMI associations, and tissues and cell types in which genes from associated loci are highly expressed^52^. We clumped GWAS-based meta-analysis summary statistics using 500 kb flanking regions, LD *r*^2^>0.1 and excluded SNPs with *P*≥1 × 10^−5^. HapMap Project Phase II CEU genotype data were used to compute LD and genomic coordinates were defined by genome build GRCh37.

### Knockdown of genes in mouse adipose tissue explants

*Materials*. Expression analyses were performed on PGAT and SCAT from 4-month-old C57BL/6J mice (derived from Jackson, Stock number 000664) fed chow (Purina PicoLab 5058) or high-fat diet (Research Diets, Inc., D12492i, 60% kcal from fat), to increase adiposity and circulating leptin levels. We measured expression of genes located ±100 kb of each lead variant or genes including SNPs with *r*^2^>0.4 with the lead variant. *Tiparp* was included after its identification by eQTL analysis, and *Ucn* and *Mpv17* were included based on their proximity to variants with *r*^2^>0.4 with the lead variant.

For knockdown experiments, 15-week-old male C57BL/6J mice fed high-fat diet *ad libitum* starting at 6 weeks of age were purchased from Jackson Laboratory (Stock Number 380050, Bar Harbor, ME). Animals were maintained at Columbia University animal facility for up to an additional 5 weeks until they reached ∼30% fat mass as determined by time-domain NMR (Minispec Analyst AD; Bruker Optics, Silberstreifen, Germany).

Mice were maintained at an ambient temperature of 22 °C–24 °C with a 12-h dark–light cycle (lights on at 0700, h) in a pathogen-free barrier facility. The protocol was approved by the Columbia University Institutional Animal Care and Use Committee.

*Electroporation and culture of adipose tissue explants*. Non-fasted mice were killed at around 20 weeks of age at ∼1000, h. PGAT was dissected and minced into 1- to 2-mm fragments. These fragments were evenly distributed into three replicates per control or knockdown condition. Approximately 7–11 fragments were added per well (for a total amount of ∼80 mg tissue) in 12-well culture dishes containing 1 ml M199 with Antibiotic-Antimycotic (Anti-Anti, 5 × ; Invitrogen). Following a 20-min incubation in 5 × Anti-Anti media, tissue fragments were washed twice with 1 ml PBS and then transferred to 4 mm Gene Pulser cuvettes (Bio-Rad) and electroporated in 400 μl PBS with 1 nmol siRNA against *Lep*, *Adig*, *Ift172*, *Mpv17*, *Tiparp* or *Cobll1* (Stealth siRNA, Invitrogen). Non-targeting sequences were used as negative controls (Invitrogen). Electroporation was performed with a Gene Pulser XceII (Bio-Rad) using 50 V, 10^2^ wave pulses, with a pulse length of 30 ms and 0.1 ms between pulses^68^. The tissue fragments were subsequently cultured at 37 °C in 5% CO_2_ in 12-well plates for 20 h in basal media consisting of M199 media with 10% fetal bovine serum (Invitrogen) plus 1 × Anti-Anti before stimulation for 12 h with basal media plus 7 nM insulin and 25 nM dexamethasone (both from Sigma), to maintain leptin expression in the explants at levels comparable to those of *in vivo* tissues^69^. Knockdown was considered successful if candidate expression was decreased by ≥30%. The effect of insulin and dexamethasone on expression of candidate genes was determined using the same mincing and culturing strategy without electroporation.

*Measuring mRNA levels and leptin and adiponectin secretion*. Total RNA was isolated using TRIzol reagent (Invitrogen) and reverse transcribed using Transcriptor First Strand cDNA Synthesis Kit (Roche) using both OligoDT and random hexamer primers. Lightcycler 480 SYBR Green I Master was used for quantitative PCR assays (Roche). Expression of murine homologues of candidate genes in PGAT and SCAT was determined using the 2(−ΔΔC(T)) method^70^. Gene expression in the knockdown experiments was calculated by Lightcycler 480 software (Roche) based on a standard curve. Primers used are listed in Supplementary Table 25. Culture media was collected from the same samples used for RNA analyses. Following the 12 h insulin/dexamethasone stimulation, secreted leptin and adiponectin were measured using the Perkin-Elmer AlphaLISA kits for mouse leptin and adiponectin (according to the manufacturer's protocol). Not all samples were included for adiponectin measurement due to the discontinuation of the AlphaLISA kit by Perkin-Elmer.

*Statistics*. Each gene knockdown was tested on tissue from 5 to 13 different mice, as indicated. Control and knockdown samples from each mouse were treated as matched pairs. Each data point represents the mean of three replicates from a single mouse. Differences between control and knockdown conditions were calculated by two-way repeated measures analysis of variance using GraphPad Prism 6. *P*-values <0.05 were considered significant.

## Additional information

**How to cite this article:** Kilpeläinen, T. O. *et al.* Genome-wide meta-analysis uncovers novel loci influencing circulating leptin levels. *Nat. Commun.* 7:10494 doi: 10.1038/ncomms10494 (2016).

## Supplementary Material

Supplementary Information Supplementary Figures 1-7, Supplementary Tables 1-26, Supplementary Note 1 and Supplementary References

## Figures and Tables

**Table 1:**
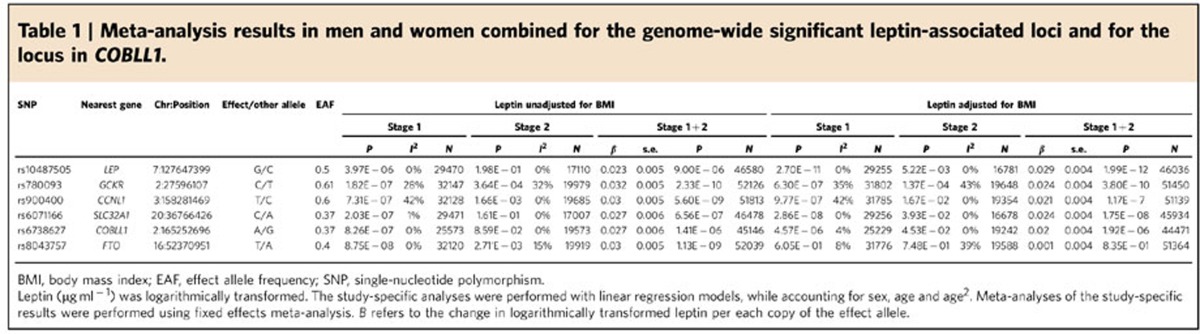
Meta-analysis results in men and women combined for the genome-wide significant leptin-associated loci and for the locus in *COBLL1*.
