# Supplementary material for: Genome-wide meta-analysis uncovers novel loci influencing circulating leptin levels
Source: Nat Commun. 2016 Feb 1;7:10494. doi: 10.1038/ncomms10494 (PMC4740377; doi:10.1038/ncomms10494)
Supplement: Supplementary Information — Supplementary Figures 1-7, Supplementary Tables 1-26, Supplementary Note 1 and Supplementary References [file ncomms10494-s1.pdf]

Supplementary Figures

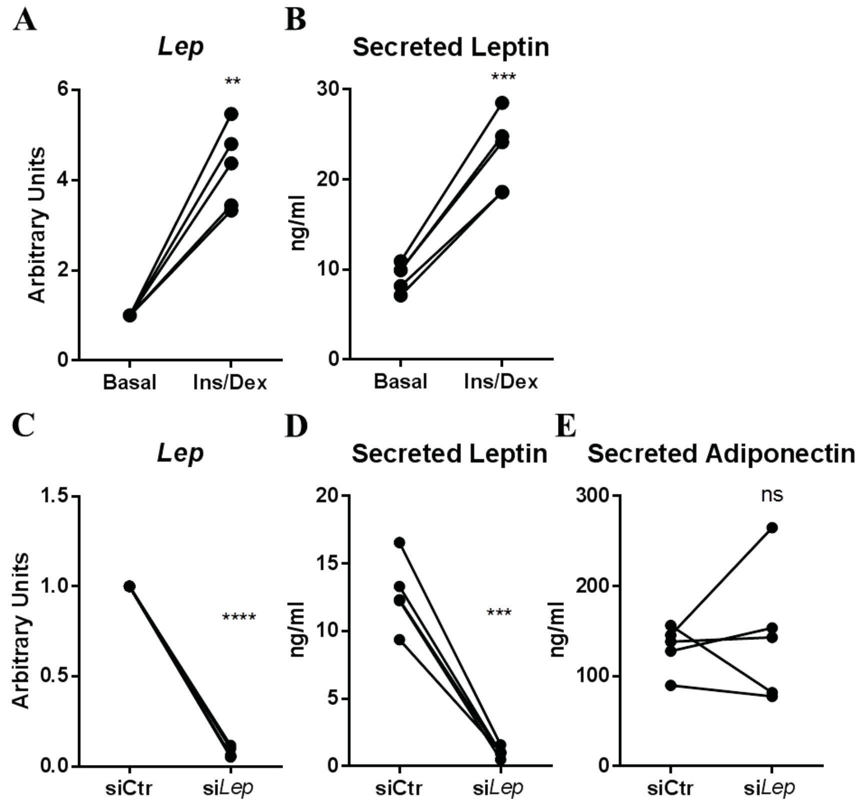

Supplementary Fig. 1. Effects of *Lep* knockdown on leptin transcription and secretion in perigonadal adipose tissue explants from mice fed with high fat diet. *Lep* transcription (**A**) and secretion (**B**) by explants in the basal or Ins/Dex stimulated state. N=5 mice per group. *Lep* knockdown (**C**) decreased LEP secretion (**D**) following stimulation with insulin and dexamethasone for 12 hours. Adiponectin secretion (**E**) remained unchanged. N=5 mice per group. Each point represents the average of 3 samples. 2 way repeated measures ANOVA. *p<0.05, **p<0.01, ***p<0.001, ****p<0.0001.

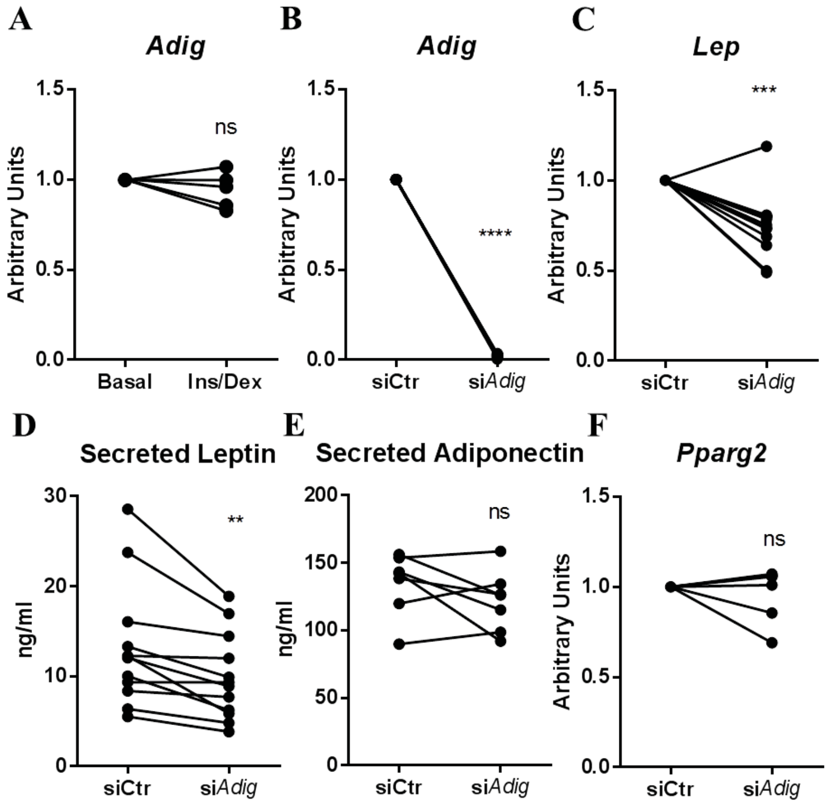

Supplementary Fig. 2 Effects of *Adig* knockdown on leptin transcription and secretion in perigonadal adipose tissue explants from mice fed with high fat diet. *Adig* expression (**A**) by explants in the basal or Ins/Dex stimulated state. N=5 mice per group. *Adig* knockdown (**B**) decreased both *Lep* expression (**C**) and leptin secretion (**D**) following stimulation with insulin and dexamethasone for 12 hours. N=12 mice per group. Adiponectin secretion (**E**) remained unchanged. N=7 mice per group. *Pparg2* expression (F) was unchanged as well. N=6 mice per group. Each point represents the average of 3 samples. 2 way repeated measures ANOVA. *p<0.05, **p<0.01, ***p<0.001, ****p<0.0001.

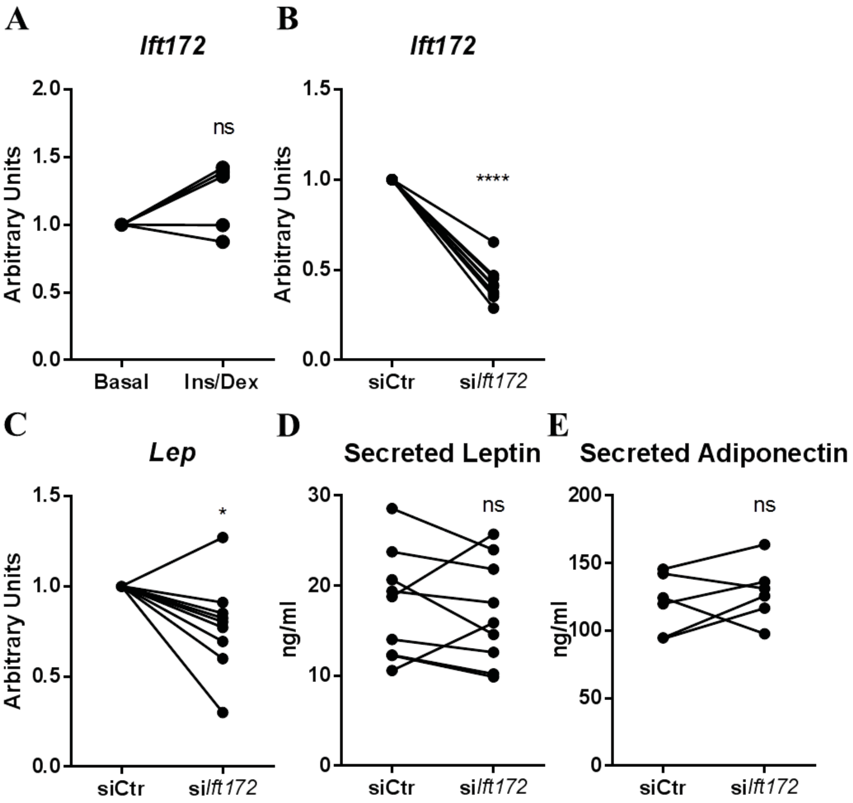

Supplementary Fig. 3. Effects of *Ift172* knockdown on leptin transcription and secretion in perigonadal adipose tissue explants from mice fed with high fat diet. *Ift172* expression (**A**) by explants in the basal or Ins/Dex stimulated state. N=5 mice per group. *Ift172* knockdown (**B**) decreased *Lep* expression (**C**) but did not change leptin secretion (**D**) following stimulation with insulin and dexamethasone for 12 hours. N=9 mice per group. Adiponectin secretion (**E**) remained unchanged. N=6 mice per group. Each point represents the average of 3 samples. 2 way repeated measures ANOVA. *p<0.05, **p<0.01, ***p<0.001, ****p<0.0001.

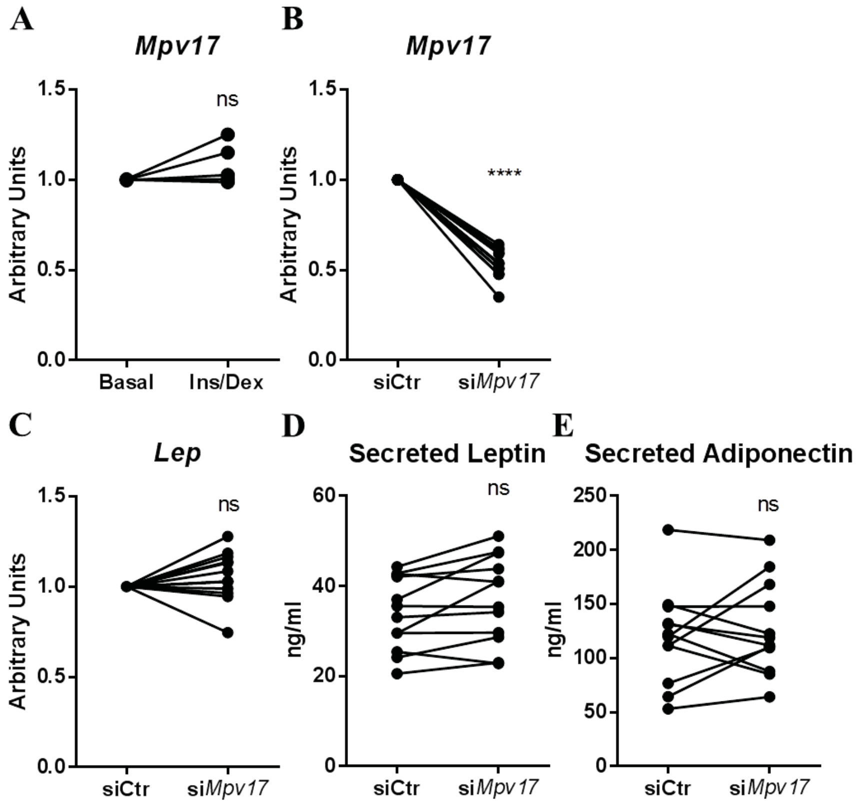

Supplementary Fig. 4. Effects of *Mpv17* knockdown on leptin transcription and secretion in perigonadal adipose tissue explants from mice fed with high fat diet. *Mpv17* expression (**A**) by explants in the basal or Ins/Dex stimulated state. N=5 mice per group. *Mpv17* knockdown (**B**) did not change *Lep* expression (**C**) or secretion (**D**) following stimulation with insulin and dexamethasone for 12 hours. Adiponectin secretion (**E**) remained unchanged. N=12 mice per group. Each point represents the average of 3 samples. 2 way repeated measures ANOVA. *$p<0.05$, **$p<0.01$, ***$p<0.001$, ****$p<0.0001$.

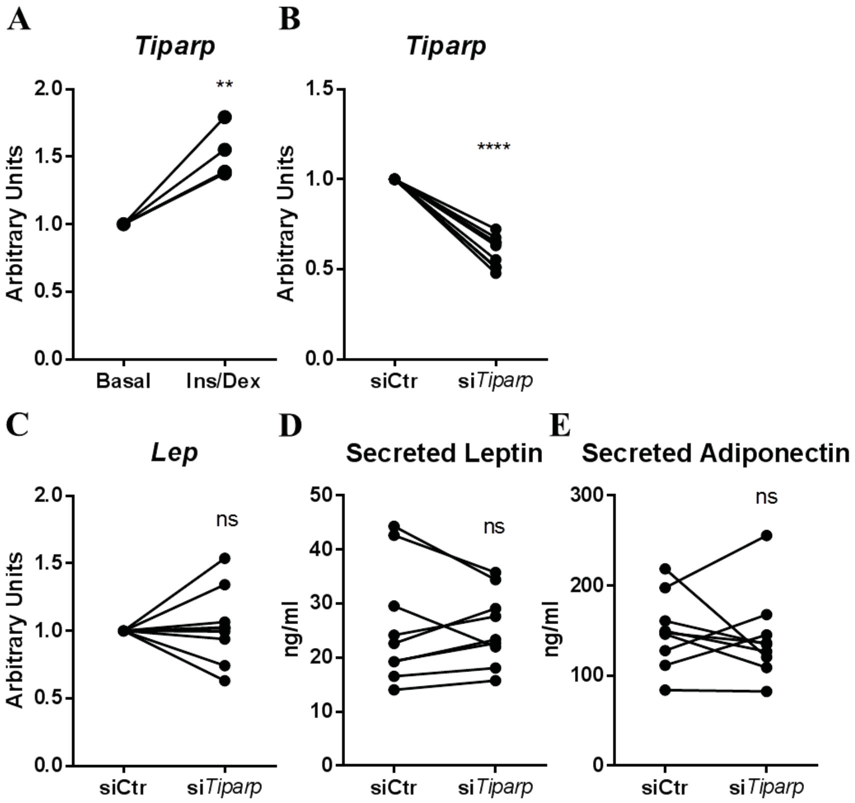

Supplementary Fig. 5. Effects of *Tiparp* knockdown on leptin transcription and secretion in perigonadal adipose tissue explants from mice fed with high fat diet. *Tiparp* expression (**A**) by explants in the basal or Ins/Dex stimulated state. N=5 mice per group. *Tiparp* knockdown (**B**) did not change *Lep* expression (**C**) or secretion (**D**) following stimulation with insulin and dexamethasone for 12 hours. Adiponectin secretion (**E**) remained unchanged. N=9 mice per group. Each point represents the average of 3 samples. 2 way repeated measures ANOVA. *p<0.05, **p<0.01, ***p<0.001, ****p<0.0001.

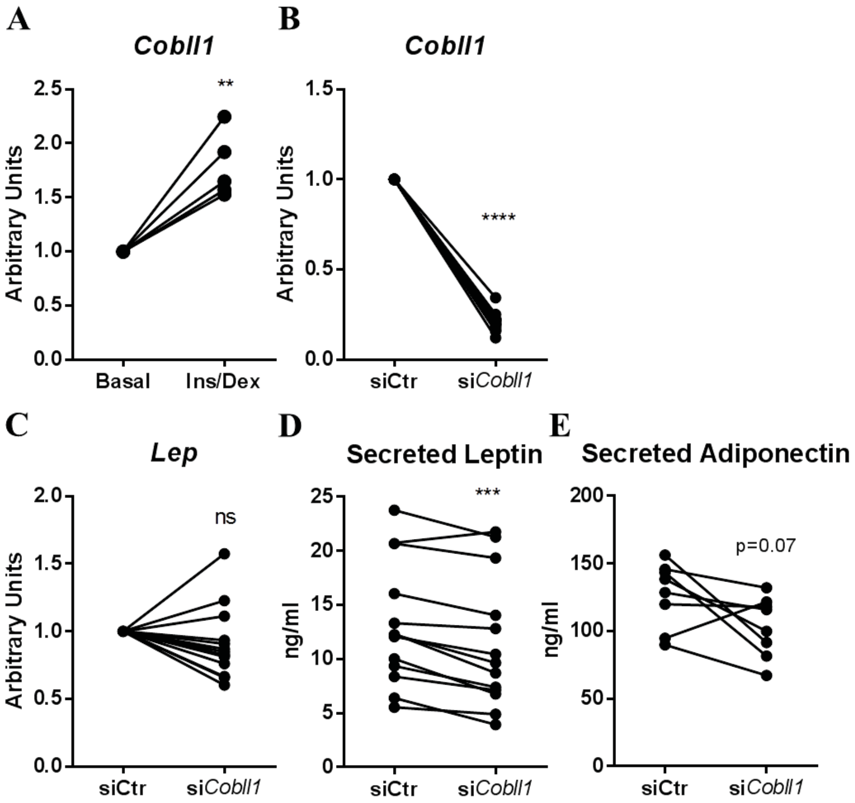

Supplementary Fig. 6. Effects of *Cobll1* knockdown on leptin transcription and secretion in perigonadal adipose tissue explants from mice fed with high fat diet. *Cobll1* expression (**A**) by explants in the basal or Ins/Dex stimulated state. N=5 mice per group. *Cobll1* knockdown (**B**) did not change *Lep* expression (**C**) but decreased Lep secretion (**D**) following stimulation with insulin and dexamethasone for 12 hours. N=13 mice per group. Adiponectin secretion (**E**) remained unchanged. N=8 mice per group. Each point represents the average of 3 samples. 2 way repeated measures ANOVA. *p<0.05, **p<0.01, ***p<0.001, ****p<0.0001.

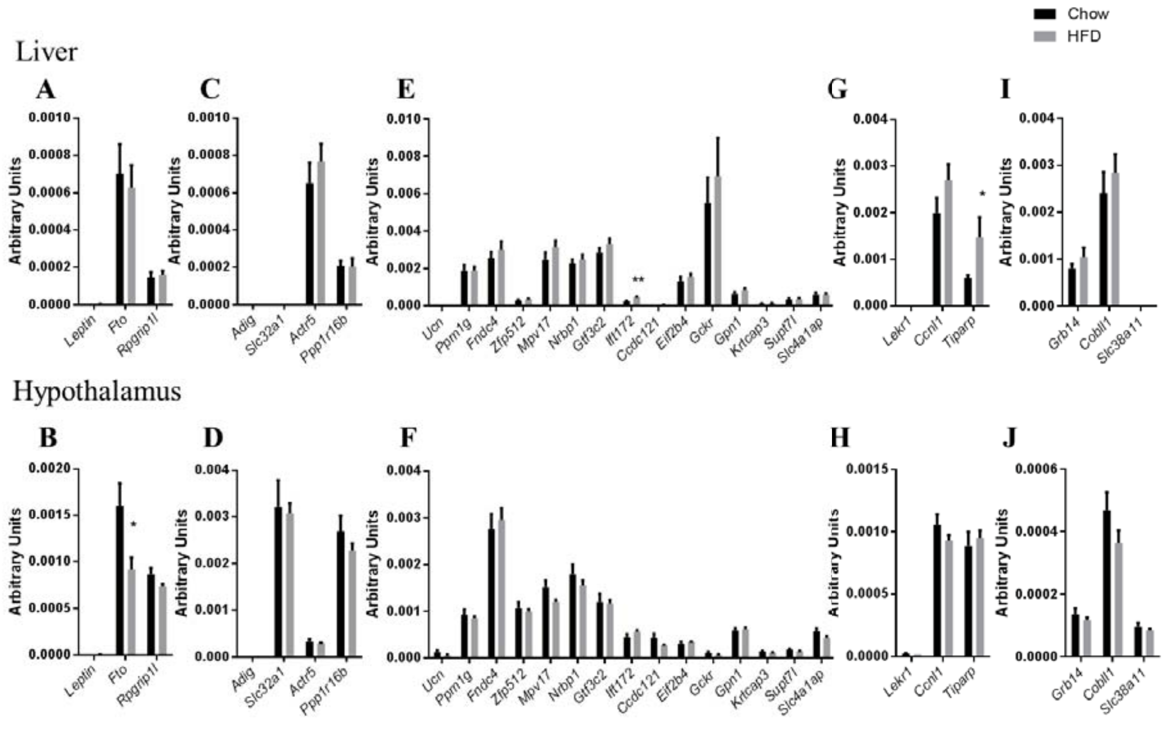

Supplementary Fig. 7. Expression of murine homologs of genes located within *Lep* (A, B), *Slc32a1* (C, D), *Gckr* (E, F), *Ccnl1* (G, H), and *Cobll1* loci (I, J) in liver and hypothalamus from 4 month old mice fed chow (black bars) or high fat diet (HFD; gray bars). qPCR transcripts were normalized using *ActB*, *36B4*, *Gapdh* and *Ppia* as housekeeping genes. N=5 mice per group. T-test. *p<0.05, **p<0.01.

Supplementary Tables

Supplementary Table 1. Study design, number of individuals, and sample quality control in studies included in Stage 1 meta-analyses

Study		Study design	Ethnicity	Total sample size	Sample QC		Samples in analyses	Leptin measurement		Reference
Short name	Full name				Call rate	other exclusions		Sample type	Assay	
**BLSA**	Baltimore Longitudinal Study on Aging	Population-based	White European	848	≥ 98.5%	1) Sex misspecification<br>2) Duplicates<br>3) Missing leptin data	532	Fasting Plasma	ELISA (LINCO Research, St. Charles, MO)	1
**COLAUS**	Cohorte Lausannoise	Population-based	Caucasian	5,406	≥ 90%	1) Ethnic outliers<br>2) Related individuals and duplicates<br>3) Missing leptin, BMI and fat mass data	4,926	Fasting serum	Sandwich enzyme-linked immunosorbent assay (Pathway diagnostics corporation, Los Angeles, CA, USA)	2
**ERF**	Erasmus Rucphen Family Study	Family-based	White European	3,000	≥ 95%	1) Missing DNA<br>2) Gender mismatch with typed X-linked markers<br>3) Excess autosomal heterozygosity<br>4) Ethnic outliers using IBS (95%)<br>5) Missing leptin measurements	1,957	Fasting plasma	Custom made duplex assay (cat.#: N45ZA-1) of Meso Scale Discovery (MSD).	3,4
**FamHS**	Family Heart Study	Family Based	White European	3,880	≥ 98%	1) Ethnic outliers<br>2) Related individuals and duplicates<br>3) Missing leptin data	1,505	Plasma	Human Leptin RIA Kit (LINCO Research)	5,6
**Fenland**	Fenland Study	Population-based	White European	1,500	≥ 95%	1) Heterozygosity <27.3% or >28.8%;<br>2) Duplicate check;<br>3) Relatedness check<br>4) Missing leptin data	1,397	Fasting serum	In-house two-site microtitre plate-based DELFIA assay. Antibodies & Standards from R&D Systems (R&D Systems Europe, Abingdon UK).	7,8

Study<br>Short name	Full name	Study design	Ethnicity	Total sample size	Call rate	Sample QC<br>other exclusions	Samples in analyses	Leptin measurement<br>Sample type	Assay	References
**FINRISK97**	The Example Study	Population-based	White European: Finnish	583	≥ 95%	1) Heterozygosity, gender check and relatedness checks have been performed and any discrepancies have been removed.<br>2) Missing phenotype	520	Semi-fasting serum	ELISA (R&D)	9
**GEMS**	Genetic Etiology of Metabolic Syndrome	Case-control for dyslipidemia	White European	1,789	≥ 90%	1) Gender inconsistency with genetic data from X-lined markers<br>2) Inconsistent genotypes when compared with control markers<br>3) Duplicates and first and second degree relatives<br>4) Missing phenotype or genotype	1,623	Fasting serum	ELISA (American Lab Products Company, Windham, NH)	10
**GOOD**	Gothenburg Osteoporosis and Obesity Determinants study	Population-based	White European	941	≥ 97.5%	1) Excessive heterozygosity<br>2) Ethnic outliers<br>3) Related individuals and duplicates<br>4) Sex mismatch<br>5) Missing leptin data	938	Non-fasting serum	Commercially available kit (Diagnostic Systems Laboratories Inc., Webster, TX; interassay CV 5.3%)	11
**H2000 CASES**	Health 2000 cases	Case-control	White European: Finnish	1,022	≥ 95%	1) Heterozygosity, gender check and relatedness checks have been performed and any discrepancies have been removed.<br>2) Missing phenotype.	991	Serum	ELISA (R&D)	12
**H2000 CTRLS**	Health 2000 controls	Case-control	White European: Finnish	1,081	≥ 95%	1) Heterozygosity, gender check and relatedness checks have been performed and any discrepansies have been removed.<br>2) Missing phenotype.	1032	Serum	ELISA (R&D)	12
**HBCS**	Helsinki Birth Cohort Study	Birth-cohort	White European: Finnish	1,872	≥ 95%	1) Heterozygosity, gender check and relatedness checks have been performed and any discrepansies have been removed.<br>2) Missing phenotype.	1605	Fasting serum	Immunoassay (Millipore, Luminex platform, 2008 Millipore Corporation, Billerica, MA, U.S.A.)	13

Study		Study design	Ethnicity	Total sample size	Sample QC		Samples in analyses	Leptin measurement		References
Short name	Full name				Call rate	other exclusions		Sample type	Assay	
**NHS**	Nurses' Health Study	Prospective nested case-control studies of type 2 diabetes and breast cancer (CGEM)	White European	5,573	≥95%	1) Missing call rate ≥2%<br>2) Presence of gross chromosomal anomalies<br>3) Ethnic outliers<br>4) Related individuals and duplicates<br>5) Missing leptin data	2,129	Plasma	Diabetes case-control study: Radioimmunoassay (EMD Millipore, Billerica, MA)<br>Breast cancer case-control study (CGEM): enzyme-linked immunosorbent assay (Diagnostic Systems, Webster, TX)	18,19,20
**QFS**	Quebec Family Study	Population-based	White, French Canadian	951	≥ 95%	1) Missing leptin data	583	Fasting serum	Radioimmunoassay (Linco, St. Charles, MO)	21
**RISC**	Relationship between Insulin Sensitivity and Cardiovascular Disease	Population-based	White European	1,566	≥ 95%	1) Non-European descent<br>2) Sex mismatches	1,031	Fasting serum	Two-step time-resolved fluorometric assays of the 1235 AutoDELFIA (PerkinElmer Lifesciences, Boston, MA) automatic immunoassay system	22,23
**Rotterdam study**	Rotterdam study	Population-based	White European	3,932	≥ 97.5%	1) gender mismatch with typed X-linked markers;<br>2) excess autosomal heterozygosity (F<-0.055);<br>3) duplicates and/or 1st degree relatives using IBD PiHAT >40% from PLINK;<br>4) ethnic outliers IBS distances > 4SD mean HaMAP CEU cluster from PLINK;<br>5) Missing leptin data.	955	Fasting serum	Radioimmunoassay	24,25,26
**TwinsUK**	TwinsUK	Twin cohort	White European	5,654	≥ 98%	1) Heterozygosity >2sd from the sample mean<br>2) Ethnic outliers<br>3) Missing leptin data	2,690	Fasting serum	Radioimmunoassay (Linco Research, St Louis, MO)	27
**YFS**	The Cardiovascular Risk in Young Finns Study	Population-based	White European: Finnish	2,556	≥ 95%	1) Heterozygosity, gender check and relatedness checks have been performed and any discrepansies have been removed.<br>2) Missing phenotype.	2,085	Serum	Radioimmunoassay (Human Leptin RIA kit, Linco Research, Inc., MO, USA)	28

Supplementary Table 2. Meta-analysis results in men and women combined for the leptin-associated loci that were taken forward for Stage 2

SNP	Nearest gene	CHR	Pos	Effect allele	Other allele	EAF	Leptin unadjusted for BMI								Leptin adjusted for BMI							
							Stage 1		Stage 2		Stage 1+2				Stage 1		Stage 2		Stage 1+2			
							P	N	P	N	Beta	SE	P	N	P	N	P	N	Beta	SE	P	N
rs780093	*GCKR*	2	27596107	C	T	0.61	1.8E-07	32147	3.6E-04	19979	0.032	0.005	2.3E-10	52126	6.3E-07	31802	1.4E-04	19648	0.024	0.004	3.8E-10	51450
rs6738627	*COBLL1*	2	165252696	A	G	0.37	8.3E-07	25573	8.6E-02	19573	0.027	0.006	1.4E-06	45146	4.6E-06	25229	4.5E-02	19242	0.020	0.004	1.9E-06	44471
rs900400	*CCNL1*	3	158281469	T	C	0.60	7.3E-07	32128	1.7E-03	19685	0.030	0.005	5.6E-09	51813	9.8E-07	31785	1.7E-02	19354	0.021	0.004	1.2E-07	51139
rs1738457	*MDGA1*	6	37661173	C	T	0.45	9.8E-03	29467	9.1E-01	17409	0.011	0.005	3.6E-02	46876	2.7E-02	29252	3.2E-01	17078	0.005	0.004	2.6E-01	46330
rs10487505	*LEP*	7	127647399	G	C	0.50	4.0E-06	29470	2.0E-01	17110	0.023	0.005	9.0E-06	46580	2.7E-11	29255	5.2E-03	16781	0.029	0.004	2.0E-12	46036
rs8043757	*FTO*	16	52370951	T	A	0.40	8.8E-08	32120	2.7E-03	19919	0.030	0.005	1.1E-09	52039	6.1E-01	31776	7.5E-01	19588	0.001	0.004	8.4E-01	51364
rs6071166	*SLC32A1*	20	36766426	C	A	0.37	2.0E-07	29471	1.6E-01	17007	0.027	0.006	6.6E-07	46478	2.9E-08	29256	3.9E-02	16678	0.024	0.004	1.8E-08	45934

Leptin (µg/ml) was logarithmically transformed. The study-specific analyses were performed with linear regression models while accounting for sex, age and age ^{2}. Meta-analyses of the study-specific results were performed using fixed effects meta-analysis. Beta refers to the change in logarithmically transformed leptin per each copy of the effect allele. The near *MDGA1* locus was taken forward from the stage 1 meta-analyses for women (**Supplementary Table 4**).

Beta, change in logarithmically transformed leptin per each copy of the effect allele; EAF, effect allele frequency

Supplementary Table 3. Meta-analysis results in men for the leptin-associated loci that were taken forward for Stage 2.

SNP	Nearest gene	CHR	Pos	Effect allele	Other allele	EAF	Leptin unadjusted for BMI								Leptin adjusted for BMI							
							Stage 1		Stage 2		Stage 1+2				Stage 1		Stage 2		Stage 1+2			
							P	N	P	N	Beta	SE	P	N	P	N	P	N	Beta	SE	P	N
rs780093	*GCKR*	2	27596107	C	T	0.61	1.0E-02	13463	3.8E-03	9848	0.029	0.0076	1.3E-04	23311	5.0E-02	13177	2.8E-04	9693	0.024	0.0061	1.1E-04	22870
rs6738627	*COBLL1*	2	165252696	A	G	0.37	1.0E-03	10428	3.6E-01	9635	0.025	0.0085	3.4E-03	20063	2.3E-03	10142	1.8E-01	9480	0.021	0.0068	2.3E-03	19622
rs900400	*CCNL1*	3	158281469	T	C	0.60	2.4E-02	13463	3.6E-03	9705	0.029	0.0078	2.7E-04	23168	1.6E-01	13177	9.6E-03	9550	0.018	0.0063	4.9E-03	22727
rs1738457	*MDGA1*	6	37661173	C	T	0.45	8.1E-02	13460	2.6E-01	8511	-0.005	0.0079	5.5E-01	21971	4.3E-01	13174	8.6E-01	8354	-0.003	0.0064	6.3E-01	21528
rs10487505	*LEP*	7	127647399	G	C	0.50	3.9E-02	13463	9.9E-01	8384	0.012	0.0077	1.1E-01	21847	2.4E-04	13177	1.2E-01	8229	0.024	0.0062	1.5E-04	21406
rs8043757	*FTO*	16	52370951	T	A	0.40	1.2E-06	13463	8.2E-03	9825	0.041	0.0076	8.6E-08	23288	3.9E-02	13177	2.1E-01	9670	0.014	0.0061	1.8E-02	22847
rs6071166	*SLC32A1*	20	36766426	C	A	0.37	6.9E-04	13462	7.6E-01	8311	0.022	0.0081	5.4E-03	21773	1.1E-03	13176	2.1E-01	8156	0.021	0.0065	1.1E-03	21332

Leptin (µg/ml) was logarithmically transformed. The study-specific analyses were performed with linear regression models while accounting for sex, age and age ^{2}. Meta-analyses of the study-specific results were performed using fixed effects meta-analysis. Beta refers to the change in logarithmically transformed leptin per each copy of the effect allele.

Beta, change in logarithmically transformed leptin per each copy of the effect allele; EAF, effect allele frequency

Supplementary Table 4. Meta-analysis results in women for the leptin-associated loci that were taken forward for Stage 2.

SNP	Nearest gene	CHR	Pos	Effect allele	Other allele	EAF	Leptin unadjusted for BMI								Leptin adjusted for BMI							
							Stage 1		Stage 2		Stage 1+2				Stage 1		Stage 2		Stage 1+2			
							P	N	P	N	Beta	SE	P	N	P	N	P	N	Beta	SE	P	N
rs780093	*GCKR*	2	27596107	C	T	0.61	6.0E-06	18684	3.1E-02	10131	0.033	0.0067	7.9E-07	28815	2.8E-06	18625	7.3E-02	9955	0.025	0.0050	1.0E-06	28580
rs6738627	*COBLL1*	2	165252696	A	G	0.37	1.1E-04	15145	1.1E-01	9938	0.029	0.0073	6.1E-05	25083	4.6E-04	15087	1.3E-01	9762	0.020	0.0055	2.0E-04	24849
rs900400	*CCNL1*	3	158281469	T	C	0.60	2.9E-06	18665	1.4E-01	9980	0.032	0.0068	3.3E-06	28645	8.1E-07	18608	4.4E-01	9804	0.023	0.0051	9.4E-06	28412
rs1738457	*MDGA1*	6	37661173	C	T	0.45	7.9E-07	16007	3.2E-01	8898	0.025	0.0072	6.4E-04	24905	4.0E-04	16078	1.2E-01	8724	0.011	0.0055	5.1E-02	24802
rs10487505	*LEP*	7	127647399	G	C	0.50	1.8E-05	16007	5.8E-02	8726	0.033	0.0071	4.1E-06	24733	1.4E-08	16078	1.5E-02	8552	0.033	0.0054	1.2E-09	24630
rs8043757	*FTO*	16	52370951	T	A	0.40	1.6E-03	18657	1.0E-01	10094	0.023	0.0066	4.5E-04	28751	4.8E-01	18599	1.1E-01	9918	-0.008	0.0050	1.3E-01	28517
rs6071166	*SLC32A1*	20	36766426	C	A	0.37	1.0E-04	16008	9.6E-02	8696	0.031	0.0074	3.7E-05	24704	1.3E-05	16079	1.1E-01	8522	0.025	0.0057	7.5E-06	24601

Leptin (µg/ml) was logarithmically transformed. The study-specific analyses were performed with linear regression models while accounting for sex, age and age ^{2}. Meta-analyses of the study-specific results were performed using fixed effects meta-analysis. Beta refers to the change in logarithmically transformed leptin per each copy of the effect allele.

Beta, change in logarithmically transformed leptin per each copy of the effect allele; EAF, effect allele frequency

Supplementary Table 5. Study design, number of individuals and sample quality control in studies included in Stage 2 meta-analyses.

Study		Study design	Ethnicity	Total N	Sample QC		N in analyses	Leptin measurement		Reference
Short name	Full name				Call rate	other exclusions		Sample type	Assay	
**BWHHS**	British Women's Heart & Health study	Prospective Cohort study	White European	4,286	>90%	Gender mismatch	500	Fasting serum	In-house (University of Glasgow) radioimmunoassay validated against the commercially available Linco assay.	29
**Ely**	MRC Ely Study	Population-based	White European	1,625	≥ 95%	1) missing phenotype data<br>2) gender check;<br>3) duplicates check;	1,595	Fasting serum	Two-step time-resolved fluorometric assays of the 1235 AutoDELFIA (PerkinElmer Lifesciences, Boston, MA) automatic immunoassay system	30
**GARP**	The Genetics osteoARthritis and Progression study	Affected sibling pairs	White European	250	> 98%	1) MAF > 5%<br>2) missing leptin data<br>3) imputation quality score > 95	240	Morning Serum	Bio-Plex Pro Human Diabetes kit (Bio-Rad, USA), the Bio-plex array reader and Bio-plex software	31,32
**GLACIER**	Gene x Lifestyle interactions And Complex traits Involved in Elevated disease Risk	Population-based	White European	17698	≥ 95%	1) missing relevant phenotype data<br>2) missing all five SNPs for replication<br>3) self-reported diabetes<br>4) fasting and 2-hr glucose levels indicative of type 2 diabetes	992	Fasting serum	Radioimmunoassay (Linco, St. Charles, MO, USA)	33,34
**GOYA Male**	Genetics of Extremely Overweight Young Adults	Population-based	White European	1,632	≥ 95%	1) heterozygosity <30.2% or >35%<br>2) discordance in SNP pairs when compared with previous project<br>3) ethnic outliers<br>4) related individuals and duplicates	467	Fasting serum	Fluro-immunometric assay with a Leptin RIA kit (Alt Diagnostica, Marburg, Germany)	35

Study Short name	Full name	Study design	Ethnicity	Total sample size	Call rate+F3	Sample QC other exclusions	Samples in analyses	Leptin measurement Sample type	Assay	References
**LURIC**	Ludwigshafen Risk and Cardiovascular Health Study	Case-control	White European	3,316	≥ 95%	1) related individuals and duplicates<br>2) missing leptin data<br>3) missing genotyping<br>4) sex ambiguity<br>5) cardiovascular disease	599	Fasting plasma	Human leptin RIA kit/LKB Valle 1277, γ Master, Uppsala, Sweden	36
**MESA**	Multi-Ethnic Study of Atherosclerosis	Population-based	Non-Hispanic White	6,814	95%	1) Ethnic outliers<br>2) Duplicates<br>3) Gender mismatch<br>4) Related individuals<br>5) Missing leptin data	760	Fasting Serum	Bio-Rad Luminex flow cytometry (Millepore, Billerica, MA).	37,38
**MrOS Sweden**	The Osteoporotic Fractures in Men Sweden	Population-based	White European	962	≥ 97%	1) exclusion based on IBD clustering<br>2) ethnic outliers<br>3) related individuals and duplicates<br>4) missing leptin data	919	Fasting serum	Commercially available kit (Diagnostic Systems Laboratories Inc., Webster, TX; interassay CV 5.3%)	39
**PIVUS**	Prospective Investigation of the Vasculature in Uppsala Seniors	Population-based	White European	1,016	≥ 99%	1) heterozygosity<br>2) ethnic outliers<br>3) duplicates<br>4) gender discordance	948	Fasting plasma	Double-antibody radioimmunoassays (RIA) (Linco Research, St. Louis, MO, USA).	40
**PROSPER**	The PROspective Study of Pravastatin in Elderly at risk for vascular disease	Population-based	White European	5,796	>97.5	1) Excess of heterozygosity<br>2) Ethnic outliers,<br>3) Familial relationships<br>4) Gender mismatch<br>5) Missing leptin data	5,148	Fasting plasma	In-house RIA validated thoroughly against the commercially available Linco Research Co. (St. Charles, MO) assay	41
**WGHS**	Women's Genome Health Study	The WGHS is population-based. The WGHS sub-population with leptin measures was a nested case/cohort design subsample for incident type 2 diabetes.	White European	23,294	≥ 98%	1) age<45<br>2) prior CVD<br>3) cancer<br>4) DM<br>5) missing BMI	808	Fasting serum	Ultra-sensitive ELISA assay, an enzymatically amplified "two-step" sandwich-type immunoassay (R&D Systems, Minneapolis, MN).	

Supplementary Table 6. Meta-analysis results for the leptin-associated loci when adjusting for BMI or body fat percentage.

SNP	Nearest gene	CHR	Pos	Effect allele	Other allele	EAF	Leptin adj. BMI				Leptin adj. body fat percentage			
							Beta	SE	P	N	Beta	SE	P	N
rs780093	*GCKR*	2	27596107	C	T	0.60	0.017	0.0057	3.8E-03	21765	0.026	0.0059	6.9E-06	18980
rs6738627	*COBLL1*	2	165252696	A	G	0.37	0.020	0.0071	5.2E-03	15192	0.014	0.0073	5.2E-02	12532
rs900400	*CCNL1*	3	158281469	T	C	0.61	0.021	0.0059	3.3E-04	21748	0.024	0.0060	9.2E-05	18966
rs10487505	*LEP*	7	127647399	G	C	0.49	0.034	0.0062	3.6E-08	19220	0.041	0.0063	9.4E-11	16946
rs8043757	*FTO*	16	52370951	T	A	0.40	0.003	0.0058	5.9E-01	21740	0.000	0.0059	9.8E-01	18960
rs6071166	*SLC32A1*	20	36766426	C	A	0.38	0.035	0.0065	6.0E-08	19219	0.021	0.0067	2.2E-03	16945

Leptin (µg/ml) was logarithmically transformed. Only the studies that had data available of both BMI and body fat percentage were included The study-specific analyses were performed with linear regression models while accounting for sex, age, age ^{2}, and BMI or body fat percentage. Meta-analyses of the study-specific results were performed using fixed effects meta-analysis. Beta refers to the change in logarithmically transformed leptin per each copy of the effect allele.

Beta, change in logarithmically transformed leptin per each copy of the effect allele; EAF, effect allele frequency

Supplementary Table 7. Association of leptin-associated loci with other traits in meta-analyses of GWAS.

	rs780093 (*GCKR*) C allele				rs6738627 (*COBLL1*) A allele				rs900400 (*CCNL1*) T allele				rs10487505 (*LEP*) G allele				rs6071166 (*SLC32A1*) C allele				
**Trait**	**β**	**SE**	***N***	***P* value**	**β**	**SE**	***N***	***P* value**	**β**	**SE**	***N***	***P* value**	**β**	**SE**	***N***	***P* value**	**β**	**SE**	***N***	***P* value**	**Ref.**
Body mass index[1]	0.012	0.003	321,115	2.28E-04	0.010	0.003	267,719	3.78E-03	0.005	0.004	233,872	1.77E-01	-0.009	0.004	221,677	2.53E-02	0.006	0.004	233,392	1.27E-01	42
Body fat percentage[2]	0.005	0.005	89,257	3.24E-01	0.030	0.005	76,338	1.84E-08	0.004	0.006	64,032	5.38E-01	-0.002	0.006	62,795	7.09E-01	0.011	0.006	64,035	8.52E-02	45
BMI-adjusted waist-hip ratio[1]	-0.009	0.004	209,080	4.90E-03	-0.020	0.004	174,672	2.20E-08	0.026	0.004	141,215	5.90E-09	-0.002	0.004	130,812	6.30E-01	0.003	0.005	142,286	4.10E-01	43
Height[1]	0.020	0.003	253,171	9.60E-12	0.001	0.003	197,773	7.20E-01	-0.003	0.003	252,995	4.20E-01	0.004	0.003	240,862	1.70E-01	-0.003	0.003	250,810	3.50E-01	44
Fasting glucose[3]	0.027	0.004	~58,074	2.90E-10	-0.008	0.004	~58,074	2.96E-02	-0.004	0.004	~58,074	3.71E-01	0.001	0.004	~58,074	7.94E-01	0.004	0.004	~58,074	3.40E-01	46
Fasting insulin[3]	0.014	0.004	~51,750	1.98E-04	-0.011	0.004	~51,750	2.50E-03	-0.005	0.004	~51,750	2.06E-01	-0.003	0.004	~51,750	4.53E-01	0.003	0.004	~51,750	4.62E-01	46
Total cholesterol[4]	-	NA	100,176	5.46E-25	-	NA	100,176	1.53E-03	-	NA	100,184	2.20E-01	-	NA	100,184	9.08E-01	+	NA	98,656	6.80E-01	47
High-density lipoprotein cholesterol[4]	+	NA	99,892	4.50E-02	+	NA	99,892	2.47E-06	-	NA	99,900	1.23E-03	-	NA	99,900	6.47E-01	-	NA	98,409	5.28E-01	47
Low-density lipoprotein cholesterol[4]	-	NA	95,446	2.41E-04	-	NA	95,446	3.61E-04	-	NA	95,454	5.24E-01	-	NA	95,454	6.87E-01	+	NA	93,999	5.27E-01	47
Triglycerides[4]	-	NA	96,590	5.68E-124	-	NA	96,590	4.43E-07	+	NA	96,598	3.05E-02	+	NA	96,598	5.07E-01	+	NA	95,070	6.20E-01	47
Systolic blood pressure[5]	NA	NA	~69,395	3.48E-01	NA	NA	~69,395	2.02E-01	NA	NA	~69,395	3.51E-01	NA	NA	~69,395	1.29E-01	NA	NA	~69,395	3.30E-01	48
Diastolic blood pressure[5]	NA	NA	~69,395	5.43E-01	NA	NA	~69,395	5.96E-01	NA	NA	~69,395	2.72E-01	NA	NA	~69,395	2.25E-01	NA	NA	~69,395	4.61E-01	48
Birth weight[6]	-0.004	0.008	~28,636	6.50E-01	-0.002	0.010	~28,636	8.30E-01	0.073	0.009	~28,636	1.20E-17	-0.019	0.008	~28,636	2.40E-02	-0.005	0.009	~28,636	5.60E-01	49
Adiponectin[7]	0.016	0.004	29,336	4.50E-04	0.015	0.005	20,653	7.94E-03	-0.015	0.005	29,340	1.84E-03	0.003	0.004	29,084	5.74E-01	0.000	0.005	29,165	9.34E-01	50
Age at menarche[8]	-0.017	NA	~132,989	4.20E-03	-0.003	NA	~132,989	7.10E-01	0.037	NA	~132,989	3.80E-10	0.003	NA	~132,989	5.90E-01	0.004	NA	~132,989	4.90E-01	51
C-reactive protein[9]	-0.087	0.006	~66,185	1.08E-41	-0.002	0.009	~66,185	8.40E-01	0.004	0.007	~66,185	5.51E-01	0.005	0.006	~66,185	3.99E-01	-0.001	0.007	~66,185	9.35E-01	52
Carbohydrate intake (% of energy)[10]	-0.034	0.058	~37,537	5.70E-01	-0.012	0.063	~37,537	8.56E-01	0.085	0.061	~37,537	1.69E-01	0.155	0.057	~37,537	7.73E-03	-0.022	0.060	~37,537	7.18E-01	53
Fat intake (% of energy)[10]	0.078	0.047	~37,537	1.03E-01	0.132	0.050	~37,537	8.88E-03	0.012	0.049	~37,537	8.11E-01	-0.077	0.046	~37,537	9.99E-02	-0.020	0.049	~37,537	6.90E-01	53
Protein intake (% of energy)[10]	-0.053	0.025	~37,537	3.86E-02	-0.030	0.027	~37,537	2.84E-01	-0.009	0.026	~37,537	7.54E-01	-0.040	0.025	~37,537	1.11E-01	0.053	0.026	~37,537	4.93E-02	53
	**OR**	**95% CI**	***N***	***P* value**	**OR**	**95% CI**	***N***	***P* value**	**OR**	**95% CI**	***N***	***P* value**	**OR**	**95% CI**	***N***	***P* value**	**OR**	**95% CI**	***N***	***P* value**	**Ref.**
Risk of early-onset obesity[6]	0.97	0.92-1.02	~13,848	1.91E-01	1.04	0.99-1.11	~13,848	1.27E-01	1.00	0.95-1.06	~13,848	9.04E-01	0.95	0.90-1.00	~13,848	4.02E-02	1.02	0.97-1.08	~13,848	4.91E-01	54
Risk of type 2 diabetes[11]	1.06	1.03-1.08	79,616	3.70E-06	0.95	0.92-0.97	80,652	1.43E-05	0.97	0.95-1.00	80,625	3.90E-02	1.00	0.96-1.04	63,390	9.90E-01	1.01	0.97-1.05	63,390	6.60E-01	55

For each locus, the effect allele is the leptin-increasing allele

[1] Association results for body mass index, BMI-adjusted waist-hip ratio, and height were obtained from the Genetic Investigation of Anthropometric Traits (GIANT) Consortium

[2] Association results for body fat percentage were obtained from the Body Composition Genetics Consortium (BCGC)

[3] Association results for fasting glucose and fasting insulin were obtained from the Meta-Analyses of Glucose and Insulin-Related Traits Consortium (MAGIC)

[4] Association results for HDL-, LDL-, and total cholesterol and triglycerides levels were obtained from the Global Lipids Genetics Consortium (GLGC)

[5] Association results for diastolic and systolic blood pressure were obtained from the International Consortium for Blood Pressure (ICBP)

[6] Association results for birth weight and risk of early-onset obesity were obtained from the Early Growth Genetics (EGG) consortium

[7] Association results for adiponectin levels were obtained from the ADIPOGen Consortium

[8] Association results for age at menarche were obtained from the ReproGen consortium

[9] Association results for C-reactive protein levels were obtained from the Cohorts for Heart and Aging Research in Genomic Epidemiology (CHARGE) consortium

[10] Association results for macronutrient intakes were obtained from the CHARGE consortium

[11] Association results for type 2 diabetes-risk were obtained from the Diabetes Genetics Replication and Meta-analysis (DIAGRAM) Consortium

Supplementary Table 8. Data used in *cis*-eQTL analyses.

Study	Tissues (*n*)	Genotype platform	Gene expression platform	Reference
Bariatric case series	Liver (n=567), Omental fat (n=742), Subcutaneous fat (n=610)	Illumina 650K	Custom Agilent 44,000 feature microarray composed of 39,280 oligonucleotide probes	56
Cortical brain study	Brain (n=193)	Affymetrix Human Mapping 500K	Illumina Human Mapping Refseq-8 Expression Beadchip	57
MolOBB	Abdominal adipose (n=54), Gluteal fat (n=65), Whole blood (n=68)	Illumina 317K	Affymetrix Human Genome U133 Plus 2.0 gene-expression microarrays (hgu133plus2 arrays) with 17,726 non-overlapping probes	58
MuTHER	Lymphoblastoid cell line (n=778), Skin (n=667), Subcutaneous fat (n=776)	Illumina HumanHap300, HumanHap610Q, 1M-Duo, and 1.2M Duo 1M	Illumina Human HT-12 V3 Beadchip	59

Supplementary Table 9. Association of the leptin-associated SNPs with *cis*-gene expression (*cis*-eQTLs)

SNP	Chr	Leptin-increasing allele	Tissue	Gene	β for leptin SNP	P for leptin SNP	P_{adj}[a] for leptin SNP	Peak SNP[b]	r^2 [c]	P for peak SNP	P_{adj}[d] for peak SNP	Ref.
rs780093	2	C	Liver	*IFT172*	0.041	7.51E-30	1.20E-01	rs1260342	0.41	4.31E-101	3.70E-41	56
			Liver	*KRTCAP3*	0.029	6.76E-06	3.70E-01	rs1260342	0.41	2.10E-17	2.65E-08	56
			Liver	*GCKR*	-0.023	4.44E-05	NA	NA	NA	NA	NA	56
			Omental	*IFT172*	0.089	6.49E-64	9.20E-01	rs1647276	0.45	7.63E-220	1.17E-69	56
			Omental	*KRTCAP3*	0.018	2.05E-05	1.08E-01	rs780110	0.45	1.22E-18	3.89E-09	56
			Subcutaneous	*IFT172*	0.084	2.70E-52	8.69E-01	rs1260342	0.43	1.01E-188	1.37E-61	56
rs6738627	2	A	Omental	*GRB14*	-0.053	5.31E-12	9.81E-01	rs6717858	0.84	3.42E-14	2.07E-01	56
			Subcutaneous	*GRB14*	-0.042	3.12E-05	9.52E-01	rs10184004	0.78	1.66E-06	2.68E-01	56
rs900400	3	T	Subcutaneous	*TIPARP*	-0.278	6.75E-58	NA	rs900400	1	6.75E-58	NA	59
			Subcutaneous	*TIPARP*	-0.099	7.51E-53	7.78E-01	rs10049090	0.94	5.90E-55	4.07E-01	56
rs6071166	20	C	Liver	*ACTR5*	0.032	2.36E-10	7.42E-03	rs6028156	0.08	7.63E-46	1.77E-35	56

[a]*P* value for the leptin SNP after adjusting for the peak SNP

[b]Most significant SNP associated with the gene transcript

[c]r^2 between the leptin SNP and the peak SNP

[d]*P* value for the peak SNP after adjusting for the leptin SNP

Supplementary Table 10. Summary of knockdown of candidate genes and effects on leptin transcript and protein secretion in perigonadal adipose tissue explants from high fat diet-fed mice.

	**Candidate gene mRNA (AU)**			***Lep* mRNA (AU)**			**Leptin protein (ng/ml)**		
**Candidate gene knocked down**	**Percent change (%)**	**SEM (%)**	**p-value**	**Percent change (%)**	**SEM (%)**	**p-value**	**Percent change (%)**	**SEM (%)**	**p-value**
Lep (n=5)				-92.3	1.3	<0.0001	-91.9	1.2	0.0004
Adig (n=12)	-97.8	0.3	<0.0001	-25.6	5.1	0.0004	-23.3	4.4	0.003
Ift177 (n=9)	-57.9	3.4	<0.0001	-21.9	8.6	0.02	-2.2	9	0.57
Mpv17 (n=12)	-46.2	2.4	<0.0001	5.7	4	0.18	9.7	4	0.17
Tiparp (n=9)	-40.2	2.9	<0.0001	3.3	9.2	0.72	4.3	6.7	0.84
Cobll1 (n=13)	-79.4	1.5	<0.0001	-9.3	7.4	0.23	-16.2	3.3	0.0003

Supplementary Table 11. Reported associations of leptin SNPs with complex traits in the NHGRI GWAS catalog.

Leptin SNP (LS)	LS position	r^2	Previously identified SNPs (PIS)	PIS position	PMID	Disease/trait	Previously reported gene(s)	LS effect allele	LS EAF	PIS effect allele	PIS EAF	OR/β	Direction of effect for leptin-increasing allele (+/-)	*P* value	Distance between LS and PIS
rs900400	158,281,469	0.97	rs1482853	156,798,473	23575227	Adiposity in newborns	*LEKR1, CCNL1*	T	0.6	C	0.620	.06	+	6E-21	302
rs900400	158,281,469	1.00	rs900400	156,798,775	23202124	Birth weight	*CCNL1*	T	0.6	T	0.61	.07	+	4E-38	0
rs900400	158,281,469	1.00	rs900400	156,798,775	20372150	Birth weight	*CCNL1, LEKR1*	T	0.6	T	NR	.09	+	2E-35	0
rs780093	27,596,107	0.97	rs780094	27,594,741	24068962	Calcium levels	*GCKR*	C	0.61	T	0.42	.02	-	1E-10	1366
rs780093	27,596,107	0.90	rs1260326	27,584,444	21943158	factors	*GCKR*	C	0.61	T	0.40	.08	-	2E-8	11663
rs780093	27,596,107	0.90	rs1260326	27,584,444	24097068	Cholesterol, total	*GCKR*	C	0.61	T	0.39	.05	-	3E-42	11663
rs780093	27,596,107	0.90	rs1260326	27,584,444	20686565	Cholesterol, total	*GCKR*	C	0.61	T	0.41	1.91	-	7E-27	11663
rs780093	27,596,107	0.90	rs1260326	27,584,444	20383146	Chronic kidney disease	*GCKR, IFT172, FNDC4*	C	0.61	T	0.41	.01	-	3E-14	11663
rs780093	27,596,107	0.90	rs1260326	27,584,444	21300955	C-reactive protein	*GCKR*	C	0.61	T	NR	.07	N/A	5E-40	11663
rs780093	27,596,107	0.97	rs780094	27,594,741	18439548	C-reactive protein	*GCKR*	C	0.61	A	NR	.14	N/A	7E-15	1366
rs780093	27,596,107	1.00	rs780093	27,596,107	21102463	Crohn's disease	*GCKR*	C	0.61	T	0.418	1.15	-	5E-11	0
rs780093	27,596,107	0.97	rs780094	27,594,741	20081858	Fasting glucose	*GCKR*	C	0.61	C	0.62	NR	+	6E-38	1366
rs780093	27,596,107	0.97	rs780094	27,594,741	20081858	Fasting insulin	*GCKR*	C	0.61	C	0.62	NR	+	4E-20	1366
rs780093	27,596,107	0.97	rs780094	27,594,741	20081858	HOMA-IR	*GCKR*	C	0.61	C	0.62	NR	+	3E-24	1366
rs780093	27,596,107	0.97	rs780094	27,594,741	22581228	Fasting glucose-related traits (interaction with BMI)	*GCKR*	C	0.61	NR	NR	NR	N/A	4E-24	1366
rs780093	27,596,107	0.97	rs780094	27,594,741	22581228	Fasting insulin-related traits (interaction with BMI)	*GCKR*	C	0.61	NR	NR	NR	N/A	3E-10	1366
rs780093	27,596,107	0.90	rs1260326	27,584,444	23903356	Fasting plasma glucose in pregnancy	*GCKR*	C	0.61	T	0.409	.00	-	6E-13	11663
rs780093	27,596,107	0.90	rs1260326	27,584,444	23903356	Fasting C-peptide in pregnancy	*GCKR*	C	0.61	T	0.409	.01	-	6E-11	11663
rs780093	27,596,107	0.90	rs1260326	27,584,444	20139978	Hematological and biochemical traits	*GCKR*	C	0.61	C	0.44	.09	-	4E-9	11663

Leptin SNP (LS)	LS position	r^2	Previously identified SNPs (PIS)	PIS position	PMID	Disease/trait	Previously reported gene(s)	LS effect allele	LS EAF	PIS effect allele	PIS EAF	OR/β	Direction of effect for leptin-increasing allele (+/-)	*P* value	Distance between LS and PIS
rs780093	27,596,107	0.90	rs1260326	27,584,444	23505323	Hypertriglyceridemia	*ZNF512,PPM1G, NRBP1,KRTCAP3, IFT172,GCKR,FN DC4,C2orf16*	C	0.61	NR	0.26	1.41	-	2E-13	11663
rs780093	27,596,107	0.90	rs1260326	27,584,444	20657596	Hypertriglyceridemia	*GCKR*	C	0.61	T	0.41	1.75	-	7E-9	11663
rs780093	27,596,107	0.97	rs780094	27,594,741	18179892	LDL cholesterol	*GCKR*	C	0.61	T	0.39	NR	-	5E-7	1366
rs780093	27,596,107	0.90	rs1260326	27,584,444	19936222	Lipid metabolism phenotypes (triglycerides by assay, fasting)	*GCKR*	C	0.61	NR	NR	.08	N/A	4E-32	11663
rs780093	27,596,107	0.90	rs1260326	27,584,444	19936222	Lipid metabolism phenotypes (triglycerides by assay)	*GCKR*	C	0.61	NR	NR	.07	N/A	1E-37	11663
rs780093	27,596,107	0.90	rs1260326	27,584,444	19936222	Lipid metabolism phenotypes (triglycerides by NMR, fasting)	*GCKR*	C	0.61	NR	NR	.06	N/A	3E-29	11663
rs780093	27,596,107	0.90	rs1260326	27,584,444	19936222	Lipid metabolism phenotypes (triglycerides by NMR)	*GCKR*	C	0.61	NR	NR	.05	N/A	3E-35	11663
rs780093	27,596,107	0.90	rs1260326	27,584,444	19936222	Lipid metabolism phenotypes (VLDL large)	*GCKR*	C	0.61	NR	NR	.36	N/A	4E-24	11663
rs780093	27,596,107	0.90	rs1260326	27,584,444	19936222	Lipid metabolism phenotypes (VLDL large, fasting)	*GCKR*	C	0.61	NR	NR	.34	N/A	3E-28	11663
rs780093	27,596,107	0.90	rs1260326	27,584,444	22001757	Liver enzyme levels (gamma-glutamyl transferase)	*C2orf16, GCKR*	C	0.61	T	0.38	3.20	-	4E-13	11663
rs780093	27,596,107	0.97	rs780094	27,594,741	22399527	Metabolic syndrome	*GCKR*	C	0.61	A	0.36	.13	-	6E-20	1366
rs780093	27,596,107	0.90	rs1260326	27,584,444	19060910	Metabolic traits	*GCKR*	C	0.61	A	0.35	.09	-	4E-10	11663
rs780093	27,596,107	0.97	rs780094	27,594,741	21886157	Metabolic traits	*GCKR*	C	0.61	T	0.399	.10	-	6E-53	1366
rs780093	27,596,107	0.90	rs1260326	27,584,444	22916037	Metabolite levels	*GCKR*	C	0.61	NR	0.36	NR	-	1E-12	11663
rs780093	27,596,107	0.90	rs1260326	27,584,444	22286219	Metabolite levels	*GCKR*	C	0.61	NR	NR	.15	N/A	3E-18	11663
rs780093	27,596,107	0.90	rs1260326	27,584,444	22558069	Non-albumin protein levels	*GCKR*	C	0.61	C	0.445	.08	-	3E-9	11663
rs780093	27,596,107	1.00	rs780093	27,596,107	23362303	Palmitoleic acid (16:1n-7) plasma levels	*GCKR, C2orf16, ZNF512, XAB1*	C	0.61	T	0.41	.02	-	1E-9	0
rs780093	27,596,107	0.97	rs780094	27,594,741	21829377	Phospholipid levels (plasma)	*GCKR*	C	0.61	T	NR	.02	-	9E-9	1366
rs780093	27,596,107	0.90	rs1260326	27,584,444	22139419	Platelet counts	*GCKR*	C	0.61	T	NR	2.33	-	9E-10	11663
rs780093	27,596,107	0.90	rs1260326	27,584,444	23022100	Serum albumin level	*GCKR-FNDC4*	C	0.61	T	0.41	.01	-	4E-19	11663

Leptin SNP (LS)	LS position	r^2	Previously identified SNPs (PIS)	PIS position	PMID	Disease/trait	Previously reported gene(s)	LS effect allele	LS EAF	PIS effect allele	PIS EAF	OR/β	Direction of effect for leptin-increasing allele (+/-)	*P* value	Distance between LS and PIS
rs780093	27,596,107	1.00	rs780093	27,596,107	22829776	Sex hormone-binding globulin levels	*GCKR*	C	0.61	T	0.40	.03	-	2E-16	0
rs780093	27,596,107	1.00	rs780093	27,596,107	22829776	Sex hormone-binding globulin levels	*GCKR*	C	0.61	T	0.40	.04	-	9E-11	0
rs780093	27,596,107	0.74	rs4665972	27,451,601	23726366	Triglycerides	*GCKR*	C	0.61	T	0.123	.07	-	1E-8	144506
rs780093	27,596,107	0.90	rs1260326	27,584,444	24097068	Triglycerides	*GCKR*	C	0.61	T	0.39	.12	-	2E-239	11663
rs780093	27,596,107	0.90	rs1260326	27,584,444	20686565	Triglycerides	*GCKR*	C	0.61	T	0.41	8.76	-	6E-133	11663
rs780093	27,596,107	0.90	rs1260326	27,584,444	20139978	Triglycerides	*GCKR*	C	0.61	C	0.45	.10	-	1E-11	11663
rs780093	27,596,107	0.90	rs1260326	27,584,444	19060906	Triglycerides	*GCKR*	C	0.61	T	0.45	.12	-	2E-31	11663
rs780093	27,596,107	0.97	rs780094	27,594,741	23726366	Triglycerides	*GCKR*	C	0.61	C	0.358	.07	-	7E-9	1366
rs780093	27,596,107	0.97	rs780094	27,594,741	19060911	Triglycerides	*GCKR*	C	0.61	G	0.63	.10	+	3E-20	1366
rs780093	27,596,107	0.97	rs780094	27,594,741	18193044	Triglycerides	*GCKR*	C	0.61	T	0.34	.13	-	3E-14	1366
rs780093	27,596,107	0.97	rs780094	27,594,741	18193043	Triglycerides	*GCKR*	C	0.61	T	0.39	8.59	-	6E-32	1366
rs780093	27,596,107	0.90	rs1260333	27,602,108	20864672	Triglycerides	*GCKR*	C	0.61	C	0.55	.05	+	2E-19	6001
rs780093	27,596,107	1.00	rs780093	27,596,107	21386085	Triglycerides-Blood Pressure (TG-BP)	*GCKR*	C	0.61	A	NR	.18	-	3E-10	0
rs780093	27,596,107	0.90	rs1260326	27,584,444	20081857	Two-hour glucose challenge	*GCKR*	C	0.61	T	NR	.07	-	3E-10	11663
rs780093	27,596,107	0.90	rs1260326	27,584,444	23263486	Urate levels	*GCKR*	C	0.61	T	0.41	.07	-	1E-44	11663
rs780093	27,596,107	1.00	rs780093	27,596,107	20884846	Urate levels	*GCKR*	C	0.61	T	0.40	5.15	-	4E-17	0
rs780093	27,596,107	0.97	rs780094	27,594,741	19503597	Uric acid levels	*GCKR*	C	0.61	T	0.42	.05	-	1E-9	1366
rs780093	27,596,107	1.00	rs780093	27,596,107	21386085	Waist Circumference - Triglycerides (WC-TG)	*GCKR*	C	0.61	A	NR	.19	-	2E-12	0
rs780093	27,596,107	0.90	rs1260326	27,584,444	18454146	Waist circumference and related phenotypes	*GCKR*	C	0.61	NR	NR	NR	N/A	4E-8	11663

Supplementary Table 12. Genes prioritized by DEPICT for loci that reached P<1x10^{-5} with leptin in the BMI-unadjusted meta-analysis.

SNP	Chr	Region start	Region end	No. genes in locus	Gene symbol	Gene P value	False discovery rate
rs6738627	2	165510134	165700189	1	*COBLL1*	0.012	0.95
rs900400	3	156799456	156840793	2	-	0.012	0.48
rs4722501	7	25632971	25790614	1	-	0.085	1
rs11646653	16	28477983	29002104	15	*RABEP2*	0.103	1
rs717339	2	165752696	165812035	1	*SLC38A11*	0.138	1
rs10487505	7	127881337	127897681	1	*LEP*	0.153	1
rs780093	2	27505260	27858041	14	*GCKR*	0.156	1
rs11646653	16	28477983	29002104	15	*SULT1A2*	0.168	1
rs900400	3	156799456	156840793	2	-	0.184	1
rs11646653	16	28477983	29002104	15	*NUPR1*	0.264	1
rs11646653	16	28477983	29002104	15	*ATP2A1*	0.277	1
rs6071166	20	37353105	37358015	1	*SLC32A1*	0.34	1
rs7593751	2	228474806	228498036	1	*C2orf83*	0.371	1
rs11646653	16	28477983	29002104	15	*SH2B1*	0.389	1
rs780093	2	27505260	27858041	14	*C2orf16*	0.423	1
rs780093	2	27505260	27858041	14	*TRIM54*	0.446	1
rs11646653	16	28477983	29002104	15	*ATXN2L*	0.502	1
rs6444289	3	187871072	188608460	1	*LPP*	0.513	1
rs11646653	16	28477983	29002104	15	*CLN3*	0.518	1
rs11646653	16	28477983	29002104	15	*APOBR*	0.539	1
rs780093	2	27505260	27858041	14	*NRBP1*	0.541	1
rs11646653	16	28477983	29002104	15	*LAT*	0.555	1
rs11646653	16	28477983	29002104	15	*NFATC2IP*	0.566	1
rs780093	2	27505260	27858041	14	*KRTCAP3*	0.594	1
rs780093	2	27505260	27858041	14	*ZNF512*	0.66	1
rs780093	2	27505260	27858041	14	*FNDC4*	0.674	1
rs11646653	16	28477983	29002104	15	*CD19*	0.691	1
rs11646653	16	28477983	29002104	15	*IL27*	0.7	1
rs780093	2	27505260	27858041	14	*SNX17*	0.733	1
rs780093	2	27505260	27858041	14	*ZNF513*	0.787	1
rs780093	2	27505260	27858041	14	*IFT172*	0.798	1
rs9077	1	202091972	202130716	3	*PTPN7*	0.816	1
rs9077	1	202091972	202130716	3	*ARL8A*	0.85	1
rs780093	2	27505260	27858041	14	*EIF2B4*	0.868	1
rs780093	2	27505260	27858041	14	*PPM1G*	0.884	1
rs11646653	16	28477983	29002104	15	*CCDC101*	0.895	1
rs11646653	16	28477983	29002104	15	*TUFM*	0.907	1
rs11646653	16	28477983	29002104	15	*SPNS1*	0.908	1
rs9077	1	202091972	202130716	3	*GPR37L1*	0.912	1
rs8043757	16	53633824	53737771	1	*RPGRIP1L*	0.922	1
rs780093	2	27505260	27858041	14	*GTF3C2*	0.936	1
rs780093	2	27505260	27858041	14	-	0.957	1

Supplementary Table 13. Genes prioritized by DEPICT for loci that reached P<1x10^{-5} with leptin in the BMI-adjusted meta-analysis.

SNP	Chr	Region start	Region end	No. genes in locus	Gene symbol	Gene P value	False discovery rate
rs900400	3	156799456	156840793	2	-	0.01	0.35
rs780094	2	27505260	27858041	14	*KRTCAP3*	0.075	1
rs2113060	5	37106330	37753537	3	*NUP155*	0.118	1
rs780094	2	27505260	27858041	14	*ZNF512*	0.168	1
rs4842841	15	84322838	85123406	3	-	0.176	1
rs780094	2	27505260	27858041	14	*IFT172*	0.199	1
rs11597063	10	7487520	7513904	1	-	0.21	1
rs10487505	7	127881337	127897681	1	*LEP*	0.248	1
rs780094	2	27505260	27858041	14	*FNDC4*	0.256	1
rs780094	2	27505260	27858041	14	*PPM1G*	0.291	1
rs780094	2	27505260	27858041	14	*GCKR*	0.309	1
rs2113060	5	37106330	37753537	3	*WDR70*	0.33	1
rs780094	2	27505260	27858041	14	*GTF3C2*	0.516	1
rs6071166	20	37353105	37358015	1	*SLC32A1*	0.516	1
rs2189383	5	130759614	131132756	2	*RAPGEF6*	0.532	1
rs16849413	1	162039564	162353321	1	*NOS1AP*	0.573	1
rs2113060	5	37106330	37753537	3	*C5orf42*	0.604	1
rs780094	2	27505260	27858041	14	*EIF2B4*	0.648	1
rs7739842	6	131894284	132068553	3	*MED23*	0.661	1
rs2189383	5	130759614	131132756	2	*FNIP1*	0.674	1
rs780094	2	27505260	27858041	14	*C2orf16*	0.696	1
rs17526000	10	96997329	97050781	1	*PDLIM1*	0.705	1
rs4842841	15	84322838	85123406	3	-	0.719	1
rs6738627	2	165510134	165700189	1	*COBLL1*	0.728	1
rs12443533	16	18995274	19091417	2	*TMC7*	0.743	1
rs900400	3	156799456	156840793	2	-	0.748	1
rs12443533	16	18995274	19091417	2	*COQ7*	0.784	1
rs780094	2	27505260	27858041	14	*NRBP1*	0.81	1
rs780094	2	27505260	27858041	14	*SNX17*	0.825	1
rs780094	2	27505260	27858041	14	-	0.84	1
rs1304218-rs1	7	49890017	50160925	1	*ZPBP*	0.853	1
rs2035794	4	130645326	130692636	1	-	0.858	1
rs780094	2	27505260	27858041	14	*ZNF513*	0.868	1
rs9947679	18	40847859	40857615	1	*SYT4*	0.88	1
rs7739842	6	131894284	132068553	3	*ARG1*	0.886	1
rs7739842	6	131894284	132068553	3	*ENPP3*	0.904	1
rs6973074	7	153584182	154685995	1	*DPP6*	0.91	1
rs1838932	7	130417401	130418888	1	*KLF14*	0.928	1
rs780094	2	27505260	27858041	14	*TRIM54*	0.958	1
rs4842841	15	84322838	85123406	3	*ADAMTSL3*	0.992	1

Supplementary Table 14. Tissue enrichment in DEPICT analysis for loci that reached $P<1x10^{-5}$ with leptin in the BMI-unadjusted meta-analysis.

MeSH ID	Name	MeSH first level term	MeSH second level term	P value	FDR
A04.623.603	Oropharynx	Respiratory System	Pharynx	0.05	1
A15.382.520.604.800	Palatine Tonsil	Hemic and Immune Systems	Immune System	0.05	1
A06.407.071.140	Adrenal Cortex	Endocrine System	Endocrine Glands	0.08	1
A06.407.071	Adrenal Glands	Endocrine System	Endocrine Glands	0.08	1
A11.436.348	Hepatocytes	Cells	Epithelial Cells	0.09	1
A08.186.211.730.317.357.352.435	Hypothalamo Hypophyseal System	Nervous System	Central Nervous System	0.09	1
A08.713	Neurosecretory Systems	Nervous System	Neurosecretory Systems	0.09	1
A08.186.211.730.317.357.352	Hypothalamus Middle	Nervous System	Central Nervous System	0.09	1
A05.360.319.679.690	Myometrium	Urogenital System	Genitalia	0.10	1
A05.360.319.114.630.535	Ovarian Follicle	Urogenital System	Genitalia	0.10	1
A06.407.312.497.535.300.500	Cumulus Cells	Endocrine System	Endocrine Glands	0.10	1
A11.436.329	Granulosa Cells	Cells	Epithelial Cells	0.10	1
A07.541.510.110	Aortic Valve	Cardiovascular System	Heart	0.11	1
A07.541.510	Heart Valves	Cardiovascular System	Heart	0.11	1
A10.165.114.830.750	Subcutaneous Fat	Tissues	Connective Tissue	0.11	1
A10.165.114.830	Adipose Tissue White	Tissues	Connective Tissue	0.11	1
A10.165.114	Adipose Tissue	Tissues	Connective Tissue	0.11	1
A10.165.114.830.500	Abdominal Fat	Tissues	Connective Tissue	0.13	1
A10.165.114.830.500.750	Subcutaneous Fat Abdominal	Tissues	Connective Tissue	0.13	1
A11.872.378.294	Lymphoid Progenitor Cells	Cells	Stem Cells	0.13	1
A11.118.637.555.567.562.440	Precursor Cells B Lymphoid	Cells	Blood Cells	0.13	1
A08.186.211.730.317.357	Hypothalamus	Nervous System	Central Nervous System	0.14	1
A11.382	Endocrine Cells	Cells	Endocrine Cells	0.14	1
A08.186.211.730.317	Diencephalon	Nervous System	Central Nervous System	0.16	1
A03.556.875.875	Stomach	Digestive System	Gastrointestinal Tract	0.17	1
A03.556.875	Upper Gastrointestinal Tract	Digestive System	Gastrointestinal Tract	0.18	1
A03.556.875.500	Esophagus	Digestive System	Gastrointestinal Tract	0.18	1
A03.556.500.760	Salivary Glands	Digestive System	Gastrointestinal Tract	0.18	1
A03.556.500.760.464	Parotid Gland	Digestive System	Gastrointestinal Tract	0.19	1
A10.690.467	Muscle Smooth	Tissues	Muscles	0.19	1
A07.231.114	Arteries	Cardiovascular System	Blood Vessels	0.20	1
A11.329.114	Adipocytes	Cells	Connective Tissue Cells	0.20	1
A07.231	Blood Vessels	Cardiovascular System	Blood Vessels	0.21	1
A10.336	Exocrine Glands	Tissues	Exocrine Glands	0.22	1
A11.497.497.600	Oocytes	Cells	Germ Cells	0.25	1
A05.360.490.690	Ovum	Urogenital System	Genitalia	0.25	1
A10.336.707	Prostate	Tissues	Exocrine Glands	0.25	1
A02.165	Cartilage	Musculoskeletal System	Cartilage	0.27	1
A05.810.453.324	Kidney Cortex	Urogenital System	Urinary Tract	0.27	1
A03.734	Pancreas	Digestive System	Pancreas	0.28	1
A07.231.908	Veins	Cardiovascular System	Blood Vessels	0.29	1
A08.186.211.730.885.287.500.571.[illegible]	Visual Cortex	Nervous System	Central Nervous System	0.29	1
A05.360.490	Germ Cells	Urogenital System	Genitalia	0.29	1
A03.620	Liver	Digestive System	Liver	0.29	1
A08.186.211.730.885.287.500.571	Occipital Lobe	Nervous System	Central Nervous System	0.30	1
A11.872.653	Neural Stem Cells	Cells	Stem Cells	0.30	1
A05.360.444	Genitalia Male	Urogenital System	Genitalia	0.30	1
A08.186.211.730.885.287.500.270	Frontal Lobe	Nervous System	Central Nervous System	0.31	1
A11.436.294.064	Glucagon Secreting Cells	Cells	Epithelial Cells	0.32	1
A11.382.625	Enteroendocrine Cells	Cells	Endocrine Cells	0.32	1
A08.186.211.653	Mesencephalon	Nervous System	Central Nervous System	0.32	1
A11.329.171	Chondrocytes	Cells	Connective Tissue Cells	0.32	1

MeSH ID	Name	MeSH first level term	MeSH second level term	P value	FDR
A05.360.444.492.362	Foreskin	Urogenital System	Genitalia	0.33	1
A05.360.444.492	Penis	Urogenital System	Genitalia	0.33	1
A10.615.284.473	Chorion	Tissues	Membranes	0.33	1
A10.615.284	Extraembryonic Membranes	Tissues	Membranes	0.33	1
A11.620.520	Myocytes Smooth Muscle	Cells	Muscle Cells	0.34	1
A11.620	Muscle Cells	Cells	Muscle Cells	0.34	1
A08.186	Central Nervous System	Nervous System	Central Nervous System	0.35	1
A08.186.211	Brain	Nervous System	Central Nervous System	0.35	1
A07.541.358.100	Atrial Appendage	Cardiovascular System	Heart	0.35	1
A08.186.211.730	Prosencephalon	Nervous System	Central Nervous System	0.36	1
A07.541.358	Heart Atria	Cardiovascular System	Heart	0.36	1
A14.724	Pharynx	Stomatognathic System	Pharynx	0.38	1
A05.810.453	Kidney	Urogenital System	Urinary Tract	0.38	1
A14.549	Mouth	Stomatognathic System	Mouth	0.39	1
A03.734.414	Islets of Langerhans	Digestive System	Pancreas	0.39	1
A05.810	Urinary Tract	Urogenital System	Urinary Tract	0.39	1
A08.186.211.730.885.287.500	Cerebral Cortex	Nervous System	Central Nervous System	0.40	1
A10.615.550.760	Respiratory Mucosa	Tissues	Membranes	0.40	1
A09.531	Nose	Sense Organs	Nose	0.40	1
A04.531.520	Nasal Mucosa	Respiratory System	Nose	0.40	1
A10.615.550.599	Mouth Mucosa	Tissues	Membranes	0.40	1
A11.627.624.249	Monocyte Macrophage Precursor Cells	Cells	Myeloid Cells	0.40	1
A08.186.211.730.885	Telencephalon	Nervous System	Central Nervous System	0.40	1
A11.118.637.555.567.562	B Lymphocytes	Cells	Blood Cells	0.41	1
A11.063	Antibody Producing Cells	Cells	Antibody-Producing Cells	0.41	1
A08.186.211.730.885.287	Cerebrum	Nervous System	Central Nervous System	0.41	1
A08.186.211.730.885.287.500.863	Temporal Lobe	Nervous System	Central Nervous System	0.42	1
A08.186.211.464.710.225	Entorhinal Cortex	Nervous System	Central Nervous System	0.43	1
A08.186.211.464.710	Parahippocampal Gyrus	Nervous System	Central Nervous System	0.43	1
A07.231.908.670	Portal System	Cardiovascular System	Blood Vessels	0.43	1
A07.231.908.670.874	Umbilical Veins	Cardiovascular System	Blood Vessels	0.43	1
A05.360	Genitalia	Urogenital System	Genitalia	0.44	1
A11.329.228	Fibroblasts	Cells	Connective Tissue Cells	0.44	1
A05.360.319.679	Uterus	Urogenital System	Genitalia	0.45	1
A08.186.211.464	Limbic System	Nervous System	Central Nervous System	0.45	1
A02.835.583.443.800.800	Synovial Fluid	Musculoskeletal System	Skeleton	0.45	1
A11.872.190.260	Embryoid Bodies	Cells	Stem Cells	0.45	1
A03.556.124.684	Intestine Small	Digestive System	Gastrointestinal Tract	0.48	1
A15.145.229.188	Blood Platelets	Hemic and Immune Systems	Blood	0.48	1
A03.556.249.124	Ileum	Digestive System	Gastrointestinal Tract	0.48	1
A07.541	Heart	Cardiovascular System	Heart	0.49	1
A11.329.629	Osteoblasts	Cells	Connective Tissue Cells	0.49	1
A08.186.211.730.885.287.500.670	Parietal Lobe	Nervous System	Central Nervous System	0.50	1
A03.556.124.526.767	Rectum	Digestive System	Gastrointestinal Tract	0.50	1
A17.815	Skin	Integumentary System	Skin	0.50	1
A15.145.229.637.555.567.569.200	CD4 Positive T Lymphocytes	Hemic and Immune Systems	Blood	0.51	1
A15.382.520.604.700	Spleen	Hemic and Immune Systems	Immune System	0.51	1
A11.872.580	Mesenchymal Stem Cells	Cells	Stem Cells	0.51	1
A15.145.846	Serum	Hemic and Immune Systems	Blood	0.51	1
A03.556.249.249.209	Cecum	Digestive System	Gastrointestinal Tract	0.52	1
A11.118.637.555.567.569.200.700	T Lymphocytes Regulatory	Cells	Blood Cells	0.53	1
A10.549	Lymphoid Tissue	Tissues	Lymphoid Tissue	0.53	1

MeSH ID	Name	MeSH first level term	MeSH second level term	P value	FDR
A15.382.520	Lymphatic System	Hemic and Immune Systems	Immune System	0.53	1
A08.186.211.464.405	Hippocampus	Nervous System	Central Nervous System	0.53	1
A08.186.211.730.885.287.249	Basal Ganglia	Nervous System	Central Nervous System	0.54	1
A11.329.830	Stromal Cells	Cells	Connective Tissue Cells	0.54	1
A15.382.490.555.567	Lymphocytes	Hemic and Immune Systems	Immune System	0.54	1
A10.272	Epithelium	Tissues	Epithelium	0.54	1
A07.541.560	Heart Ventricles	Cardiovascular System	Heart	0.54	1
A10.615.789	Serous Membrane	Tissues	Membranes	0.55	1
A05.360.319.114.373	Fallopian Tubes	Urogenital System	Genitalia	0.56	1
A15.382.490.555.567.622	Lymphocytes Null	Hemic and Immune Systems	Immune System	0.57	1
A05.360.319	Genitalia Female	Urogenital System	Genitalia	0.57	1
A11.436	Epithelial Cells	Cells	Epithelial Cells	0.57	1
A10.615.550	Mucous Membrane	Tissues	Membranes	0.57	1
A10.615	Membranes	Tissues	Membranes	0.58	1
A03.556	Gastrointestinal Tract	Digestive System	Gastrointestinal Tract	0.58	1
A03.556.249	Lower Gastrointestinal Tract	Digestive System	Gastrointestinal Tract	0.59	1
A03.556.249.249	Intestine Large	Digestive System	Gastrointestinal Tract	0.60	1
A02.835.583.443.800	Synovial Membrane	Musculoskeletal System	Skeleton	0.60	1
A02.835.583	Joints	Musculoskeletal System	Skeleton	0.60	1
A02.835.583.443	Joint Capsule	Musculoskeletal System	Skeleton	0.60	1
A08.186.211.730.885.287.249.487	Corpus Striatum	Nervous System	Central Nervous System	0.60	1
A03.556.249.249.356	Colon	Digestive System	Gastrointestinal Tract	0.61	1
A08.186.211.132	Brain Stem	Nervous System	Central Nervous System	0.61	1
A11.872.378.590.635	Granulocyte Macrophage Progenitor Cells	Cells	Stem Cells	0.61	1
A15.145.693	Plasma	Hemic and Immune Systems	Blood	0.61	1
A05.360.319.679.490	Endometrium	Urogenital System	Genitalia	0.62	1
A15.145.229.637.555.567.562.725	Plasma Cells	Hemic and Immune Systems	Blood	0.62	1
A14.549.885	Tongue	Stomatognathic System	Mouth	0.62	1
A03.556.249.249.356.668	Colon Sigmoid	Digestive System	Gastrointestinal Tract	0.62	1
A03.556.124	Intestines	Digestive System	Gastrointestinal Tract	0.62	1
A11.329	Connective Tissue Cells	Cells	Connective Tissue Cells	0.63	1
A15.145.229.637.555	Leukocytes Mononuclear	Hemic and Immune Systems	Blood	0.63	1
A10.165.450.300	Cicatrix	Tissues	Connective Tissue	0.64	1
A10.165.450	Granulation Tissue	Tissues	Connective Tissue	0.64	1
A15.382.490.315.583	Neutrophils	Hemic and Immune Systems	Immune System	0.64	1
A06.407	Endocrine Glands	Endocrine System	Endocrine Glands	0.65	1
A05.360.319.114	Adnexa Uteri	Urogenital System	Genitalia	0.65	1
A05.360.319.114.630	Ovary	Urogenital System	Genitalia	0.65	1
A04.411	Lung	Respiratory System	Lung	0.66	1
A03.556.124.369	Intestinal Mucosa	Digestive System	Gastrointestinal Tract	0.66	1
A11.118.637	Leukocytes	Cells	Blood Cells	0.66	1
A10.549.400	Lymph Nodes	Tissues	Lymphoid Tissue	0.67	1
A11.118.637.555.567.569	T Lymphocytes	Cells	Blood Cells	0.67	1
A08.186.211.865	Rhombencephalon	Nervous System	Central Nervous System	0.67	1
A08.186.211.865.428	Metencephalon	Nervous System	Central Nervous System	0.67	1
A15.145	Blood	Hemic and Immune Systems	Blood	0.67	1
A11.118.637.415	Granulocytes	Cells	Blood Cells	0.68	1
A10.165.450.300.425	Keloid	Tissues	Connective Tissue	0.68	1
A11.436.275	Endothelial Cells	Cells	Epithelial Cells	0.69	1
A06.407.312	Gonads	Endocrine System	Endocrine Glands	0.69	1
A15.145.229	Blood Cells	Hemic and Immune Systems	Blood	0.70	1

MeSH ID	Name	MeSH first level term	MeSH second level term	P value	FDR
A15.382.812.260	Dendritic Cells	Hemic and Immune Systems	Immune System	0.70	1
A11.066	Antigen Presenting Cells	Cells	Antigen-Presenting Cells	0.70	1
A15.145.300	Fetal Blood	Hemic and Immune Systems	Blood	0.71	1
A11.872.378	Hematopoietic Stem Cells	Cells	Stem Cells	0.71	1
A14.724.557	Nasopharynx	Stomatognathic System	Pharynx	0.72	1
A11.436.397	Keratinocytes	Cells	Epithelial Cells	0.73	1
A15.382	Immune System	Hemic and Immune Systems	Immune System	0.73	1
A10.165	Connective Tissue	Tissues	Connective Tissue	0.73	1
A15.382.216	Bone Marrow	Hemic and Immune Systems	Immune System	0.73	1
A02.835.232.834	Spine	Musculoskeletal System	Skeleton	0.73	1
A15.378.316.580	Monocytes	Hemic and Immune Systems	Hematopoietic System	0.73	1
A02.835.232.834.151	Cervical Vertebrae	Musculoskeletal System	Skeleton	0.73	1
A08.186.211.132.810.428.200	Cerebellum	Nervous System	Central Nervous System	0.73	1
A15.382.680	Phagocytes	Hemic and Immune Systems	Immune System	0.74	1
A09.371.729	Retina	Sense Organs	Eye	0.75	1
A15.378	Hematopoietic System	Hemic and Immune Systems	Hematopoietic System	0.75	1
A15.378.316	Bone Marrow Cells	Hemic and Immune Systems	Hematopoietic System	0.75	1
A15.382.490.555.567.537	Killer Cells Natural	Hemic and Immune Systems	Immune System	0.76	1
A15.382.812.522	Macrophages	Hemic and Immune Systems	Immune System	0.76	1
A05.810.890	Urinary Bladder	Urogenital System	Urinary Tract	0.76	1
A11.872.378.590.817	Megakaryocyte Erythroid Progenitor Cells	Cells	Stem Cells	0.76	1
A15.378.316.378.590.837.250	Erythroid Precursor Cells	Hemic and Immune Systems	Hematopoietic System	0.76	1
A15.382.812	Mononuclear Phagocyte System	Hemic and Immune Systems	Immune System	0.77	1
A11.627.635	Myeloid Progenitor Cells	Cells	Myeloid Cells	0.77	1
A02.835.232	Bone and Bones	Musculoskeletal System	Skeleton	0.77	1
A11.627	Myeloid Cells	Cells	Myeloid Cells	0.78	1
A02.835	Skeleton	Musculoskeletal System	Skeleton	0.78	1
A05.360.319.679.256	Cervix Uteri	Urogenital System	Genitalia	0.79	1
A10.690	Muscles	Tissues	Muscles	0.79	1
A14.549.167	Dentition	Stomatognathic System	Mouth	0.79	1
A11.872	Stem Cells	Cells	Stem Cells	0.80	1
A15.145.229.334	Erythrocytes	Hemic and Immune Systems	Blood	0.80	1
A05.360.319.887	Vulva	Urogenital System	Genitalia	0.81	1
A11.443	Erythroid Cells	Cells	Erythroid Cells	0.81	1
A11.329.372.600	Macrophages Alveolar	Cells	Connective Tissue Cells	0.83	1
A10.272.497	Epidermis	Tissues	Epithelium	0.86	1
A11.627.340.360	Granulocyte Precursor Cells	Cells	Myeloid Cells	0.87	1
A02.835.232.043.300	Foot Bones	Musculoskeletal System	Skeleton	0.88	1
A02.835.232.043.300.710	Tarsal Bones	Musculoskeletal System	Skeleton	0.88	1
A09.371.060	Anterior Eye Segment	Sense Organs	Eye	0.88	1
A14.549.167.646	Periodontium	Stomatognathic System	Mouth	0.88	1
A09.371.337.168	Conjunctiva	Sense Organs	Eye	0.89	1
A09.371.337	Eyelids	Sense Organs	Eye	0.89	1
A11.872.700	Pluripotent Stem Cells	Cells	Stem Cells	0.89	1
A11.872.700.500	Induced Pluripotent Stem Cells	Cells	Stem Cells	0.89	1
A02.835.232.043	Bones of Lower Extremity	Musculoskeletal System	Skeleton	0.89	1
A11.872.190	Embryonic Stem Cells	Cells	Stem Cells	0.90	1
A10.690.552.500	Muscle Skeletal	Tissues	Muscles	0.91	1
A10.690.552	Muscle Striated	Tissues	Muscles	0.91	1
A02.633.567.850	Quadriceps Muscle	Musculoskeletal System	Muscles	0.93	1
A06.407.312.782	Testis	Endocrine System	Endocrine Glands	0.95	1
A06.407.900	Thyroid Gland	Endocrine System	Endocrine Glands	0.99	1
A11.872.040	Adult Stem Cells	Cells	Stem Cells	0.99	1
A09.371	Eye	Sense Organs	Eye	1.00	1

Supplementary Table 15. Tissue enrichment in DEPICT analysis for loci that reached $P<1x10^{-5}$ with leptin in the BMI-adjusted meta-analysis.

MeSH ID	Name	MeSH first level term	MeSH second level term	P value	FDR
A11.436.348	Hepatocytes	Cells	Epithelial Cells	0.0140	1
A08.713	Neurosecretory Systems	Nervous System	Neurosecretory Systems	0.0400	1
A08.186.211.730.317.357.352	Hypothalamus Middle	Nervous System	Central Nervous System	0.0400	1
A08.186.211.730.317.357.352.435	Hypothalamo Hypophyseal System	Nervous System	Central Nervous System	0.0400	1
A11.118.637.555.567.562.440	Precursor Cells B Lymphoid	Cells	Blood Cells	0.0429	1
A11.872.378.294	Lymphoid Progenitor Cells	Cells	Stem Cells	0.0429	1
A11.382.625	Enteroendocrine Cells	Cells	Endocrine Cells	0.0857	1
A11.436.294.064	Glucagon Secreting Cells	Cells	Epithelial Cells	0.0857	1
A08.186.211.730.317.357	Hypothalamus	Nervous System	Central Nervous System	0.0932	1
A10.165	Connective Tissue	Tissues	Connective Tissue	0.10	1
A10.165.114.830.500.750	Subcutaneous Fat Abdominal	Tissues	Connective Tissue	0.10	1
A10.165.114.830.500	Abdominal Fat	Tissues	Connective Tissue	0.10	1
A11.436.397	Keratinocytes	Cells	Epithelial Cells	0.11	1
A10.165.114.830	Adipose Tissue White	Tissues	Connective Tissue	0.11	1
A10.165.114.830.750	Subcutaneous Fat	Tissues	Connective Tissue	0.11	1
A15.382.216	Bone Marrow	Hemic and Immune Systems	Immune System	0.11	1
A11.118.637.555.567.562	B Lymphocytes	Cells	Blood Cells	0.12	1
A11.063	Antibody Producing Cells	Cells	Antibody-Producing Cells	0.12	1
A10.165.114	Adipose Tissue	Tissues	Connective Tissue	0.12	1
A02.835.232	Bone and Bones	Musculoskeletal System	Skeleton	0.12	1
A02.835	Skeleton	Musculoskeletal System	Skeleton	0.13	1
A14.724	Pharynx	Stomatognathic System	Pharynx	0.13	1
A08.186.211.730.317	Diencephalon	Nervous System	Central Nervous System	0.14	1
A03.734.414	Islets of Langerhans	Digestive System	Pancreas	0.15	1
A15.145.693	Plasma	Hemic and Immune Systems	Blood	0.16	1
A15.145.229.637.555.567.562.725	Plasma Cells	Hemic and Immune Systems	Blood	0.16	1
A15.145.846	Serum	Hemic and Immune Systems	Blood	0.17	1
A11.329.114	Adipocytes	Cells	Connective Tissue Cells	0.17	1
A06.407.071	Adrenal Glands	Endocrine System	Endocrine Glands	0.20	1
A10.615.284	Extraembryonic Membranes	Tissues	Membranes	0.22	1
A10.615.284.473	Chorion	Tissues	Membranes	0.22	1
A05.360.490	Germ Cells	Urogenital System	Genitalia	0.22	1
A04.623.603	Oropharynx	Respiratory System	Pharynx	0.23	1
A15.382.520.604.800	Palatine Tonsil	Hemic and Immune Systems	Immune System	0.23	1
A11.872.653	Neural Stem Cells	Cells	Stem Cells	0.23	1
A15.382	Immune System	Hemic and Immune Systems	Immune System	0.24	1
A05.360.319.679.690	Myometrium	Urogenital System	Genitalia	0.25	1
A15.382.490.315.583	Neutrophils	Hemic and Immune Systems	Immune System	0.25	1
A15.382.490.555.567	Lymphocytes	Hemic and Immune Systems	Immune System	0.26	1
A11.118.637.415	Granulocytes	Cells	Blood Cells	0.26	1
A11.872.378.590.817	Megakaryocyte Erythroid Progenitor Cells	Cells	Stem Cells	0.26	1
A15.378.316.378.590.837.250	Erythroid Precursor Cells	Hemic and Immune Systems	Hematopoietic System	0.26	1
A07.231.908	Veins	Cardiovascular System	Blood Vessels	0.26	1
A14.724.557	Nasopharynx	Stomatognathic System	Pharynx	0.27	1
A08.186.211.865.428	Metencephalon	Nervous System	Central Nervous System	0.27	1
A08.186.211.865	Rhombencephalon	Nervous System	Central Nervous System	0.27	1
A02.633.567.850	Quadriceps Muscle	Musculoskeletal System	Muscles	0.27	1
A11.329.171	Chondrocytes	Cells	Connective Tissue Cells	0.28	1
A08.186.211.132.810.428.200	Cerebellum	Nervous System	Central Nervous System	0.28	1
A06.407.071.140	Adrenal Cortex	Endocrine System	Endocrine Glands	0.29	1
A03.620	Liver	Digestive System	Liver	0.29	1

MeSH ID	Name	MeSH first level term	MeSH second level term	P value	FDR
A08.186.211.132	Brain Stem	Nervous System	Central Nervous System	0.29	1
A10.690.552.500	Muscle Skeletal	Tissues	Muscles	0.29	1
A10.690.552	Muscle Striated	Tissues	Muscles	0.29	1
A10.272.497	Epidermis	Tissues	Epithelium	0.30	1
A07.231.908.670	Portal System	Cardiovascular System	Blood Vessels	0.31	1
A07.231.908.670.874	Umbilical Veins	Cardiovascular System	Blood Vessels	0.31	1
A11.436.275	Endothelial Cells	Cells	Epithelial Cells	0.31	1
A10.690	Muscles	Tissues	Muscles	0.31	1
A11.382	Endocrine Cells	Cells	Endocrine Cells	0.32	1
A14.549.885	Tongue	Stomatognathic System	Mouth	0.34	1
A05.360.490.690	Ovum	Urogenital System	Genitalia	0.35	1
A11.497.497.600	Oocytes	Cells	Germ Cells	0.35	1
A10.615.550.599	Mouth Mucosa	Tissues	Membranes	0.35	1
A11.118.637	Leukocytes	Cells	Blood Cells	0.36	1
A10.272	Epithelium	Tissues	Epithelium	0.36	1
A08.186.211.653	Mesencephalon	Nervous System	Central Nervous System	0.38	1
A02.835.583.443.800.800	Synovial Fluid	Musculoskeletal System	Skeleton	0.39	1
A05.810.453.324	Kidney Cortex	Urogenital System	Urinary Tract	0.39	1
A11.329.629	Osteoblasts	Cells	Connective Tissue Cells	0.40	1
A03.556.249.249.209	Cecum	Digestive System	Gastrointestinal Tract	0.40	1
A07.231	Blood Vessels	Cardiovascular System	Blood Vessels	0.40	1
A09.531	Nose	Sense Organs	Nose	0.40	1
A10.615.550.760	Respiratory Mucosa	Tissues	Membranes	0.40	1
A04.531.520	Nasal Mucosa	Respiratory System	Nose	0.40	1
A15.382.490.555.567.622	Lymphocytes Null	Hemic and Immune Systems	Immune System	0.40	1
A15.145.300	Fetal Blood	Hemic and Immune Systems	Blood	0.40	1
A09.371.729	Retina	Sense Organs	Eye	0.41	1
A03.556.875.500	Esophagus	Digestive System	Gastrointestinal Tract	0.41	1
A10.690.467	Muscle Smooth	Tissues	Muscles	0.41	1
A11.436	Epithelial Cells	Cells	Epithelial Cells	0.42	1
A14.549	Mouth	Stomatognathic System	Mouth	0.43	1
A11.118.637.555.567.569.200.700	T Lymphocytes Regulatory	Cells	Blood Cells	0.44	1
A05.360.444.492.362	Foreskin	Urogenital System	Genitalia	0.44	1
A05.360.444.492	Penis	Urogenital System	Genitalia	0.44	1
A15.145.229.637.555.567.569.200	CD4 Positive T Lymphocytes	Hemic and Immune Systems	Blood	0.45	1
A05.360.319.679.256	Cervix Uteri	Urogenital System	Genitalia	0.46	1
A03.556.124.526.767	Rectum	Digestive System	Gastrointestinal Tract	0.47	1
A11.620	Muscle Cells	Cells	Muscle Cells	0.47	1
A11.620.520	Myocytes Smooth Muscle	Cells	Muscle Cells	0.47	1
A15.145.229.637.555	Leukocytes Mononuclear	Hemic and Immune Systems	Blood	0.48	1
A11.872.190.260	Embryoid Bodies	Cells	Stem Cells	0.48	1
A17.815	Skin	Integumentary System	Skin	0.49	1
A03.734	Pancreas	Digestive System	Pancreas	0.51	1
A15.145	Blood	Hemic and Immune Systems	Blood	0.51	1
A15.145.229	Blood Cells	Hemic and Immune Systems	Blood	0.52	1
A08.186.211.464.405	Hippocampus	Nervous System	Central Nervous System	0.52	1
A10.615.550	Mucous Membrane	Tissues	Membranes	0.52	1
A11.443	Erythroid Cells	Cells	Erythroid Cells	0.52	1
A03.556.249.249	Intestine Large	Digestive System	Gastrointestinal Tract	0.53	1
A15.378	Hematopoietic System	Hemic and Immune Systems	Hematopoietic System	0.53	1
A15.378.316	Bone Marrow Cells	Hemic and Immune Systems	Hematopoietic System	0.53	1
A07.541.560	Heart Ventricles	Cardiovascular System	Heart	0.53	1

MeSH ID	Name	MeSH first level term	MeSH second level term	P value	FDR
A15.145.229.334	Erythrocytes	Hemic and Immune Systems	Blood	0.53	1
A05.360.319.679	Uterus	Urogenital System	Genitalia	0.53	1
A03.556.875	Upper Gastrointestinal Tract	Digestive System	Gastrointestinal Tract	0.53	1
A08.186.211.730	Prosencephalon	Nervous System	Central Nervous System	0.54	1
A03.556.249.249.356	Colon	Digestive System	Gastrointestinal Tract	0.54	1
A08.186	Central Nervous System	Nervous System	Central Nervous System	0.54	1
A08.186.211.730.885.287.500.270	Frontal Lobe	Nervous System	Central Nervous System	0.54	1
A08.186.211.730.885.287.500.571	Occipital Lobe	Nervous System	Central Nervous System	0.54	1
A08.186.211	Brain	Nervous System	Central Nervous System	0.54	1
A03.556.249	Lower Gastrointestinal Tract	Digestive System	Gastrointestinal Tract	0.54	1
A07.541.358.100	Atrial Appendage	Cardiovascular System	Heart	0.54	1
A06.407.312.782	Testis	Endocrine System	Endocrine Glands	0.55	1
A08.186.211.730.885.287.500.571.7	Visual Cortex	Nervous System	Central Nervous System	0.56	1
A05.360.319.679.490	Endometrium	Urogenital System	Genitalia	0.56	1
A03.556.124	Intestines	Digestive System	Gastrointestinal Tract	0.56	1
A03.556	Gastrointestinal Tract	Digestive System	Gastrointestinal Tract	0.57	1
A10.615	Membranes	Tissues	Membranes	0.57	1
A10.165.450.300.425	Keloid	Tissues	Connective Tissue	0.58	1
A08.186.211.464	Limbic System	Nervous System	Central Nervous System	0.58	1
A07.541.358	Heart Atria	Cardiovascular System	Heart	0.58	1
A08.186.211.730.885.287.500	Cerebral Cortex	Nervous System	Central Nervous System	0.58	1
A03.556.249.249.356.668	Colon Sigmoid	Digestive System	Gastrointestinal Tract	0.58	1
A08.186.211.730.885	Telencephalon	Nervous System	Central Nervous System	0.59	1
A08.186.211.730.885.287	Cerebrum	Nervous System	Central Nervous System	0.59	1
A03.556.124.684	Intestine Small	Digestive System	Gastrointestinal Tract	0.59	1
A07.541	Heart	Cardiovascular System	Heart	0.59	1
A10.615.789	Serous Membrane	Tissues	Membranes	0.60	1
A05.810.453	Kidney	Urogenital System	Urinary Tract	0.60	1
A05.810	Urinary Tract	Urogenital System	Urinary Tract	0.60	1
A05.810.890	Urinary Bladder	Urogenital System	Urinary Tract	0.61	1
A03.556.875.875	Stomach	Digestive System	Gastrointestinal Tract	0.61	1
A05.360.444	Genitalia Male	Urogenital System	Genitalia	0.61	1
A02.835.232.043.300	Foot Bones	Musculoskeletal System	Skeleton	0.62	1
A02.835.232.043.300.710	Tarsal Bones	Musculoskeletal System	Skeleton	0.62	1
A08.186.211.730.885.287.500.863	Temporal Lobe	Nervous System	Central Nervous System	0.62	1
A02.835.583.443	Joint Capsule	Musculoskeletal System	Skeleton	0.63	1
A02.835.583.443.800	Synovial Membrane	Musculoskeletal System	Skeleton	0.63	1
A02.835.583	Joints	Musculoskeletal System	Skeleton	0.63	1
A07.231.114	Arteries	Cardiovascular System	Blood Vessels	0.63	1
A02.835.232.043	Bones of Lower Extremity	Musculoskeletal System	Skeleton	0.63	1
A11.329.228	Fibroblasts	Cells	Connective Tissue Cells	0.63	1
A10.336.707	Prostate	Tissues	Exocrine Glands	0.63	1
A08.186.211.730.885.287.249	Basal Ganglia	Nervous System	Central Nervous System	0.63	1
A10.165.450	Granulation Tissue	Tissues	Connective Tissue	0.64	1
A10.165.450.300	Cicatrix	Tissues	Connective Tissue	0.64	1
A11.627	Myeloid Cells	Cells	Myeloid Cells	0.64	1
A08.186.211.464.710	Parahippocampal Gyrus	Nervous System	Central Nervous System	0.65	1
A08.186.211.464.710.225	Entorhinal Cortex	Nervous System	Central Nervous System	0.65	1
A15.382.680	Phagocytes	Hemic and Immune Systems	Immune System	0.65	1
A03.556.124.369	Intestinal Mucosa	Digestive System	Gastrointestinal Tract	0.65	1
A10.336	Exocrine Glands	Tissues	Exocrine Glands	0.66	1

MeSH ID	Name	MeSH first level term	MeSH second level term	P value	FDR
A11.627.340.360	Granulocyte Precursor Cells	Cells	Myeloid Cells	0.66	1
A15.382.520	Lymphatic System	Hemic and Immune Systems	Immune System	0.66	1
A10.549	Lymphoid Tissue	Tissues	Lymphoid Tissue	0.66	1
A11.118.637.555.567.569	T Lymphocytes	Cells	Blood Cells	0.68	1
A15.382.520.604.700	Spleen	Hemic and Immune Systems	Immune System	0.68	1
A09.371.337.168	Conjunctiva	Sense Organs	Eye	0.69	1
A09.371.337	Eyelids	Sense Organs	Eye	0.69	1
A09.371.060	Anterior Eye Segment	Sense Organs	Eye	0.69	1
A08.186.211.730.885.287.500.670	Parietal Lobe	Nervous System	Central Nervous System	0.69	1
A08.186.211.730.885.287.249.487	Corpus Striatum	Nervous System	Central Nervous System	0.69	1
A05.360.319.114.630.535	Ovarian Follicle	Urogenital System	Genitalia	0.70	1
A11.436.329	Granulosa Cells	Cells	Epithelial Cells	0.70	1
A06.407.312.497.535.300.500	Cumulus Cells	Endocrine System	Endocrine Glands	0.70	1
A15.382.490.555.567.537	Killer Cells Natural	Hemic and Immune Systems	Immune System	0.71	1
A04.411	Lung	Respiratory System	Lung	0.72	1
A14.549.167.646	Periodontium	Stomatognathic System	Mouth	0.72	1
A05.360	Genitalia	Urogenital System	Genitalia	0.72	1
A11.329.372.600	Macrophages Alveolar	Cells	Connective Tissue Cells	0.73	1
A11.872.190	Embryonic Stem Cells	Cells	Stem Cells	0.74	1
A06.407.900	Thyroid Gland	Endocrine System	Endocrine Glands	0.74	1
A05.360.319.887	Vulva	Urogenital System	Genitalia	0.74	1
A06.407	Endocrine Glands	Endocrine System	Endocrine Glands	0.74	1
A10.549.400	Lymph Nodes	Tissues	Lymphoid Tissue	0.77	1
A11.872.700	Pluripotent Stem Cells	Cells	Stem Cells	0.77	1
A11.872.700.500	Induced Pluripotent Stem Cells	Cells	Stem Cells	0.77	1
A11.872.378	Hematopoietic Stem Cells	Cells	Stem Cells	0.77	1
A07.541.510.110	Aortic Valve	Cardiovascular System	Heart	0.77	1
A07.541.510	Heart Valves	Cardiovascular System	Heart	0.77	1
A05.360.319	Genitalia Female	Urogenital System	Genitalia	0.77	1
A06.407.312	Gonads	Endocrine System	Endocrine Glands	0.79	1
A02.835.232.834.151	Cervical Vertebrae	Musculoskeletal System	Skeleton	0.79	1
A11.872	Stem Cells	Cells	Stem Cells	0.79	1
A11.872.580	Mesenchymal Stem Cells	Cells	Stem Cells	0.79	1
A02.835.232.834	Spine	Musculoskeletal System	Skeleton	0.79	1
A15.378.316.580	Monocytes	Hemic and Immune Systems	Hematopoietic System	0.82	1
A15.145.229.188	Blood Platelets	Hemic and Immune Systems	Blood	0.83	1
A11.329	Connective Tissue Cells	Cells	Connective Tissue Cells	0.83	1
A05.360.319.114.630	Ovary	Urogenital System	Genitalia	0.86	1
A05.360.319.114	Adnexa Uteri	Urogenital System	Genitalia	0.86	1
A11.627.635	Myeloid Progenitor Cells	Cells	Myeloid Cells	0.86	1
A14.549.167	Dentition	Stomatognathic System	Mouth	0.86	1
A02.165	Cartilage	Musculoskeletal System	Cartilage	0.87	1
A03.556.249.124	Ileum	Digestive System	Gastrointestinal Tract	0.87	1
A05.360.319.114.373	Fallopian Tubes	Urogenital System	Genitalia	0.89	1
A15.382.812	Mononuclear Phagocyte System	Hemic and Immune Systems	Immune System	0.89	1
A15.382.812.522	Macrophages	Hemic and Immune Systems	Immune System	0.91	1
A03.556.500.760	Salivary Glands	Digestive System	Gastrointestinal Tract	0.91	1
A11.872.040	Adult Stem Cells	Cells	Stem Cells	0.91	1
A09.371	Eye	Sense Organs	Eye	0.91	1
A11.329.830	Stromal Cells	Cells	Connective Tissue Cells	0.93	1
A03.556.500.760.464	Parotid Gland	Digestive System	Gastrointestinal Tract	0.93	1
A11.872.378.590.635	Granulocyte Macrophage Progenitor Cells	Cells	Stem Cells	0.94	1
A11.066	Antigen Presenting Cells	Cells	Antigen-Presenting Cells	0.95	1
A15.382.812.260	Dendritic Cells	Hemic and Immune Systems	Immune System	0.95	1
A11.627.624.249	Monocyte Macrophage Precursor Cells	Cells	Myeloid Cells	0.96	1

Supplementary Table 16. DEPICT with re-constituted gene network for BMI-unadjusted leptin.

Reconstituted gene set ID	Reconstituted gene set name	P value	FDR	Reconstituted gene set genes in associated loci
MP:0004939	Abnormal B Cell Morphology	3.51E-05	1	CD19 (13.01), RABEP2 (3.19), LAT (3.14), SNX17 (2.65), NRBP1 (2.03), LPP (1.85)
ENSG00000133627	ACTR3B protein complex	1.25E-04	1	PPM1G (6.28), ATP2A1 (6.04), SNX17 (3.93), GTF3C2 (3.88), ZNF513 (3.21), ZNF512 (2.99), PTPN7 (2.51), TRIM54 (2.45), LPP (2.18), ARL8A (1.72)
ENSG00000087008	ACOX3 protein complex	1.91E-04	1	ATP2A1 (2.4), NFATC2IP (2.19), TRIM54 (1.97), LPP (1.81), FNDC4 (1.74), LEP (1.71), KRTCAP3 (1.68)
GO:0043087	Regulation Of Gtpase Activity	2.88E-04	1	APOBR (2.45), SNX17 (2.15), ZNF513 (1.89)
ENSG00000077380	DYNC1I2 protein complex	4.25E-04	1	SNX17 (2.77), NRBP1 (2.29), ENSG00000241544 (1.94), EIF2B4 (1.76)
ENSG00000165309	ARMC3 protein complex	4.37E-04	1	NRBP1 (2.63), ARL8A (2.51), GCKR (2.49), LPP (1.71)
GO:0005096	Gtpase Activator Activity	4.59E-04	1	APOBR (6.03), PTPN7 (3.91), LAT (2.64), GPR37L1 (2.6), RABEP2 (2.37), ATXN2L (1.71), NRBP1 (1.7)
ENSG00000174177	CTU2 protein complex	4.65E-04	1	LAT (3.7), GCKR (3.33), PTPN7 (2.74), ZNF512 (2.63), APOBR (2.28), CLN3 (2.22), FNDC4 (2.17), LEP (2.11), TUFM (2.0), SH2B1 (1.95)
ENSG00000124214	STAU1 protein complex	4.88E-04	1	ATP2A1 (5.06), PPM1G (4.2), NRBP1 (3.67), GTF3C2 (2.93), RABEP2 (1.75)
GO:0042054	Histone Methyltransferase Activity	6.54E-04	1	ATXN2L (4.31), PPM1G (3.6), GTF3C2 (2.87), NFATC2IP (2.79), EIF2B4 (2.62), SH2B1 (2.61), CCDC101 (2.13), NRBP1 (1.99), SNX17 (1.96)
GO:0019902	Phosphatase Binding	6.90E-04	1	TRIM54 (3.29), NRBP1 (2.4), SH2B1 (1.98), PPM1G (1.89), CD19 (1.65)
ENSG00000105610	KLF1 protein complex	6.97E-04	1	ATXN2L (7.53), ARL8A (3.83), RABEP2 (3.23), PPM1G (2.82), TUFM (2.45), CCDC101 (2.29), ZNF512 (2.15), SH2B1 (1.85)
MP:0002774	Small Prostate Gland	8.19E-04	1	FNDC4 (4.52), GCKR (2.5), SNX17 (2.32), EIF2B4 (1.89), C2orf83 (1.68)
GO:0033124	Regulation Of Gtp Catabolic Process	9.16E-04	1	APOBR (2.39), SNX17 (2.28), ZNF513 (1.8)
ENSG00000072518	MARK2 protein complex	9.55E-04	1	PTPN7 (3.32), PPM1G (2.67), ATXN2L (2.54), ENSG00000243629 (2.53), APOBR (2.35), ARL8A (2.3), COBLL1 (2.21), SH2B1 (2.19), NRBP1 (2.15), SLC32A1 (1.95)

Reconstituted gene set ID	Reconstituted gene set name	P value	FDR	Reconstituted gene set genes in associated loci
MP:0001282	Short Vibrissae	0.001	1	GPR37L1 (3.11), EIF2B4 (2.28), CCDC101 (2.1), ENSG00000223561 (1.88), C2orf83 (1.7)
GO:0007015	Actin Filament Organization	0.001	1	LPP (3.66), SLC38A11 (2.08), PTPN7 (1.99), APOBR (1.91), NRBP1 (1.66)
ENSG00000100813	ACIN1 protein complex	0.001	1	CCDC101 (1.91), NFATC2IP (1.76)
GO:0043149	Stress Fiber Assembly	0.001	1	SLC38A11 (2.97), CLN3 (1.75)
ENSG00000133216	EPHB2 protein complex	0.001	1	NRBP1 (2.8), ATXN2L (2.6), PPM1G (2.56), LPP (1.72), IFT172 (1.7)
ENSG00000112038	OPRM1 protein complex	0.001	1	SLC32A1 (5.44), C2orf83 (2.46), ARL8A (2.42), LEP (2.33), LPP (2.09), SNX17 (1.86), NRBP1 (1.86), ENSG00000243629 (1.75), ATXN2L (1.69)
MP:0005065	Abnormal Neutrophil Morphology	0.001	1	PTPN7 (5.03), APOBR (3.63), LAT (2.46), RPGRIP1L (1.68), LEP (1.65)
ENSG00000163017	ACTG2 protein complex	0.001	1	PPM1G (5.21), LPP (4.46), NRBP1 (2.82), SNX17 (2.45), TUFM (2.37), ARL8A (1.87)
MP:0001764	Abnormal Homeostasis	0.002	1	SULT1A2 (4.91), LEP (3.08), GCKR (1.98), C2orf83 (1.87), ENSG00000243629 (1.84)
MP:0003419	Delayed Endochondral Bone Ossification	0.002	1	NUPR1 (3.07), LEP (2.32), SLC32A1 (1.95), LPP (1.65)
ENSG00000198211	TUBB3 protein complex	0.002	1	NRBP1 (4.37), PPM1G (4.16), SNX17 (2.95), LPP (2.83), ENSG00000243629 (2.16), ZNF512 (2.1)
ENSG00000157193	LRP8 protein complex	0.002	1	GPR37L1 (3.85), NRBP1 (3.16), EIF2B4 (3.13)
ENSG00000114670	NEK11 protein complex	0.002	1	ATP2A1 (2.14), FNDC4 (2.06), NFATC2IP (2.06), LPP (1.83)
ENSG00000139219	COL2A1 protein complex	0.002	1	NUPR1 (2.05), LAT (1.76), SLC38A11 (1.73)
ENSG00000058272	PPP1R12A protein complex	0.002	1	ATP2A1 (5.92), TRIM54 (4.72), PPM1G (4.02), NRBP1 (3.79), LPP (3.57), SNX17 (3.02), EIF2B4 (1.83)
ENSG00000149177	PTPRJ protein complex	0.002	1	CLN3 (4.43), LAT (4.28), ARL8A (2.17), IFT172 (2.16), TUFM (2.13), APOBR (2.05), CCDC101 (1.84), ATXN2L (1.78)

Reconstituted gene set ID	Reconstituted gene set name	P value	FDR	Reconstituted gene set genes in associated loci
ENSG00000103342	GSPT1 protein complex	0.002	1	TUFM (2.45), PPM1G (2.36), NRBP1 (2.25), ZNF512 (1.83), CD19 (1.72)
ENSG00000151577	DRD3 protein complex	0.002	1	LPP (2.6), LEP (2.59), COBLL1 (1.77), LAT (1.76), NRBP1 (1.75)
GO:0015085	Calcium Ion Transmembrane Transporter Activity	0.002	1	TRIM54 (3.31), LPP (2.53), SPNS1 (2.17), ENSG00000223561 (1.93), ATXN2L (1.84), LEP (1.79)
ENSG00000125753	VASP protein complex	0.002	1	APOBR (6.07), ATP2A1 (4.63), ATXN2L (4.51), LPP (4.37), LAT (3.83), PTPN7 (3.24), TRIM54 (2.7), PPM1G (2.51), CCDC101 (2.39), ARL8A (1.81)
MP:0004989	Decreased Osteoblast Cell Number	0.002	1	LEP (3.46), KRTCAP3 (2.81), SLC32A1 (2.32), ENSG00000241544 (1.92)
MP:0002896	Abnormal Bone Mineralization	0.002	1	NUPR1 (5.46), LPP (1.78)
ENSG00000047849	MAP4 protein complex	0.002	1	NRBP1 (3.66), ARL8A (2.95), PPM1G (2.75), CLN3 (2.48), APOBR (2.32), LAT (2.28), SULT1A2 (2.21)
ENSG00000100612	DHRS7 protein complex	0.002	1	NRBP1 (4.53), SNX17 (3.89), ATXN2L (3.66), GTF3C2 (3.29), TUFM (2.8), PPM1G (2.41), ARL8A (1.83), SH2B1 (1.78), RABEP2 (1.69)
ENSG00000158560	DYNC1I1 protein complex	0.003	1	ATP2A1 (6.96), ARL8A (3.48), NRBP1 (2.6), ATXN2L (1.95), RABEP2 (1.74)
GO:0051017	Actin Filament Bundle Assembly	0.003	1	SLC38A11 (3.44), LPP (2.3), APOBR (2.14), ZNF512 (1.73)
MP:0005617	Increased Susceptibility To Type Iv Hypersensitivity Reaction	0.003	1	LAT (3.3), PTPN7 (2.37), LEP (2.37), C2orf83 (2.13), C2orf16 (1.75), APOBR (1.65)
REACTOME_TRANS-GOLGI_NETWORK_VESICLE_BUDDI	Trans:Golgi Network Vesicle Budding	0.003	1	CLN3 (5.06), NRBP1 (4.0), SPNS1 (2.95), SNX17 (2.6)
REACTOME_CLATHRIN_DERIVED_VESICLE_BUDDING	Clathrin Derived Vesicle Budding	0.003	1	CLN3 (5.06), NRBP1 (4.0), SPNS1 (2.95), SNX17 (2.6)
ENSG00000156795	WDYHV1 protein complex	0.003	1	CCDC101 (3.8), NUPR1 (3.18), NFATC2IP (2.89), KRTCAP3 (2.56), SULT1A2 (2.41), ZNF512 (1.92)
ENSG00000116353	MECR protein complex	0.003	1	ATXN2L (3.03), SULT1A2 (2.55), GCKR (2.22), RPGRIP1L (1.7)
GO:0008170	N-Methyltransferase Activity	0.003	1	ATXN2L (3.53), GTF3C2 (3.3), EIF2B4 (3.16), CCDC101 (2.82), PPM1G (2.45), TUFM (2.25), SH2B1 (2.23), NFATC2IP (2.03)
ENSG00000169710	FASN protein complex	0.003	1	NRBP1 (3.69), ATP2A1 (2.72), SULT1A2 (2.01), LPP (1.96), ENSG00000243629 (1.92), ARL8A (1.78)
MP:0003795	Abnormal Bone Structure	0.003	1	NUPR1 (4.78), LPP (1.78), ENSG00000241544 (1.77)
GO:0030811	Regulation Of Nucleotide Catabolic Process	0.003	1	SNX17 (2.21), APOBR (2.0)

Supplementary Table 17. DEPICT with re-constituted gene network for BMI-adjusted leptin.

Reconstituted gene set ID	Reconstituted gene set name	P value	FDR	Reconstituted gene set genes in associated loci
ENSG00000146963	LUC7L2 protein complex	1.71E-07	0.0500	NRBP1 (2.77), SLC32A1 (2.36), SYT4 (2.36), TMC7 (2.25), COQ7 (2.14), WDR70 (1.89), PPM1G (1.84), PDLIM1 (1.74)
ENSG00000096063	SRPK1 protein complex	1.54E-05	0.4750	PPM1G (5.36), COQ7 (3.91), WDR70 (3.05), NUP155 (3.0), ZNF512 (2.31), NRBP1 (2.2)
MP:0010954	Abnormal Cellular Respiration	3.26E-05	0.4670	TRIM54 (4.88), ZNF513 (2.76), COQ7 (2.33), ZNF512 (1.93)
ENSG00000149557	FEZ1 protein complex	3.10E-04	1	COQ7 (3.31), PPM1G (2.73), ENSG00000259511 (2.44), NUP155 (1.86)
ENSG00000173534	ENSG00000173534 protein complex	4.55E-04	1	PPM1G (2.45), COQ7 (2.25), EIF2B4 (2.03), ZNF512 (2.02)
ENSG00000170847	ENSG00000170847 protein complex	4.55E-04	1	PPM1G (2.45), COQ7 (2.25), EIF2B4 (2.03), ZNF512 (2.02)
ENSG00000164708	PGAM2 protein complex	4.77E-04	1	PPM1G (3.31), TRIM54 (3.29), EIF2B4 (2.64), WDR70 (2.52), IFT172 (2.05), NRBP1 (1.97), SNX17 (1.69)
ENSG00000135698	MPHOSPH6 protein complex	5.21E-04	1	WDR70 (4.98), PPM1G (2.43), SNX17 (2.23), COQ7 (2.0), COBLL1 (1.91), NUP155 (1.82)
MP:0001721	Absent Visceral Yolk Sac Blood Islands	5.27E-04	1	IFT172 (3.23), ADAMTSL3 (3.08), NRBP1 (2.36), GCKR (2.12), NUP155 (1.96), ARG1 (1.94)
ENSG00000024422	EHD2 protein complex	5.79E-04	1	SNX17 (4.7), NRBP1 (3.92), ZNF512 (3.43), PDLIM1 (2.98), PPM1G (2.85), ENPP3 (2.27), WDR70 (2.0), IFT172 (1.66)
ENSG00000079739	PGM1 protein complex	6.19E-04	1	WDR70 (4.33), SYT4 (2.58), EIF2B4 (2.49), SNX17 (2.2), IFT172 (1.71)
ENSG00000136286	MYO1G protein complex	6.19E-04	1	COBLL1 (2.79), SNX17 (1.7)
ENSG00000072958	AP1M1 protein complex	6.30E-04	1	ZNF512 (3.12), PPM1G (2.3), NUP155 (2.26), NOS1AP (1.85), FNIP1 (1.83), SNX17 (1.83), GTF3C2 (1.8), NRBP1 (1.69)
ENSG00000112742	TTK protein complex	7.82E-04	1	DPP6 (1.85), MED23 (1.74)
ENSG00000107625	DDX50 protein complex	8.23E-04	1	PPM1G (3.27), MED23 (2.96), RAPGEF6 (2.81), NUP155 (2.71), ARG1 (2.52), GTF3C2 (2.04)

Reconstituted gene set ID	Reconstituted gene set name	P value	FDR	Reconstituted gene set genes in associated loci
ENSG00000138107	ACTR1A protein complex	9.61E-04	1	GTF3C2 (2.95), NRBP1 (2.94), PPM1G (2.85), SNX17 (2.72), IFT172 (2.34), ENSG00000259511 (2.14), NUP155 (2.0), SLC32A1 (1.82), ADAMTSL3 (1.7)
ENSG00000113068	PFDN1 protein complex	0.001	1	GTF3C2 (3.01), PPM1G (2.37), TRIM54 (2.06), ZNF512 (1.99), SLC32A1 (1.9)
MP:0000938	Motor Neuron Degeneration	0.001	1	IFT172 (2.96), EIF2B4 (2.84), DPP6 (2.77), GTF3C2 (2.62), NRBP1 (2.32), NUP155 (2.3), ENSG00000259511 (2.14), ZNF513 (2.13), TRIM54 (2.03), PPM1G (1.87)
ENSG00000196961	AP2A1 protein complex	0.001	1	MED23 (2.39), NRBP1 (2.35), ZNF512 (1.82)
GO:0046329	Negative Regulation Of Jnk Cascade	0.001	1	SNX17 (2.14)
ENSG00000007047	MARK4 protein complex	0.001	1	TRIM54 (4.38), PPM1G (4.1), SYT4 (3.38), RAPGEF6 (2.16), NRBP1 (1.81)
ENSG00000029363	BCLAF1 protein complex	0.001	1	PPM1G (2.66), PDLIM1 (2.66), WDR70 (2.64), NRBP1 (2.61), SNX17 (2.03), NUP155 (1.89), RAPGEF6 (1.67), ZNF512 (1.67)
ENSG00000149182	ARFGAP2 protein complex	0.001	1	NRBP1 (5.63), GTF3C2 (3.13), SNX17 (3.04), MED23 (3.02), FNIP1 (2.78)
ENSG00000095139	ARCN1 protein complex	0.001	1	NRBP1 (3.52), COQ7 (3.16), SNX17 (2.76), GTF3C2 (2.71), MED23 (2.66), IFT172 (2.27), EIF2B4 (1.86), WDR70 (1.8)
ENSG00000001497	LAS1L protein complex	0.001	1	GTF3C2 (3.58), WDR70 (2.71), KLF14 (2.23), C5orf42 (2.12), PPM1G (2.11), NRBP1 (1.85)
ENSG00000082805	ERC1 protein complex	0.002	1	SYT4 (3.04), KLF14 (2.86), ADAMTSL3 (2.38), RAPGEF6 (1.75)
ENSG00000167881	SRP68 protein complex	0.002	1	NRBP1 (3.94), COQ7 (3.21), IFT172 (2.31), PPM1G (2.17), WDR70 (2.11), ENSG00000232591 (1.86), COBLL1 (1.84), FNIP1 (1.75), ENPP3 (1.73), EIF2B4 (1.66)
ENSG00000111602	TIMELESS protein complex	0.002	1	NUP155 (6.3), PPM1G (4.15), GTF3C2 (1.91)
ENSG00000118705	RPN2 protein complex	0.002	1	PPM1G (6.68), SNX17 (5.31), NUP155 (3.84), NRBP1 (2.95), PDLIM1 (2.69), EIF2B4 (2.5), GTF3C2 (2.28), ZNF512 (2.13), NOS1AP (1.79)
GO:0019400	Alditol Metabolic Process	0.002	1	ENPP3 (3.57), COQ7 (2.44), LEP (2.39), ZPBP (1.89), GCKR (1.66)
GO:0006071	Glycerol Metabolic Process	0.002	1	ENPP3 (3.61), LEP (2.49), COQ7 (2.39), ZPBP (1.98)

Reconstituted gene set ID	Reconstituted gene set name	P value	FDR	Reconstituted gene set genes in associated loci
GO:0019751	Polyol Metabolic Process	0.002	1	ENPP3 (3.04), LEP (2.37), ZPBP (2.21), COQ7 (2.01), ENSG00000243629 (1.82)
GO:0030835	Negative Regulation Of Actin Filament Depolymerization	0.002	1	ENSG00000232591 (2.92), ENSG00000259728 (2.77), ENSG00000259511 (2.34), ENSG00000243629 (1.87), NRBP1 (1.67), NOS1AP (1.66)
ENSG00000154710	RABGEF1 protein complex	0.002	1	NUP155 (2.1), ENSG00000243629 (1.74), FNIP1 (1.71)
GO:0033605	Positive Regulation Of Catecholamine Secretion	0.002	1	NOS1AP (2.16), KLF14 (2.15), LEP (1.94), SYT4 (1.8), C5orf42 (1.77)
ENSG00000089009	RPL6 protein complex	0.002	1	WDR70 (2.35), NUP155 (2.34), PPM1G (1.73)
ENSG00000174444	RPL4 protein complex	0.002	1	NUP155 (4.78), PPM1G (4.76), EIF2B4 (4.13), WDR70 (4.11), GTF3C2 (2.59), NRBP1 (2.17)
ENSG00000170820	FSHR protein complex	0.002	1	PPM1G (3.5), ENSG00000243629 (3.08), SNX17 (2.66), EIF2B4 (2.01), ZNF512 (1.82), KLF14 (1.82), NRBP1 (1.72), C5orf42 (1.72), ZPBP (1.7), SYT4 (1.65)
ENSG00000197299	BLM protein complex	0.002	1	NUP155 (6.71), PPM1G (3.55), ZNF512 (3.41), RAPGEF6 (2.62), COQ7 (2.42), C5orf42 (2.24), MED23 (1.95)
ENSG00000158560	DYNC1I1 protein complex	0.002	1	PDLIM1 (3.17), SYT4 (3.09), NRBP1 (2.6), DPP6 (2.14)
ENSG00000108100	CCNY protein complex	0.002	1	MED23 (3.92), NUP155 (3.82), ZNF512 (3.74), COQ7 (2.96), GTF3C2 (2.51), SLC32A1 (2.25), PPM1G (2.18), SNX17 (2.16), COBLL1 (2.04), ZPBP (1.75)
ENSG00000067369	TP53BP1 protein complex	0.003	1	NUP155 (4.67), COQ7 (2.45), ENSG00000232591 (1.86), WDR70 (1.75)
ENSG00000066044	ELAVL1 protein complex	0.003	1	NUP155 (3.26), PPM1G (2.88), GTF3C2 (2.41), RAPGEF6 (1.98)
ENSG00000125503	PPP1R12C protein complex	0.003	1	COBLL1 (3.43), PDLIM1 (2.44), SNX17 (2.42), RAPGEF6 (1.7), SYT4 (1.66)
REACTOME_RECRUITMENT_OF_NUMA_TO_MITOTIC_CENTROSOMES	Recruitment Of Numa To Mitotic Centrosomes	0.003	1	NUP155 (3.5), GTF3C2 (2.61), MED23 (2.48)
GO:0000922	Spindle Pole	0.003	1	NUP155 (4.01), IFT172 (2.31), FNIP1 (2.18), COQ7 (1.75), PPM1G (1.69), COBLL1 (1.68)
GO:0030834	Regulation Of Actin Filament Depolymerization	0.003	1	ENSG00000232591 (2.81), ENSG00000259728 (2.59), ENSG00000259511 (2.46), NOS1AP (1.88), NRBP1 (1.66)
GO:0002792	Negative Regulation Of Peptide Secretion	0.003	1	LEP (3.04), KRTCAP3 (2.98), NOS1AP (1.87)
ENSG00000141456	ENSG00000141456 protein complex	0.003	1	GTF3C2 (4.35), WDR70 (3.58), NRBP1 (3.29), MED23 (2.98), C5orf42 (2.88), NUP155 (2.54), RAPGEF6 (2.46), FNIP1 (2.34)
ENSG00000133961	NUMB protein complex	0.003	1	ADAMTSL3 (3.24), WDR70 (1.77), ENSG00000241544 (1.67)

Supplementary Table 18. Genes prioritized by GRAIL for loci that reached P<1x10^{-5} with leptin in the BMI-unadjusted meta-analysis.

SNP	Chr	Start	Stop	No. genes in locus	Candidate gene(s)	GRAIL P-value
rs8043757	16	52354500	52406000	1	*FTO*	0.23918461
rs780093	2	27240853	27713853	23	*SLC30A3*	0.3147925
rs6071166	20	36735500	36793500	1	*SLC32A1*	0.11950529
rs900400	3	158024492	158586492	3	*CCNL1*	0.66275441
rs6738627	2	165207739	165342739	1	*COBLL1*	0.54121544
rs7593751	2	228186739	228233239	1	*DKFZp547H025*	1
rs717339	2	165443239	165538239	2	*SLC38A11*	1
rs6444289	3	189637992	189656992	1	*LPP*	1
rs9077	1	200342466	200394966	3	*ARL8A, PTPN7, GPR37L1*	1
rs4722501	7	25647785	25769785	0		N/A
rs10487505	7	127576785	127695285	1	*LEP*	1
rs11646653	16	28192500	28943500	22	*EIF3CL*	0.64698318

Supplementary Table 19. Genes prioritized by GRAIL for loci that reached $P<1x10^{-5}$ with leptin in the BMI-adjusted meta-analysis.

SNP	Chr	Start	Stop	No. Genes in locus	Candidate gene(s)	GRAIL P-value
rs10487505	7	127576785	127695285	1	*LEP*	1
rs6071166	20	36735500	36793500	1	*SLC32A1*	0.58416815
rs780094	2	27240853	27713853	23	*UCN*	0.23136092
rs11720805	3	158024492	158586492	3	*CCNL1*	0.81344738
rs1838932	7	129823285	130370285	6	*TSGA14*	0.72429227
rs12443533	16	18884000	19015000	2	*COQ7*	0.078747098
rs2035794	4	130422345	131061845	0		N/A
rs2113060	5	36820000	37790500	5	*NUP155*	0.9640783
rs867150	5	129902000	131349500	6	*RAPGEF6*	0.95222908
rs6973074	7	154108285	154139285	1	*DPP6*	1
rs11597063	10	7471500	7570500	1	*SFMBT2*	0.61749778
rs7739842	6	131938500	132018000	4	*ARG1*	0.36943389
rs16849413	1	160305966	160533466	1	*NOS1AP*	0.74201041
rs6738627	2	165207739	165342739	1	*COBLL1*	1
rs1304218	7	49127785	49701285	0		N/A
rs17526000	10	96987000	97031000	1	*PDLIM1*	1
rs4842841	15	82278000	83256000	10	*SLC28A1*	0.45502052
rs13231420	7	49852785	50165785	2	*VWC2*	0.20894234

Supplementary Table 20. Enrichment of leptin-associated loci ($P<1x10^{-5}$) in the ChromHMM annotations for adipose and brain muscle tissues available from the Roadmap Epigenomics Project.

Cell line ID	Tissue	Cell Description	Active Promoter	Enhancer	Bivalent Enhancer	Bivalent Poised TSS	Flanking Active TSS	Bivalent Flank TSS/Enhancer	Genic Enhancer	Heterochromatin	Quiescent /Low	Repressed PolyComb	Strong Transcription	Transcription at gene 5' or 3'	Weak Repressed	Weak Transcription
			P value	P value	P value	P value	P value	P value	P value	P value	P value	P value	P value	P value	P value	P value
E023	Mesench	FAT.MSC.DR.ADIP	0.868	0.367	1	1	0.375	1	0.558	0.022	0.583	0.676	0.036	1	0.540	0.327
E025	Mesench	FAT.ADIP.DR.MSC	0.561	0.064	1	1	0.593	1	0.650	0.081	0.294	0.782	0.330	1	0.253	0.336
E063	Adipose	FAT.ADIP.NUC	0.718	0.072	1	1	1	1	0.163	0.360	0.809	0.713	0.142	1	0.598	0.013
E067	Brain	BRN.ANG.GYR	0.546	0.451	1	1	1	1	1	0.065	0.568	1	0.073	1	0.746	0.151
E068	Brain	BRN.ANT.CAUD	0.620	0.567	1	1	0.355	1	1	0.186	0.889	1	0.152	1	0.462	0.391
E069	Brain	BRN.CING.GYR	0.850	0.313	1	1	0.299	1	1	0.050	0.929	0.513	0.289	1	0.727	0.313
E070	Brain	BRN.GRM.MTRX	0.755	0.272	1	1	0.268	1	1	0.136	0.960	0.634	0.353	1	0.629	0.222
E071	Brain	BRN.HIPP.MID	0.850	0.411	1	1	0.395	1	1	0.014	0.605	0.483	0.356	1	0.503	0.350
E072	Brain	BRN.INF.TMP	0.563	0.735	1	1	0.702	1	1	0.030	0.475	1	0.343	1	0.378	0.533
E073	Brain	BRN.DL.PRFRNTL.CRTX	0.248	0.586	1	1	1	1	1	0.309	0.514	0.349	0.255	1	0.543	0.484
E074	Brain	BRN.SUB.NIG	1	0.352	1	1	0.659	1	0.062	0.004	0.600	1	0.242	1	0.452	0.147
E081	Brain	BRN.FET.M	1	0.456	1	1	0.564	1	1	0.046	0.949	0.501	0.249	1	0.064	0.187
E082	Brain	BRN.FET.F	0.187	0.584	1	1	0.646	1	1	0.043	0.956	0.712	0.410	1	0.112	0.126
E125	Brain	BRN.NHA	1	0.703	1	1	0.800	1	0.373	0.523	0.903	0.408	0.313	1	0.250	0.281

Supplementary Table 21. Association of established susceptibility loci for BMI and extreme obesity with BMI-unadjusted levels of leptin.

SNP	Chr	Position (bp, hg18)	Nearest gene	Effect allele[a]	Other allele	EAF (%)	Per-allele effect on leptin[b]	*P*-value	N
**BMI associated loci**									
rs977747	1	47,457,264	*TAL1*	T	G	38%	0.005	4.72E-01	32,086
rs657452	1	49,362,434	*AGBL4*	A	G	39%	0.010	1.54E-01	29,453
rs11583200	1	50,332,407	*ELAVL4*	C	T	39%	0.006	3.65E-01	32,153
rs3101336	1	72,523,773	*NEGR1*	C	T	63%	0.009	1.86E-01	32,154
rs12566985	1	74,774,781	*FPGT-TNNI3K*	G	A	46%	0.010	1.05E-01	32,055
rs12401738	1	78,219,349	*FUBP1*	A	G	33%	0.012	8.34E-02	32,119
rs11165643	1	96,696,685	*PTBP2*	T	C	58%	0.014	2.63E-02	32,136
rs17024393	1	109,956,211	*GNAT2*	C	T	4%	0.039	3.41E-02	30,500
rs543874	1	176,156,103	*SEC16B*	G	A	18%	0.009	2.89E-01	32,081
rs2820292	1	200,050,910	*NAV1*	C	A	54%	0.005	4.58E-01	29,470
rs13021737	2	622,348	*TMEM18*	G	A	82%	0.028	1.13E-03	29,472
rs10182181	2	25,003,800	*ADCY3*	G	A	46%	0.014	3.11E-02	32,141
rs11126666	2	26,782,315	*KCNK3*	A	G	29%	-0.001	8.75E-01	32,093
rs1016287	2	59,159,129	*FLJ30838*	T	C	28%	0.008	2.85E-01	29,472
rs11688816	2	62,906,552	*EHBP1*	G	A	51%	0.008	1.88E-01	32,159
rs2121279	2	142,759,755	*LRP1B*	T	C	16%	0.004	6.94E-01	32,083
rs1460676	2	164,275,935	*FIGN*	C	T	18%	0.012	1.42E-01	32,143
rs1528435	2	181,259,207	*UBE2E3*	T	C	64%	0.003	6.60E-01	32,093
rs17203016	2	207,963,763	*CREB1*	G	A	19%	0.006	5.02E-01	32,113
rs7599312	2	213,121,476	*ERBB4*	G	A	73%	0.003	6.79E-01	32,104
rs492400	2	219,057,996	*USP37*	C	T	43%	0.006	3.17E-01	32,030
rs2176040	2	226,801,046	*LOC646736*	A	G	36%	0.022	6.37E-04	32,157
rs6804842	3	25,081,441	*RARB*	G	A	56%	0.008	2.63E-01	29,468
rs2365389	3	61,211,502	*FHIT*	C	T	58%	0.021	1.56E-03	29,472
rs3849570	3	81,874,802	*GBE1*	A	C	35%	0.004	5.08E-01	32,127
rs13078960	3	85,890,280	*CADM2*	G	T	20%	-0.002	7.98E-01	32,137
rs16851483	3	142,758,126	*RASA2*	T	G	7%	0.022	8.53E-02	32,155
rs1516725	3	187,306,698	*ETV5*	C	T	87%	0.000	9.83E-01	32,129
rs10938397	4	44,877,284	*GNPDA2*	G	A	44%	0.020	4.11E-03	29,471
rs17001654	4	77,348,592	*SCARB2*	G	C	16%	0.002	8.30E-01	29,467
rs13107325	4	103,407,732	*SLC39A8*	T	C	8%	0.028	4.47E-02	31,320
rs11727676	4	145,878,514	*HHIP*	T	C	92%	0.003	8.51E-01	27,848
rs2112347	5	75,050,998	*POC5*	T	G	62%	0.013	4.39E-02	32,050
rs7715256	5	153,518,086	*GALNT10*	G	T	42%	0.000	9.49E-01	32,109
rs205262	6	34,671,142	*C6orf106*	G	A	28%	0.001	9.43E-01	32,160
rs2033529	6	40,456,631	*TDRG1*	G	A	29%	0.012	1.20E-01	29,470
rs2207139	6	50,953,449	*TFAP2B*	G	A	17%	0.020	1.42E-02	32,132

SNP	Chr	Position (bp, hg18)	Nearest gene	Effect allele[a]	Other allele	EAF (%)	Per-allele effect on leptin[b]	*P* -value	N
rs9400239	6	109,084,356	*FOXO3*	C	T	67%	0.019	8.22E-03	29,472
rs9374842	6	120,227,364	*LOC285762*	T	C	75%	0.013	7.72E-02	32,110
rs13201877	6	137,717,234	*IFNGR1*	G	A	14%	0.017	6.65E-02	32,155
rs13191362	6	162,953,340	*PARK2*	A	G	88%	0.010	3.22E-01	32,097
rs1167827	7	75,001,105	*HIP1*	G	A	53%	0.008	2.48E-01	30,759
rs2245368	7	76,446,079	*PMS2L11*	C	T	18%	0.001	9.49E-01	25,507
rs9641123	7	93,035,668	*CALCR*	C	G	42%	0.002	8.17E-01	29,469
rs6465468	7	95,007,450	*ASB4*	T	G	30%	0.016	4.02E-02	29,468
rs17405819	8	76,969,139	*HNF4G*	T	C	70%	0.009	2.20E-01	29,472
rs16907751	8	81,538,012	*ZBTB10*	C	T	95%	-0.004	8.34E-01	29,472
rs2033732	8	85,242,264	*RALYL*	C	T	75%	0.007	3.83E-01	29,472
rs4740619	9	15,624,326	*C9orf93*	T	C	54%	0.006	3.83E-01	32,135
rs10968576	9	28,404,339	*LINGO2*	G	A	32%	0.013	4.88E-02	32,156
rs6477694	9	110,972,163	*EPB41L4B*	C	T	36%	0.006	3.98E-01	32,154
rs1928295	9	119,418,304	*TLR4*	T	C	54%	0.007	2.60E-01	32,121
rs10733682	9	128,500,735	*LMX1B*	A	G	48%	0.005	4.51E-01	32,153
rs7899106	10	87,400,884	*GRID1*	G	A	5%	0.013	3.88E-01	32,118
rs17094222	10	102,385,430	*HIF1AN*	C	T	21%	0.018	3.28E-02	29,010
rs11191560	10	104,859,028	*NT5C2*	C	T	9%	-0.001	9.20E-01	32,121
rs7903146	10	114,748,339	*TCF7L2*	C	T	71%	0.010	1.57E-01	32,160
rs4256980	11	8,630,515	*TRIM66*	G	C	64%	0.007	2.68E-01	32,153
rs11030104	11	27,641,093	*BDNF*	A	G	79%	0.023	2.59E-03	32,127
rs2176598	11	43,820,854	*HSD17B12*	T	C	26%	0.016	2.98E-02	29,472
rs3817334	11	47,607,569	*MTCH2*	T	C	40%	0.021	9.25E-04	32,084
rs12286929	11	114,527,614	*CADM1*	G	A	53%	0.013	5.06E-02	29,464
rs7138803	12	48,533,735	*BCDIN3D*	A	G	38%	0.011	1.03E-01	32,077
rs11057405	12	121,347,850	*CLIP1*	G	A	91%	0.014	2.48E-01	30,525
rs12016871	13	26,915,782	*MTIF3*	T	C	20%	0.003	7.01E-01	32,044
rs12429545	13	53,000,207	*OLFM4*	A	G	14%	0.020	4.12E-02	32,148
rs9540493	13	65,103,705	*MIR548X2*	A	G	44%	0.002	7.56E-01	26,915
rs1441264	13	78,478,920	*MIR548A2*	A	G	62%	0.004	5.61E-01	31,317
rs10132280	14	24,998,019	*STXBP6*	C	A	67%	-0.003	7.15E-01	29,470
rs12885454	14	28,806,589	*PRKD1*	C	A	65%	0.009	1.71E-01	31,861
rs11847697	14	29,584,863	*PRKD1*	T	C	4%	0.039	2.48E-02	32,062
rs7141420	14	78,969,207	*NRXN3*	T	C	54%	0.007	2.73E-01	32,058
rs3736485	15	49,535,902	*DMXL2*	A	G	46%	0.007	3.03E-01	29,468
rs16951275	15	65,864,222	*MAP2K5*	T	C	79%	0.021	7.47E-03	32,154
rs7164727	15	70,881,044	*LOC100287559*	T	C	68%	0.009	1.74E-01	32,082

SNP	Chr	Position (bp, hg18)	Nearest gene	Effect allele[a]	Other allele	EAF (%)	Per-allele effect on leptin[b]	*P*-value	N
rs12446632	16	19,842,890	*GPRC5B*	G	A	87%	0.027	4.09E-03	32,157
rs2650492	16	28,240,912	*SBK1*	A	G	30%	0.022	3.94E-03	32,156
rs3888190	16	28,796,987	*ATP2A1*	A	C	39%	0.027	3.02E-05	32,157
rs4787491	16	29,922,838	*INO80E*	G	A	49%	0.015	4.69E-02	22,925
rs9925964	16	31,037,396	*KAT8*	A	G	61%	0.015	2.84E-02	29,471
rs2080454	16	47,620,091	*CBLN1*	C	A	40%	0.003	6.23E-01	32,089
rs1558902	16	52,361,075	*FTO*	A	T	42%	0.033	2.32E-07	32,041
rs9914578	17	1,951,886	*SMG6*	G	C	21%	-0.008	2.81E-01	32,145
rs1000940	17	5,223,976	*RABEP1*	G	A	32%	0.015	2.88E-02	32,157
rs12940622	17	76,230,166	*RPTOR*	G	A	58%	0.013	4.72E-02	32,149
rs1808579	18	19,358,886	*C18orf8*	C	T	54%	0.004	5.12E-01	32,155
rs7239883	18	38,401,669	*LOC284260*	G	A	40%	0.014	2.87E-02	32,002
rs7243357	18	55,034,299	*GRP*	T	G	81%	0.006	4.73E-01	29,472
rs6567160	18	55,980,115	*MC4R*	C	T	23%	0.027	4.21E-04	32,057
rs17724992	19	18,315,825	*PGPEP1*	A	G	75%	-0.004	6.54E-01	28,856
rs29941	19	39,001,372	*KCTD15*	G	A	67%	0.009	1.68E-01	32,160
rs2075650	19	50,087,459	*TOMM40*	A	G	85%	0.025	1.55E-02	28,973
rs2287019	19	50,894,012	*QPCTL*	C	T	80%	0.014	1.32E-01	28,323
rs3810291	19	52,260,843	*ZC3H4*	A	G	66%	0.008	2.75E-01	29,467
rs6091540	20	50,521,269	*ZFP64*	C	T	74%	0.002	7.69E-01	29,472
rs2836754	21	39,213,610	*ETS2*	C	T	59%	0.007	3.10E-01	29,468
**Extreme obesity associated loci**									
rs11208659	1	65,751,868	*LEPR*	C	T	9%	0.026	2.18E-02	32,138
rs17381664	1	77,820,919	*ZZZ3*	C	T	37%	0.012	6.45E-02	32,113
rs17150703	8	9,783,208	*MSRA*	A	G	12%	0.006	5.51E-01	32,051
rs10508503	10	16,339,957	*PTER*	C	T	91%	0.029	2.37E-02	29,469
rs564343	11	65,651,742	*PACS1*	A	G	41%	0.026	5.49E-01	840
rs11109072	12	96,425,401	*RMST*	A	C	6%	-0.020	1.85E-01	29,472
rs7989336	13	95,815,549	*HS6ST3*	A	G	51%	0.006	5.27E-03	32,077
rs1957894	14	60,977,864	*PRKCH*	T	G	9%	-0.028	1.50E-02	32,129
rs2531995	16	3,953,468	*ADCY9*	T	C	60%	0.015	3.57E-02	32,150
rs1424233	16	78,240,252	*MAF*	A	G	47%	0.000	9.87E-01	32,156
rs1805081	18	19,394,430	*NPC1*	A	G	59%	0.002	8.15E-01	32,155
rs13041126	20	50,526,403	*MRPS33P4*	T	C	73%	0.000	9.54E-01	32,145

Chr, chromosome; EAF, Effect allele frequency.

[a] The effect allele is the allele associated with increased BMI and extreme obesity risk.

[b] Effects sizes are expressed for logarighmically transformed BMI-unadjusted leptin levels

Supplementary Table 22. Association of established susceptibility loci for WHRadjBMI with BMI-adjusted levels of leptin.

SNP	Chr	Position (bp, hg18)	Nearest gene	Effect allele[a]	Other allele	EAF (%)	Men and women			Men			Women		
							Per-allele effect on BMIadj leptin[b]	*P*-value	N	Per-allele effect on BMIadj leptin[b]	*P*-value	N	Per-allele effect on BMIadj leptin[b]	*P*-value	N
rs2645294	1	119,376,110	*TBX15-WARS2*	t	c	58%	0.0001	9.84E-01	31,775	0.0071	3.93E-01	13,177	-0.0034	5.81E-01	18,598
rs905938	1	153,258,013	*DCST2*	t	c	74%	-0.0089	1.41E-01	31,247	0.0015	8.84E-01	12,894	-0.0148	4.53E-02	18,353
rs10919388	1	168,639,127	*GORAB*	c	a	72%	0.0010	8.46E-01	31,813	0.0013	8.87E-01	13,177	0.0008	9.09E-01	18,636
rs714515	1	170,619,613	*DNM3-PIGC*	g	a	43%	0.0000	9.99E-01	31,785	0.0063	4.38E-01	13,177	-0.0045	4.54E-01	18,608
rs2820443	1	217,820,132	*LYPLAL1*	t	c	72%	0.0047	3.85E-01	31,776	0.0064	4.76E-01	13,177	0.0025	7.06E-01	18,599
rs1385167	2	66,054,152	*MEIS1*	g	a	15%	0.0030	6.66E-01	31,702	0.0075	5.16E-01	13,176	0.0011	8.98E-01	18,526
rs10195252	2	165,221,337	*GRB14-COBLL1*	t	c	60%	-0.0187	1.76E-04	31,810	-0.0142	9.17E-02	13,175	-0.0217	4.41E-04	18,635
rs1569135	2	187,823,643	*CALCRL*	a	g	53%	-0.0004	9.30E-01	31,814	0.0006	9.41E-01	13,176	-0.0015	7.97E-01	18,638
rs17819328	3	12,464,342	*PPARG*	g	t	44%	-0.0046	3.81E-01	29,251	-0.0030	7.21E-01	13,174	-0.0052	4.43E-01	16,077
rs2276824	3	52,612,526	*PBRM1c*	c	g	43%	0.0021	6.94E-01	29,256	0.0009	9.20E-01	13,177	0.0043	5.36E-01	16,080
rs2371767	3	64,693,298	*ADAMTS9*	g	c	71%	0.0040	4.89E-01	29,257	0.0077	4.04E-01	13,177	0.0025	7.31E-01	16,080
rs10804591	3	130,816,923	*PLXND1*	a	c	79%	-0.0095	1.07E-01	31,811	-0.0097	3.35E-01	13,174	-0.0084	2.46E-01	18,637
rs17451107	3	158,280,303	*LEKR1*	t	c	61%	0.0226	5.11E-05	29,257	0.0128	1.52E-01	13,177	0.0279	8.20E-05	16,080
rs3805389	4	56,177,507	*NMU*	a	g	28%	0.0025	6.42E-01	31,815	-0.0132	1.56E-01	13,177	0.0099	1.44E-01	18,639
rs9991328	4	89,932,144	*FAM13A*	t	c	49%	-0.0064	1.88E-01	31,812	-0.0145	7.83E-02	13,176	-0.0017	7.79E-01	18,636
rs303084	4	124,286,398	*SPATA5-FGF2*	a	g	80%	0.0013	8.28E-01	31,813	0.0223	2.86E-02	13,175	-0.0088	2.44E-01	18,638
rs9687846	5	55,897,651	*MAP3K1*	a	g	19%	-0.0117	7.78E-02	29,256	-0.0102	3.35E-01	13,177	-0.0127	1.36E-01	16,079
rs1045241	5	118,757,185	*TNFAIP3-HSD17B*	c	t	71%	-0.0043	4.51E-01	29,249	-0.0029	7.51E-01	13,173	-0.0052	4.81E-01	16,076
rs7705502	5	173,253,421	*CPEB4*	a	g	33%	-0.0105	4.36E-02	31,812	-0.0252	4.08E-03	13,177	-0.0021	7.38E-01	18,635
rs6556301	5	176,460,183	*FGFR4*	t	g	35%	0.0043	4.28E-01	31,808	0.0085	3.57E-01	13,173	0.0023	7.33E-01	18,635
rs1294410	6	6,683,751	*LY86*	t	c	63%	-0.0135	1.18E-02	29,253	-0.0130	1.30E-01	13,175	-0.0123	7.10E-02	16,078
rs7759742	6	32,489,714	*BTNL2*	a	t	51%	-0.0101	5.42E-02	29,257	-0.0082	3.25E-01	13,177	-0.0121	6.90E-02	16,080
rs1776897	6	34,302,989	*HMGA1*	g	t	8%	-0.0082	4.61E-01	23,693	-0.0333	9.96E-02	9,339	0.0006	9.65E-01	14,355
rs1358980	6	43,872,529	*VEGFA*	t	c	47%	0.0068	2.30E-01	29,255	-0.0009	9.19E-01	13,176	0.0115	1.06E-01	16,079
rs1936805	6	127,493,809	*RSPO3*	t	c	51%	-0.0148	3.90E-03	29,235	-0.0065	4.25E-01	13,163	-0.0200	2.33E-03	16,072

SNP	Chr	Position (bp, hg18)	Nearest gene	Effect allele[a]	Other allele	EAF (%)	Men and women			Men			Women		
							Per-allele effect on BMIadj leptin[b]	*P* -value	N	Per-allele effect on BMIadj leptin[b]	*P* -value	N	Per-allele effect on BMIadj leptin[b]	*P* -value	N
rs10245353	7	25,825,139	*NFE2L3*	a	c	21%	0.0093	1.45E-01	29,254	0.0105	3.03E-01	13,176	0.0089	2.80E-01	16,077
rs1534696	7	26,363,764	*SNX10*	c	a	44%	-0.0032	5.42E-01	29,219	0.0019	8.26E-01	13,163	-0.0069	3.06E-01	16,056
rs7801581	7	27,190,296	*HOXA11*	t	c	25%	0.0174	7.06E-03	29,254	0.0201	5.23E-02	13,177	0.0151	6.76E-02	16,078
rs7830933	8	23,659,269	*NKX2-6*	a	g	76%	0.0017	7.66E-01	31,772	0.0007	9.45E-01	13,177	-0.0032	6.48E-01	18,595
rs12679556	8	72,676,782	*MSC*	g	t	25%	-0.0093	9.63E-02	31,790	0.0005	9.58E-01	13,174	-0.0148	3.37E-02	18,616
rs10991437	9	106,775,741	*ABCA1*	a	c	11%	0.0031	6.95E-01	30,920	0.0164	2.33E-01	12,378	-0.0035	7.21E-01	18,542
rs7917772	10	104,477,433	*SFXN2*	a	g	62%	0.0016	7.46E-01	31,753	0.0086	3.12E-01	13,173	-0.0013	8.34E-01	18,580
rs11231693	11	63,619,188	*MACROD1-VEGFB*	a	g	6%	0.0106	3.42E-01	28,301	0.0123	4.89E-01	12,702	0.0095	5.05E-01	15,599
rs10842707	12	26,362,631	*ITPR2-SSPN*	t	c	23%	-0.0023	7.09E-01	29,257	0.0008	9.37E-01	13,177	-0.0043	5.85E-01	16,080
rs1443512	12	52,628,951	*HOXC13*	a	c	24%	-0.0022	6.92E-01	31,814	0.0065	4.88E-01	13,177	-0.0084	2.32E-01	18,637
rs4765219	12	123,006,063	*CCDC92*	c	a	67%	-0.0041	4.25E-01	31,794	-0.0106	2.27E-01	13,176	-0.0014	8.23E-01	18,618
rs8042543	15	29,495,555	*KLF13*	c	t	78%	0.0178	9.48E-03	29,253	0.0271	1.41E-02	13,175	0.0117	1.78E-01	16,077
rs8030605	15	54,291,890	*RFX7*	a	g	15%	0.0050	5.09E-01	31,791	0.0002	9.85E-01	13,173	0.0073	4.28E-01	18,618
rs1440372	15	64,820,205	*SMAD6*	c	t	72%	0.0055	3.21E-01	31,782	0.0139	1.37E-01	13,176	0.0006	9.29E-01	18,606
rs2925979	16	80,092,291	*CMIP*	t	c	30%	0.0019	7.25E-01	31,807	-0.0072	4.32E-01	13,174	0.0057	3.95E-01	18,633
rs4646404	17	17,360,924	*PEMT*	g	a	66%	-0.0080	1.74E-01	29,252	-0.0081	3.93E-01	13,175	-0.0078	2.97E-01	16,078
rs8066985	17	65,964,940	*KCNJ2*	a	g	50%	-0.0046	3.40E-01	31,734	-0.0032	7.01E-01	13,177	-0.0051	3.91E-01	18,557
rs12454712	18	58,996,864	*BCL2*	t	c	60%	-0.0008	9.16E-01	25,401	-0.0208	7.87E-02	11,339	0.0138	1.49E-01	14,062
rs12608504	19	18,250,135	*JUND*	a	g	35%	-0.0113	2.64E-02	31,810	-0.0123	1.49E-01	13,174	-0.0105	9.66E-02	18,636
rs4081724	19	38,516,786	*CEBPA*	g	a	86%	-0.0105	1.90E-01	29,256	-0.0084	5.11E-01	13,177	-0.0111	2.81E-01	16,079
rs979012	20	6,571,374	*BMP2*	t	c	34%	0.0167	1.05E-03	31,698	0.0186	3.04E-02	13,175	0.0153	1.53E-02	18,523
rs224333	20	33,487,376	*GDF5*	g	a	62%	0.0073	1.85E-01	29,257	0.0048	5.83E-01	13,177	0.0101	1.54E-01	16,080
rs6090583	20	44,992,238	*EYA2*	a	g	49%	-0.0177	5.27E-04	29,254	-0.0201	1.36E-02	13,174	-0.0154	1.85E-02	16,080
rs2294239	22	27,779,477	*ZNRF3-KREMEN1*	a	g	59%	-0.0061	2.22E-01	31,808	-0.0058	5.01E-01	13,173	-0.0068	2.73E-01	18,635

Chr, chromosome; EAF, Effect allele frequency.

[a] The effect allele is the allele associated with increased WHRadjBMI

[b] Effects sizes are expressed for logarighmically transformed leptin levels

Supplementary Table 23. Study-specific descriptive statistics of the participating studies.

Study	Men													Correlation		Women													Correlation	
		Age, yrs		BMI, kg/m2		Height, cm		Weight, kg		Body fat percentage (%)		Leptin, ng/ml		Leptin vs. BMI	Leptin vs. Fat%		Age, yrs		BMI, kg/m2		Height, cm		Weight, kg		Body fat percentage (%)		Leptin, ng/ml		Leptin vs. BMI	Leptin vs. Fat%
	n*	mean	SD	mean	SD	mean	SD	mean	SD	mean	SD	mean	SD	r	r	n*	mean	SD	mean	SD	mean	SD	mean	SD	mean	SD	mean	SD	r	r
**BLSA**	250	72.7	13.0	27.5	4.3	175.0	8.2	81.3	15.0	-	-	14.9	15.6	0.63	-	137	68.0	14.1	28.0	5.3	162.6	7.1	67.2	13.3	-	-	38.3	4.7	0.70	-
**BWHHS**	-	-	-	-	-	-	-	-	-	-	-	-	-	-	-	500	70.2	5.4	27.7	4.6	158.6	6.1	69.5	12.0	-	-	27.2	19.3	0.73	-
**COLAUS**	2250	53.5	10.8	27.0	3.8	172.0	7.3	82.7	12.8	24.5	5.8	8.7	7.1	0.55	0.49	2676	53.7	10.7	25.0	4.6	162.7	6.7	66.0	12.1	34.3	8.0	17.1	11.9	0.69	0.54
**Ely Study**	741	61.5	9.1	27.4	4.0	174.2	6.7	83.1	13.3	26.6	5.2	9.6	8.4	0.65	0.55	854	60.8	9.3	27.3	5.4	161.4	6.1	71.1	14.3	39.9	6.5	33.7	26.9	0.78	0.66
**ERF**	839	49.6	15.7	27.3	4.2	174.8	7.2	83.3	13.7	26.8	7.1	24.6	29.5	0.64	0.75	1112	50.7	16.4	26.5	5.0	161.9	6.5	69.5	13.4	38.8	7.6	74.9	65.1	0.68	0.73
**FamHS**	737	52.5	13.9	27.8	5.0	177.0	7.0	87.5	17.3	26.9	8.2	8.5	7.0	0.80	0.62	768	52.8	13.1	28.1	6.9	160.0	6.5	74.5	18.6	38.1	8.4	23.4	17.9	0.83	0.72
**Fenland**	610	44.5	7.3	27.6	3.9	177.3	6.5	86.7	13.9	28.0	6.7	7.6	7.3	0.68	0.73	785	45.4	7.2	26.7	5.5	163.7	6.2	71.5	15.2	37.6	7.6	23.9	20.4	0.79	0.76
**FinRisk97**	245	52.2	13.1	26.9	3.9	176.0	7.0	83.3	13.1	-	-	6.4	5.5	0.67	-	275	59.1	10.6	26.3	4.7	162.0	7.0	68.3	12.4	-	-	19.6	14.9	0.76	-
**GARP**	38	60.1	7.9	27.4	3.2	176.1	7.0	84.9	10.6	-	-	5.4	6.7	0.11	-	202	58.4	7.0	27.4	5.3	165.0	6.5	75.3	15.3	-	-	9.2	8.6	0.39	-
**GEMS cases**	521	49.4	8.5	28.6	3.2	177.4	6.9	90.2	12.1	-	-	11.6	8.7	0.51	-	282	49.6	9.9	28.3	3.7	162.6	5.8	74.9	10.7			24.5	12.2	0.36	-
**GEMS controls**	491	55.5	9.1	28.0	3.4	177.4	6.6	88.1	12.8	-	-	10.1	9.4	0.47	-	329	54.2	8.9	27.9	2.9	162.5	6.1	73.7	9.7			21.5	12.6	0.26	-
**GLACIER**	601	51.1	8.0	26.1	3.6	176.4	6.8	81.3	11.5	-	-	4.6	3.4	0.70	-	391	51.0	8.0	25.5	4.1	164.0	5.6	68.7	11.6	-	-	12.9	8.2	0.69	-
**GOOD**	938	18.9	0.6	22.4	3.2	182.0	7.0	73.9	11.6	17.1	7.3	7.7	8.6	0.45	0.62	-	-	-	-	-	-	-	-	-	-	-	-	-	-	-
**GOYA Male**	270	49.7	6.2	26.1	3.8	178.6	6.0	83.5	13.2	21.7	8.1	4.8	3.5	0.73	0.84	-	-	-	-	-	-	-	-	-	-	-	-	-	-	-
**H2000CASES**	497	49.8	10.5	29.4	3.6	175.0	7.0	91.1	12.2	25.3	5.4	11.5	10.0	0.50	0.63	494	53.1	11.4	29.7	4.8	161.0	7.0	76.5	13.5	36.2	6.1	32.9	19.3	0.65	0.65
**H2000CTRLS**	488	48.3	10.1	25.3	3.0	175.0	7.0	78.0	10.1	19.6	5.1	5.9	5.8	0.53	0.65	544	51.3	11.4	24.9	3.9	161.0	7.0	65.2	10.2	29.6	6.5	19.1	15.0	0.67	0.73
**HBCS**	696	61.4	2.8	27.6	4.3	177.0	6.0	86.3	14.4	23.8	6.0	13.7	16.6	0.44	0.48	909	61.6	3.0	27.8	5.1	163.0	6.0	74.0	13.8	34.0	6.8	24.4	20.2	0.57	0.59
**Health ABC**	291	73.8	2.9	29.1	3.6	173.5	6.9	87.8	12.9	32.6	4.5	13.3	8.6	0.41	0.49	414	73.4	2.7	28.2	4.1	159.6	5.8	71.8	11.0	42.8	4.1	27.0	13.8	0.41	0.55
**HERITAGE**	242	36.7	15.0	26.7	4.9	178.0	6.0	84.7	16.3	22.9	9.0	7.1	6.0	0.80	0.88	244	35.1	14.1	25.0	4.8	163.0	6.0	66.9	13.6	29.9	9.9	18.3	13.5	0.78	0.77
**InCHIANTI**	418	66.1	15.8	27.1	3.4	167.0	8.0	75.8	12.0	-	-	6.6	7.8	0.59	-	542	68.1	15.4	27.2	4.6	154.3	7.3	64.8	11.2	-	-	17.4	15.3	0.56	-
**Inter99**	2869	46.5	7.8	26.7	4.0	178.8	6.9	85.5	14.0	-	-	4.6	5.2	0.61	-	2961	45.8	8.0	25.8	5.0	166.0	6.3	71.0	14.4	-	-	15.1	16.2	0.66	-
**KORA-F3**	797	62.9	10.1	28.2	3.8	-	-	-	-	29.0	5.2	9.5	9.4	0.70	0.76	820	62.1	10.1	28.0	5.1	-	-	-	-	38.4	6.8	30.7	26.1	0.78	0.79
**KORA-F4**	428	68.9	4.5	28.5	3.9	-	-	-	-	-	-	10.7	12.2	0.66	-	412	68.7	4.2	28.9	5.0	-	-	-	-	-	-	33.5	27.1	0.78	-
**LLS**	1033	59.9	6.7	25.7	3.0	-	-	-	-	-	-	10.2	12.1	0.50	-	1226	58.7	6.7	25.1	3.9	-	-	-	-	-	-	29.1	24.8	0.67	-
**LURIC**	312	54.7	12.7	27.4	3.8	176.7	7.0	85.4	12.9	-	-	9.2	7.8	0.60	-	287	62.3	10.8	27.4	4.8	162.6	6.6	72.4	13.6	-	-	25.9	16.5	0.64	-
**MESA**	397	62.6	10.2	28.2	4.0	176.8	6.9	88.1	14.2	-	-	10.4	10.6	0.51	-	363	62.9	9.2	27.4	5.7	162.8	6.5	72.9	16.2	-	-	27.0	23.0	0.68	-

Study	Men															Women														
		Age, yrs		BMI, kg/m2		Height, cm		Weight, kg		Body fat percentage (%)		Leptin, ng/ml		Correlation Leptin vs. BMI	Correlation Leptin vs. Fat%		Age, yrs		BMI, kg/m2		Height, cm		Weight, kg		Body fat percentage (%)		Leptin, ng/ml		Correlation Leptin vs. BMI	Correlation Leptin vs. Fat%
	n*	mean	SD	mean	SD	mean	SD	mean	SD	mean	SD	mean	SD	r	r	n*	mean	SD	mean	SD	mean	SD	mean	SD	mean	SD	mean	SD	r	r
MICROS	470	45.5	15.9	26.1	3.9	173.0	7.0	78.4	13.0	-	-	2.0	2.6	0.57	-	627	45.9	16.5	25.4	5.3	161.0	7.0	65.2	13.2	-	-	7.0	9.1	0.66	-
MrOS Sweden	919	75.3	3.2	26.2	3.5	176.0	6.0	80.9	12.1	22.7	4.8	22.7	21.1	0.63	0.77	-	-	-	-	-	-	-	-	-	-	-	-	-	-	-
NHS T2D	-	-	-	-	-	-	-	-	-	-	-	-	-	-	-	1793	56.7	6.9	28.4	5.9	163.6	9.1	74.8	19.5	-	-	28.1	19.5	0.74	-
NHS CGEM	-	-	-	-	-	-	-	-	-	-	-	-	-	-	-	336	58.6	6.5	25.2	4.7	164.5	6.1	67.1	15.1	-	-	22.0	16.1	0.71	-
PIVUS	474	70.1	0.2	27.0	3.7	175.9	6.5	83.6	13.0	-	-	8.0	5.7	0.70	-	474	70.3	0.2	27.1	4.9	162.2	5.6	71.3	13.1	-	-	19.4	5.7	0.73	-
PROSPER	2475	75.0	3.3	26.6	3.6	172.1	6.7	78.7	11.9	-	-	2.1	0.7	0.65	-	2673	75.7	3.4	27.1	4.7	158.8	6.7	68.3	12.7	-	-	3.1	0.8	0.70	-
QFS	254	43.7	16.2	27.6	5.9	172.6	6.8	82.2	18.4	23.1	8.6	9.1	7.8	0.68	0.71	329	43.8	17.1	28.0	8.1	159.6	6.8	71.1	20.6	32.4	10.0	25.7	18.9	0.73	0.74
RISC	156	44.7	8.3	26.0	3.5	178.6	7.3	83.0	12.4	21.5	7.2	5.5	5.6	0.68	0.70	157	45.8	7.9	25.2	4.5	164.9	6.7	68.6	13.2	32.8	8.1	20.9	16.6	0.82	0.70
Rotterdam	482	66.1	5.1	26.7	2.8	177.1	5.8	83.7	9.9	-	-	6.3	4.5	0.49	-	486	66.8	6.0	26.1	4.1	163.1	7.1	69.5	11.9	-	-	21.1	17.5	0.68	-
TwinsUK	-	-	-	-	-	-	-	-	-	-	-	-	-	-	-	2689	47.6	12.5	25.0	4.5	162.4	6.1	65.8	12.2	35.7	7.8	17.0	12.7	0.73	0.73
WGHS	-	-	-	-	-	-	-	-	-	-	-	-	-	-	-	808	54.6	7.4	25.8	5.0	164.0	6.0	69.4	14.5	-	-	23.0	17.1	0.70	-
YFS	941	31.7	5.0	25.8	4.1	180.0	7.0	83.4	15.0	16.1	6.2	5.5	4.3	0.72	0.81	1144	31.7	5.0	24.5	4.6	166.0	6.0	67.8	13.3	27.0	7.4	15.8	10.2	0.73	0.79

* number of the individuals included in the leptin analyses

Supplementary Table 24. Information on genotyping methods, quality control of SNPs, imputation, and statistical analysis of the participating studies.

Cohort	GENOTYPING						IMPUTATION			Association analyses software
	Platform	Genotype calling algorithm	Inclusion criteria			SNPs that met QC criteria	Imputation software	Inclusion criteria		
			MAF	Call rate*	p for HWE			MAF	Imputation quality**	
**BLSA**	Illumina 550K	Beadstudio	≥ 1%	≥ 99%	$> 10^{-4}$	498,838	MACH	≥1%	r2-hat ≥ 0.30	Merlinoffline
**BWHHS**	50K HumanCVD BeadChip	GenCall	> 0%	≥ 95%	$> 10^{-7}$	50,000	-	-	-	PLINK
**COLAUS**	Affymetrix GeneChip Human Mapping 500K	BRLMM	≥ 1%	≥ 70%	$> 10^{-7}$	390,631	IMPUTE	-	proper-info ≥ 0.40	In-house Matlab code
**Ely Study**	Illumina Metabochip	GenCall	> 0%	≥ 90%	$> 10^{-6}$	149,302	-	-	-	SAS
**ERF**	Illumina 300K and 370K; Affymetrix 250K	Beadstudio, blrmm	≥ 1%	≥ 95.5%	$> 10^{-6}$	650,197	MACH	≥1%	r2-hat ≥ 0.30	GenABEL/ProbABEL
**FamHS**	Illumina 550K, 610K, and 1M	Bead Studio	≥ 1%	≥ 98%	$> 10^{-6}$	499,558	MACH	-	r2-hat ≥ 0.30	lmekin R Package in
**Fenland**	Affymetrix SNP5.0	BRLMM	≥ 1%	≥ 90%	$> 10^{-6}$	362,055	IMPUTE	≥1%	proper-info ≥ 0.40	SNPtest
**FINRISK97**	Illumina Human 610-Quad	Illuminus	≥ 5%	≥ 95%	$> 10^{-6}$	554,988	MACH	-	r2-hat ≥ 0.30	ProbABEL
**GARP study**	Illumina Human660W	Genome studio	> 5%	> 98%	$> 10^{-4}$	510,865	IMPUTE	>5%	proper-info ≥ 0.40	QTASSOC In-house
**GEMS**	Affymetrix GeneChip Human Mapping 500K	BRLMM	≥1%	> 90%	$> 10^{-6}$	357,170	IMPUTE	≥1%	proper-info ≥ 0.40	ProbABEL
**GLACIER**	Illumina Metabochip	Illuminus	≥ 0%	≥ 95%	$> 10^{-6}$	189,315	-	-	-	SAS
**GOOD**	Illumina 610K	Bead Studio	≥ 1%	≥ 98%	$> 10^{-6}$	521,160	MACH	≥1%	r2-hat ≥ 0.30	PLINK
**GOYA Male**	Illumina Human 610K-Quad	GenCall	≥ 1%	≥ 95%	$> 10^{-7}$	545,349	MACH	≥1%	r2-hat ≥ 0.30	R
**H2000 CASES**	Illumina Human610-Quad	Illuminus	≥ 5%	≥ 95%	$> 10^{-6}$	555388	MACH	-	r2-hat ≥ 0.30	ProbABEL
**H2000 CTRLS**	Illumina Human610-Quad	Illuminus	≥ 5%	≥ 95%	$> 10^{-6}$	555,388	MACH	-	r2-hat ≥ 0.30	ProbABEL
**HBCS**	Illumina Human670-Quad	Illuminus	≥ 5%	≥ 95%	$> 10^{-6}$	533 491	MACH	-	r2-hat ≥ 0.30	ProbABEL
**Health ABC**	Illumina 1M	GenomeStudio	≥ 1%	≥ 95%	$> 10^{-5}$	899,818	MACH	≥1%	r2-hat ≥ 0.30	MACH2
**HERITAGE**	Illumina HumanCNV370-Quad v3.0	Illumina Genome Studio	≥ 1%	≥ 99%	$> 10^{-6}$	324,607	MACH	≥1%	r2-hat ≥ 0.30	ProbABEL
**InCHIANTI**	Illumina 550K	Beadstudio	≥ 1%	≥ 99%	$> 10^{-6}$	514,027	MACH	≥1%	r2-hat ≥ 0.30	Merlinoffline
**Inter99**	KASP Genotyping Assay (LGC Genomics, UK)	KlusterCaller	≥ 1%	≥ 95%	> 0.005	8	-	-	-	R
**Inter99**	Illumina MetaboChip	GenCall	≥ 1%	≥ 95%	$> 10^{-4}$	118,622	-	-	-	PLINK/R
**KORA-F3**	Affymetrix GeneChip Human Mapping 500K	BRLMM	≥ 1%	≥ 95%	$> 10^{-6}$	379,392	IMPUTE	-	proper-info ≥ 0.40	SNPtest
**KORA-F4**	Affymetrix 6.0 (1000K)	Birdseed2	-	-	-	909,622	IMPUTE	-	proper-info ≥ 0.40	SNPtest

Cohort	GENOTYPING Platform	Genotype calling algorithm	Inclusion criteria MAF	Call rate*	p for HWE	SNPs that met QC criteria	IMPUTATION Imputation software	Inclusion criteria MAF	Imputation quality**	Association analyses software
**LLS**	Illumina 660W-Quad, Illumina OmniExpress	Beadstudio	≥ 1%	≥ 95%	> 10^{-4}	516,712	IMPUTE	≥1%	proper-info ≥ 0.40	QTassoc
**LURIC**	Affymetrix 6.0	Birdseed v2	≥ 1%	≥ 98%	> 10^{-4}	686,195	MACH	-	r2-hat ≥ 0.30	QUICKTEST
**MESA**	Affymetrix 6.0	Birdseed v1.33	≥ 1%	≥ 95%	> 10^{-6}	881,666	IMPUTE2	≥1%	proper-info ≥ 0.40	SNPtest2
**MICROS**	Illumina CNV370, Illumina HumHap300v2	Bead Studio	>1%	≥ 98%	> 10^{-6}	292,917	MACH	≥1%	r2-hat ≥ 0.30	GeneABEL, ProbABEL
**MrOS Sweden**	Illumina 1M	Bead Studio	≥ 1%	≥ 97%	> 10-4	739,447	MINIMAC	≥1%	r2-hat ≥ 0.30	PLINK
**NHS T2D**	Affymetrix 6.0	Birdseed	>2%	≥ 95%	>10^{-4}	704,409	MACH	>2%	r2-hat ≥ 0.30	ProbABEL
**NHS CGEM**	Illumina HumanHap550	Illumina Beadstudio	>1%	≥ 90%	-	528,173	MACH	>2%	r2-hat ≥ 0.30	ProbABEL
**PIVUS**	Merged Metabochip and Omni Express	GenCall	≥ 1%	> 95%	> 10^{-6}	738,879	IMPUTE	≥1%	proper-info ≥ 0.40	SNPtest
**PROSPER**	Illumina 660K Quad	BeadStudio	-	> 97.5%	> 10^{-6}	557,192	MACH	≥1%	r2-hat ≥ 0.30	ProbABEL
**QFS**	Illumina 610-Quad chip	Bead Studio Genotyping	≥ 1%	≥ 95%	> 10^{-4}	543 173	MACH	≥1%	r2-hat ≥ 0.30	GWAF
**RISC**	Affymetrix 6.0	Birdseed	>1%	≥98%	> 10^{-4}	747,423	MACH	≥1%	r2_hat ≥ 0.30	MACH2QTL
**Rotterdam Study+A19**	Illumina HumanHap 550	Beadstudio Genecall	≥ 1%	≥ 97.5%	> 10^{-6}	512,349	MACH	-	r2_hat ≥ 0.30	MACH2QTL
**TwinsUK**	Illumina HumanHap300+ Illumina 610K Quad	Illuminus	≥ 1%	≥ 97%	> 10^{-6}	303,940 (Hap300)	IMPUTE	≥1%	proper-info ≥ 0.40	GenABEL
**WGHS**	Illumina HumanHap 300 DuoPlus	Illumina BeadStudio 3.3	≥ 1%	≥ 90%	> 10^{-6}	339,596	MACH	≥1%	r2-hat ≥ 0.30	SAS
**YFS**	Illumina Human 670-Quad	Illuminus	≥ 5%	≥ 95%	> 10^{-6}	546,677	MACH	-	r2-hat ≥ 0.30	ProbABEL

* Call rate to exclude SNPs for which less than a certain percentage of individuals were successfully genotyped

** Imputation quality filter used during the meta-analysis (proper_info ≥ 0.40 for IMPUTE, r2-hat ≥ 0.30 for MACH, or INFO ≥ 0.8 for PLINK)

Supplementary Table 25. Primers used for qPCR.

Gene	Forward primer	Reverse primer
**Housekeeping genes**		
Actb ex2-3	CGGGCTGTATTCCCCTCCAT	GGGCCTCGTCACCCACATAG
Rplp0 ex3-4	ACCTCCTTCTTCCAGGCTTTGG	CGAAGGAGAAGGGGGAGATGTT
Gapdh ex2-3	CTGGAGAAACCTGCCAAGTATGATG	GAGACAACCTGGTCCTCAGTGTAGC
Ppia ex2-3	CTTCGAGCTGTTTGCAGACAAAGTT	GGAGGAACCCTTATAGCCAAATCCT
***Lep* /*Fto* loci**		
Lep ex1-2	CGAGGAATCGTTCTGCAAATCC	GCCAGGTTAAGTGCAGCTATCACA
Fto ex1-2	GGCGGCTTTAGTAGCAGCAT	CCAAGTGTCTTCAAGCTCCTC
Rpgrip1l ex4-5	CCAAACAGCAGCTCCAAGTCCAGGG	GAGCGTGGGTTGTACAGTTTCTGCTTC
***Gckr* locus**		
Ucn ex1-2	TTGCACTGGGCAGACACTCC	AGCTGTGCCAAGAGCAGCAA
Ppm1g ex2-3	CCATGGAGGATGCTCACAACTG	TTTGGCACAGTACAAGGCAACC
Fndc4 ex3-4	GGGACGTTCCAGAAGGCAAC	AGGCCCTGGTGGTGGTGTT
Zfp512 ex2-3	GCAGAGGAGCACCAGGATCG	CGAGCCACTCAGGGAGTCCT
Mpv17 ex3-4	GGGGTCTCCAGCAACACCAG	GGTGCCCGGGATTAAGTGGT
Nrbp1 ex3-4	CCTGGCCATGGATACAGAGGA	TCAAACACTGCACGGACCTT
Gtf3c2 ex2-3	GGAAAGCCTCACCAGGTTGG	CGGAAGGCTTTGAGGCCCTA
Ift172 ex13-14	CATCAGCGTCCGTATCAATG	CCAATGTTGTAGCCACCAATC
Ccdc121 ex1-1	AGGCGCACTTCCAGTTCCTG	ATCTGCCCCAGCTCCATCAG
Eif2b4 ex2-3	AGGAAAAGGGGGCAGACCAA	CCTCCCAGCTGACTACCAGGTC
Gckr ex1-2	GCCAAGCACCAAGCGGTATC	CGGGTCAGTGGGTTGGACTT
Gpn1 ex2-3	CTGTGCATGAAGTCCCCTTT	GTTTGGCCCAAGTCCATATT
Krtcap3 ex2-3	TCATTTCTGTGGGTTCGGGACT	CAGTCGTGGGCGAAGAAGGT
Supt7l ex2-3	TTCCAGATCCGGCACAGTGA	GGATTGTGGCGACTGCTTGA
Slc4a1ap ex1-2	CAGCACTCGGCTCTTTATCC	TGCTGCTGTTTACGCAATTC
***Ccnl1* locus**		
Lekr1 (short) ex2-3	CCCATGCATGCCTTACCTGA	TGAAGAATTAGATAGCTGACGCCACA
Ccnl1 ex1-2	AGACGGATCTGCGCATCTTG	GAACCGATGGAACAGCACCTG
Tiparp ex1-2	CGTTCGAAGGGCGGCTTG	CAGTCTGGCTCAGGTTCAGTGG
***Cobll1* locus**		
Grb14 ex3-4	TTGCCAGCTGTTGATCCTGAA	TCAGTTGGCAGCTCGTGGTC
Cobll1 ex2-3	AGTGGTCCTGCCGGGAGATA	GAGCACAGAGGAACACGAGCA
Slc38a11 ex3-4	GCAAGCTGGATTTCCCTTGG	TCCAGAGAGGGCTCCTCCTTTT
***Slc32a1* locus**		
Adig ex1-2	CGTGGCTTTGCTGCTGTTCTT	CTCCAGGGCTCCCAGTTGAA
Slc32a1 ex1-2	AGTTCGGGGGTCACGACAAA	TAGGGTAGGCCCAGCACGAA
Actr5 ex2-3	GCGGCCTCATCATTTCATCC	GCCAGCTGCCTGACTTCCTC
Ppp1r16b ex1-2	ACCACCAGCTGCGGCTCT	GAGCACAGGGGCTCCCAAG
**Additional primers**		
Pparg2 ex2-4	TTTGAAAGAAGCGGTGAACCA	CGAAGTTGGTGGGCCAGAAT

Supplementary Table 26. Imputation quality distribution for the leptin-associated loci.

				Imputation quality				
**SNP**	**Chr**	**Pos**	**Nearby gene**	**95%**	**75%**	**50%**	**25%**	**5%**
rs1048750[illegible]	7	127647399	*LEP*	1.00	1.00	0.95	0.93	0.91
rs780093	2	27596107	*GCKR*	1.00	1.00	1.00	1.00	0.94
rs900400	3	158281469	*CCNL1*	0.99	0.99	0.96	0.93	0.55
rs6071166	20	36766426	*SLC32A1*	0.98	0.95	0.94	0.92	0.88
rs6738627	2	165252696	*COBLL1*	1.00	0.99	0.98	0.96	0.91
rs8043757	16	52370951	*FTO*	1.00	1.00	1.00	0.99	0.94

Supplementary Note 1

Acknowledgements

BLSA: The BLSA was supported by the Intramural Research Program of the NIH, National Institute on Aging.

BWHHS: The British Women Heart and Health Study is supported by British Heart Foundation programme grant no. PG/13/66/30442 and Department of Health Policy Research Programme (0090049). Genotype analysis in BWHHS was supported by the BHF (PG/07/131/24254) and UK Medical Research Council MC_UU_12013/8.

COLAUS: The CoLaus study received financial contributions from GlaxoSmithKline, the Faculty of Biology and Medicine of Lausanne, and the Swiss National Science Foundation (33CSCO-122661, 33CS30-139468 and 33CS30-148401). The authors thank Vincent Mooser and Dawn Waterworth, Co-PIs of the CoLaus study. Special thanks to Murielle Bochud, Yolande Barreau, Mathieu Firmann, Vladimir Mayor, Anne-Lise Bastian, Binasa Ramic, Martine Moranville, Martine Baumer, Marcy Sagette, Jeanne Ecoffey and Sylvie Mermoud for data collection. S.B. is grateful for financial support from the Swiss National Science Foundation (Grant #3100AO-116323/1) and the Swiss Institute of Bioinformatics.

Ely: We are grateful to all the volunteers and to the staff of St. Mary's Street Surgery, Ely and the study team. The Ely Study was funded by the MRC (MC_U106179471) and Diabetes UK. Genotyping in the Ely and Fenland studies was supported in part by an MRC-GlaxoSmithKline pilot programme grant (G0701863).

ERF: The genotyping for the ERF study was supported by EUROSPAN (European Special Populations Research Network) and the European Commission FP6 STRP grant (018947; LSHG-CT-2006-01947). The ERF study was further supported by grants from the Netherlands Organisation for Scientific Research, Erasmus MC, the Centre for Medical Systems Biology (CMSB) and the Netherlands Brain Foundation (HersenStichting Nederland). We are grateful to all participating individuals and their relatives, general practitioners and neurologists for their contributions and to P. Veraart for her help in genealogy, Jeannette Vergeer for the supervision of the laboratory work and P. Snijders for his help in data collection.

Family Heart Study: The Family Heart Study is funded by NHLBI grants 5R01HL068891, 5R01HL087700 and R01HL117078, and NIDDK grants 5R01DK068336, 5R01DK075681 and R01DK089256.

Fenland: The Fenland Study is funded by the Wellcome Trust and the Medical Research Council (MC_U106179471). We are grateful to all the volunteers for their time and help, and to the General Practitioners and practice staff for assistance with recruitment. We thank the Fenland Study Investigators, Fenland Study Co-ordination team and the Epidemiology Field, Data and Laboratory teams.

FINRISK97: This study has been funded by the Academy of Finland (grant numbers 139635, 129494, 118065, 129322, 250207, 136895, 263836), the Orion-Farmos Research Foundation, the Finnish Foundation for Cardiovascular Research, and the Sigrid Jusélius Foundation. We are grateful for the THL DNA laboratory for its skillful work to produce the DNA samples used in this study. We thank the Sanger Institute genotyping facilities for genotyping the samples.

GARP: We thank all participants of the GARP study. The Leiden University Medical Centre, the Dutch Arthritis Association and Pfizer Inc., Groton, CT, USA support the GARP study, whilst genotypic work was supported by the Netherlands Organization of Scientific Research (MW 904-61-095, 911-03-016, 917 66344 and 911-03-012), Leiden University Medical Centre, and by the “Centre of Medical Systems Biology” and the “Netherlands Consortium of Healthy Aging” in the framework of the Netherlands Genomics Initiative (NGI). Furthermore, the research leading to these results has received funding from the Dutch Arthritis Association (DAA 2010_017) and the European Union's Seventh Framework Programme (FP7/2007-2011) under grant agreement n° 259679.

GEMS: We acknowledge GEMS Study Investigators: Philip Barter, PhD; Antero Kesäniemi, PhD; Robert W. Mahley, PhD; Ruth McPherson, FRCP; Gerard Waeber MD; and Scott M. Grundy, PhD.

GLACIER: We thank the participants, health professionals and data managers involved in the Northern Swedish Health and Disease Study. We specifically thank John Hutiainen and Åsa Ågren (Umeå Medical Biobank) for data organization, and Kerstin Enquist and Thore Johansson (Västerbottens County Council) for expert technical assistance with DNA preparation. The GLACIER Study was funded by project grants from Novo Nordisk, the Swedish Heart-Lung Foundation, the Swedish Diabetes Association, Påhlssons Foundation, the Swedish Research Council, Umeå University Career Development Award, and The Heart Foundation of Northern Sweden (all to P.W.F). F.R. was supported by a post-doctoral stipend from the Swedish Heart-Lung Foundation.

GOOD: Financial support was received from the Swedish Research Council, the Swedish Foundation for Strategic Research, the ALF/LUA research grant in Gothenburg, the Lundberg Foundation, the Torsten and Ragnar Söderberg’s Foundation, the Novo Nordisk Foundation, and the European Commission grant HEALTH-F2-2008-201865-GEFOS.

GOYA: This study was conducted as part of the activities of the Gene-Diet Interactions in Obesity project (GENDINOB, www.gendinob.dk) and the MRC centre for Causal Analyses in Translational Epidemiology (MRC CAiTE). The data used in the present study is the ADIGEN subset of the GOYA-male cohort that underwent a thorough clinical-physiological examination, supported by the Danish Medical Research Council. We thank all the participants of the study. Tarunveer S. Ahluwalia received his Post Doctoral Fellowship from the GENDINOB project and acknowledges the same.

Health2000: This study has been funded by the Academy of Finland (grant numbers 129322 and 250207), the Orion-Farmos Research Foundation, the Finnish Foundation for Cardiovascular Research, and the Sigrid Jusélius Foundation. The Health 2000 Study was funded by the National Institute for Health and Welfare

(THL), the Finnish Centre for Pensions (ETK), the Social Insurance Institution of Finland (KELA), the Local Government Pensions Institution (KEVA) and other organizations listed on the website of the survey (http://www.terveys2000.fi). We are grateful for the THL DNA laboratory for its skillful work to produce the DNA samples used in this study. We thank the Sanger Institute genotyping facilities for genotyping the GenMets subcohort.

HealthABC: The Health ABC Study was supported by NIA contracts N01AG62101, N01AG62103, and N01AG62106 and, in part, by the NIA Intramural Research Program. The genome-wide association study was funded by NIA grant 1R01AG032098-01A1 to Wake Forest University Health Sciences and genotyping services were provided by the Center for Inherited Disease Research (CIDR). CIDR is fully funded through a federal contract from the National Institutes of Health to The Johns Hopkins University, contract number HHSN268200782096C. This study utilized the high-performance computational capabilities of the Biowulf Linux cluster at the National Institutes of Health, Bethesda, MD (http://biowulf.nih.gov).

HBCS: We thank all study participants as well as everybody involved in the Helsinki Birth Cohort Study. Helsinki Birth Cohort Study has been supported by grants from the Academy of Finland (130326, 134791, 263924), the Finnish Diabetes Research Society, Folkhälsan Research Foundation, Novo Nordisk Foundation, Finska Läkaresällskapet, Signe and Ane Gyllenberg Foundation, Ahokas Foundation, Emil Aaltonen Foundation, Juho Vainio Foundation, Finnish Foundation for Pediatric Research, Sigrid Juselius Foundation, the Finnish Special Governmental Subsidy for Health Sciences, Samfundet Folkhälsan, Liv och Hälsa, and Wellcome Trust (grant number WT089062). We are grateful for the THL DNA laboratory for its skillful work to produce the DNA samples used in this study. We thank the Sanger Institute genotyping facilities for genotyping the samples.

HERITAGE: We thank all PIs who contributed to the HERITAGE Family Study in the past via support from the National Heart, Lung, and Blood Institute (NHLBI) through the following grants: C Bouchard (HL-45670); AS Leon (HL-47323); DC Rao (HL-47317); JS Skinner (HL-47327); JH Wilmore (HL-47321).

InCHIANTI: The InCHIANTI study baseline (1998-2000) was supported as a "targeted project" (ICS110.1/RF97.71) by the Italian Ministry of Health and in part by the U.S. National Institute on Aging (Contracts: 263 MD 9164 and 263 MD 821336).

INTER99: The Novo Nordisk Foundation Center for Basic Metabolic Research is an independent research center at the University of Copenhagen partially funded by an unrestricted donation from the Novo Nordisk Foundation (www.metabol.ku.dk). The Inter99 study was initiated by Torben Jørgensen (PI), Knut Borch-Johnsen (co-PI), Hans Ibsen and Troels F. Thomsen. The steering committee comprises Torben Jørgensen (PI) and Charlotta Pisinger. The study was financially supported by research grants from the Danish Research Council, the Danish Centre for Health Technology Assessment, Novo Nordisk Inc., Research Foundation of Copenhagen County, Ministry of Internal Affairs and Health, the Danish Heart Foundation, the Danish Pharmaceutical Association, the Augustinus Foundation, the Ib Henriksen Foundation, the Becket Foundation, and the Danish Diabetes Association.

KORA: We thank all members of the field staff involved in the KORA study and we express our appreciation to all study participants. We thank the KORA Study Group consisting of A. Peters (speaker), J. Heinrich, R. Holle, R. Leidl, C. Meisinger, K. Strauch, and their co-workers, who are responsible for the design and conduct of the KORA studies. The KORA research platform (KORA, Cooperative Research in the Region of Augsburg) was initiated and financed by the Helmholtz Zentrum München - German Research Center for Environmental Health, which is funded by the German Federal Ministry of Education and Research and by the State of Bavaria. Furthermore, KORA research was supported within the Munich Center of Health Sciences (MC Health), Ludwig-Maximilians-Universität, as part of LMUinnovativ. This study was supported in part by a grant from the German Federal Ministry of Education and Research (BMBF) to the German Center for Diabetes Research (DZD e.V.).

LLS: The research leading to these results has received funding from the European Union's Seventh Framework Programme (FP7/2007-2011) under grant agreement number 259679. This study was financially supported by the Innovation-Oriented Research Program on Genomics (SenterNovem IGE05007), the Centre for Medical Systems Biology and the Netherlands Consortium for Healthy Ageing (grant 050-060-810), all in the framework of the Netherlands Genomics Initiative, Netherlands Organization for Scientific Research (NWO), by Unilever Colworth and by BBMRI-NL, a Research Infrastructure financed by the Dutch government (NWO 184.021.007).

LURIC: We extend our appreciation to the participants of the LURIC study; without their collaboration, this article would not have been written. We thank the LURIC study team who were either temporarily or permanently involved in patient recruitment as well as sample and data handling, in addition to the laboratory staff at the Ludwigshafen General Hospital and the Universities of Freiburg and Ulm, Germany. This work was supported by the 7th Framework Program (integrated project AtheroRemo, grant agreement number 201668 and RiskyCAD, grant agreement number 305739) of the European Union and, by the INTERREG IV Oberrhein Program (Project A28, Genetic mechanisms of cardiovascular diseases) with support from the European Regional Development Fund (ERDF) and the Wissenschaftsoffensive TMO.

MESA: MESA and the MESA SHARe project are conducted and supported by the National Heart, Lung, and Blood Institute (NHLBI) in collaboration with MESA investigators. Support for MESA is provided by contracts N01-HC-95159, N01-HC-95160, N01-HC-95161, N01-HC-95162, N01-HC-95163, N01-HC-95164, N01-HC-95165, N01-HC-95166, N01-HC-95167, N01-HC-95168, N01-HC-95169, UL1-TR-001079, and UL1-TR-000040. Funding for SHARe genotyping was provided by NHLBI Contract N02-HL-64278. Genotyping was performed at Affymetrix (Santa Clara, California, USA) and the Broad Institute of Harvard and Massachusetts Institute of Technology (Boston, Massachusetts, USA) using the Affymetrix Genome-Wide Human SNP Array 6.0. Additional support was provided by R01-HL-088451. Leptin assays were funded by 1R01DK080015.

MICROS: For the MICROS study, we thank the primary care practitioners Raffaela Stocker, Stefan Waldner, Toni Pizzecco, Josef Plangger, Ugo Marcadent, and the personnel of the Hospital of Silandro (Department of Laboratory Medicine) for their participation and collaboration in the research project. The MICROS study was supported by the Ministry of Health and Department of Educational Assistance, University and

Research of the Autonomous Province of Bolzano, the South Tyrolean Sparkasse Foundation, and the European Union framework program 6 EUROSPAN project (contract no. LSHG-CT-2006-018947).

MrOS Sweden: Financial support was received from the Swedish Research Council, the Swedish Foundation for Strategic Research, the ALF/LUA research grant in Gothenburg, the Lundberg Foundation, the Torsten and Ragnar Söderberg's Foundation, the Novo Nordisk Foundation, and the European Commission grant HEALTH-F2-2008-201865-GEFOS.

MuTHER: The MuTHER Study was funded by a program grant from the Wellcome Trust (081917/Z/07/Z) and by core funding from the Wellcome Trust Centre for Human Genetics (090532). Additional funding came from Wellcome Trust and European Community's Seventh Framework Programme (FP7/2007-2013). The study also received support from the National Institute for Health Research (NIHR) Clinical Research Facility at Guy's & St. Thomas', National Health Service (NHS) Foundation Trust and NIHR Biomedical Research Centre at Guy's and St. Thomas' NHS Foundation Trust and King's College London. Genotyping was performed by the Wellcome Trust Sanger Institute and the National Eye Institute via a National Institutes of Health (NIH) project grant.

NHS: This research was supported by NIH grants HG004399, HG004446, CA087969, CA055075, DK058845, CA65725, CA49449, CA67262, CA50385, and UO1CA098233. Qi Sun was supported by a career development award (R00HL098459) from the NHLBI.

PIVUS: This research was supported by funding from the Swedish Heart-Lung Foundation (20140422), Knut and Alice Wallenberg Foundation, Swedish Diabetes Foundation (grant no. 2013-024), Swedish Research Council (grant no. 2012-1397), and Göran Gustafsson Foundation. APM is a Wellcome Trust Senior Research Fellow and acknowledges funding from the Wellcome Trust under awards WT098017, WT090532 and WT064890. C.M.L. is a Wellcome Trust Research Career Development Fellow (086596/Z/08/Z) and acknowledges funding from the Li Ka Shing Foundation.

PROSPER: The PROSPER study was supported by an investigator initiated grant obtained from Bristol-Myers Squibb. Prof. Dr. J. W. Jukema is an Established Clinical Investigator of the Netherlands Heart Foundation (grant 2001 D 032). Support for genotyping was provided by the seventh framework program of the European commission (grant 223004) and by the Netherlands Genomics Initiative (Netherlands Consortium for Healthy Aging grant 050-060-810).

QFS: The Quebec Family Study was funded by multiple grants from the Medical Research Council of Canada and the Canadian Institutes for Health Research. This work was supported by a team grant from the Canadian Institutes of Health Research (FRCN-CCT-83028).

RISC: The RISC Study was supported by European Union grant QLG1-CT-2001-01252 and AstraZeneca. David B. Savage acknowledges funding from the Wellcome Trust (grant no 091551). Tim Frayling is supported by the European Research Council grant 323195:GLUCOSEGENES-FP7-IDEAS-ERC.

Rotterdam Study: The generation and management of GWAS genotype data for the Rotterdam Study (RS I, RS II, RS III) was executed by the Human Genotyping Facility of the Genetic Laboratory of the Department of Internal Medicine, Erasmus MC, Rotterdam, The Netherlands. The GWAS datasets are supported by the Netherlands Organisation of Scientific Research NWO Investments (nr. 175.010.2005.011, 911-03-012), the Genetic Laboratory of the Department of Internal Medicine, Erasmus MC, the Research Institute for Diseases in the Elderly (014-93-015; RIDE2), the Netherlands Genomics Initiative (NGI)/Netherlands Organisation for Scientific Research (NWO) Netherlands Consortium for Healthy Aging (NCHA), project nr. 050-060-810. We thank Pascal Arp, Mila Jhamai, Marijn Verkerk, Lizbeth Herrera and Marjolein Peters, MSc, and Carolina Medina-Gomez, MSc, for their help in creating the GWAS database, and Karol Estrada, PhD, Yurii Aulchenko, PhD, and Carolina Medina-Gomez, MSc, for the creation and analysis of imputed data. The Rotterdam Study is funded by Erasmus Medical Center and Erasmus University, Rotterdam, the Netherlands Organization for the Health Research and Development (ZonMw), the Research Institute for Diseases in the Elderly (RIDE), the Ministry of Education, Culture and Science, the Ministry for Health, Welfare and Sports, the European Commission (DG XII), and the Municipality of Rotterdam. The authors are grateful to the study participants, the staff from the Rotterdam Study and the participating general practitioners and pharmacists.

TwinsUK: The study was funded by the Wellcome Trust; European Community's Seventh Framework Programme (FP7/2007-2013). The study also receives support from the National Institute for Health Research (NIHR) BioResource Clinical Research Facility and Biomedical Research Centre based at Guy's and St Thomas', NHS Foundation Trust and King's College London. Brent Richards is funded by the CIHR, MDEIE, FRSQ and CFI. SNP Genotyping was performed by The Wellcome Trust Sanger Institute and National Eye Institute via NIH/CIDR.

WGHS: The WGHS is supported by HL043851 and HL080467 from the National Heart, Lung, and Blood Institute and CA047988 from the National Cancer Institute and the Donald W. Reynolds Foundation, with collaborative scientific support and funding for genotyping provided by Amgen. Leptin measures were funded by an investigator-initiated grant from Roche Pharmaceuticals to PMR.

Young Finns: The Young Finns Study has been financially supported by the Academy of Finland: grants 286284 (T.L.), 134309 (Eye), 126925, 121584, 124282, 129378 (Salve), 117787 (Gendi), and 41071 (Skidi); the Social Insurance Institution of Finland; Kuopio, Tampere and Turku University Hospital Medical Funds (grant X51001 for T.L.); Juho Vainio Foundation; Paavo Nurmi Foundation; Finnish Foundation of Cardiovascular Research (T.L.); Finnish Cultural Foundation; Tampere Tuberculosis Foundation (T.L.); Emil Aaltonen Foundation (T.L.); and Yrjö Jahnsson Foundation (T.L.). We gratefully acknowledge the THL DNA laboratory for its skillful work to produce the DNA samples used in this study, and Ville Aalto and Irina Lisinen for the expert technical assistance in the statistical analyses.

Other acknowledgements: This work was supported in part through the computational resources and staff expertise provided by the Department of Scientific Computing at the Icahn School of Medicine at Mount Sinai. Tuomas O. Kilpeläinen was supported by the Danish Council for Independent Research (DFF – 1333-00124 and Sapere Aude program grant DFF – 1331-00730B). Robert J. Klein was supported by the NHGRI

grant U01-HG007033. Tune H Pers is funded by The Lundbeck Foundation. Data on the childhood obesity trait has been contributed by EGG Consortium and has been downloaded from www.egg-consortium.org. Data on glycaemic traits have been contributed by MAGIC investigators and have been downloaded from www.magicinvestigators.org. This research was conducted prior to Ms. Garcia's current affiliation with the National Heart, Lung, and Blood Institute. As such, the views expressed in this article do not necessarily represent the views of the National Heart, Lung, and Blood Institute, National Institutes of Health, or any other government entity.

Supplementary References

1. Shock, N.W. *et al.* Normal Human Aging: The Baltimore Study of Aging. *NIH Publication* No. 84-2540 (1984).
2. Firmann, M. *et al.* The CoLaus study: a population-based study to investigate the epidemiology and genetic determinants of cardiovascular risk factors and metabolic syndrome. *BMC Cardiovasc Disord* **8**, 6 (2008).
3. Aulchenko, Y.S. *et al.* Linkage disequilibrium in young genetically isolated Dutch population. E*ur J Hum Genet* **12**, 527-34 (2004).
4. Broer, L. *et al.* Association of adiponectin and leptin with relative telomere length in seven independent cohorts including 14,448 participants. *Eur J Epidemiol* **29**, 629-38 (2014).
5. Higgins, M. *et al.* NHLBI Family Heart Study: objectives and design. *Am J Epidemiol* **143**, 1219-28 (1996).
6. Ma, D. *et al.* Leptin is associated with blood pressure and hypertension in women from the National Heart, Lung, and Blood Institute Family Heart Study. *Hypertension* **53**, 473-9 (2009).
7. Rolfe Ede, L. *et al.* Association between birth weight and visceral fat in adults. *Am J Clin Nutr* **92**:347-52 (2010).
8. Willer, C.J. *et al.* Six new loci associated with body mass index highlight a neuronal influence on body weight regulation. *Nat Genet* **41**, 25-34 (2009).
9. Blankenberg, S. *et al.* Contribution of 30 biomarkers to 10-year cardiovascular risk estimation in 2 population cohorts: the MONICA, risk, genetics, archiving, and monograph (MORGAM) biomarker project. *Circulation* **121**, 2388-97 (2010).
10. Stimadel, H. *et al.* Genetic and phenotypic architecture of metabolic syndrome-associated components in dyslipidemic and normolipidemic subjects: the GEMS Study. *Atherosclerosis* **197**, 868-76 (2008).
11. Lorentzon, M. *et al.* Free testosterone is a positive, whereas free estradiol is a negative, predictor of cortical bone size in young Swedish men: the GOOD study. *J Bone Miner Res* **20**, 1334-41 (2005).
12. Aromaa, A., Koskinen, S. Health and functional capacity in Finland. Baseline results of the Health 2000 Health Examination Survey. *Publications of the National Public Health Institute* **B12/2004**
13. Eriksson, J.G. Early growth and adult health outcomes—lessons learned from the Helsinki Birth Cohort Study. *Matern Child Nutr* **1**, 149-54 (2005).
14. Bouchard, C. *et al.* The HERITAGE Family Study. Aims, design and measurement protocol. *Med Sci Sports Exerc* **27**, 721-9 (1995).

15. Ferrucci, L. *et al.* Subsystems contributing to the decline in ability to walk: bridging the gap between epidemiology and geriatric practice in the InCHIANTI study. *J Am Geriatr Soc* **48**, 1618-25 (2000).
16. Holle, R. *et al.* KORA—a research platform for population based health research. *Gesundheitswesen* **67 Suppl 1**, S19-25 (2005).
17. Pattaro, C. *et al.* The genetic study of three population microisolates in South Tyrol (MICROS): study design and epidemiological perspectives. *BMC Med Genet* **8**, 29 (2007).
18. Sun, Q. *et al.* Leptin and soluble leptin receptor levels in plasma and risk of type 2 diabetes in U.S. Women: A Prospective Study. *Diabetes* **59**, 611-8 (2010).
19. Sun, Q. *et al.* Genome-wide association study identifies polymorphisms in LEPR as determinants of plasma soluble leptin receptor levels. *Hum Mol Genet* **20**, 1846-55 (2010).
20. Hunter, D.J. *et al.* A genome-wide association study identifies alleles in FGFR2 associated with risk of sporadic postmenopausal breast cancer. *Nat Genet* **39**, 870-4 (2007).
21. Rice, T. *et al.* Familial resemblance for plasma leptin: sample homogeneity across adiposity and ethnic groups. *Obes Res* **10**, 351-60 (2002).
22. Hill, S.A. *et al.* The EGIR-RISK STUDY (The European group for the study of insulin resistance: relationship between insulin sensitivity and cardiovascular disease risk): I. Methodology and objectives. *Diabetologia* **47**, 566-70 (2004).
23. Finucane, F.M. *et al.* Correlation of the leptin:adiponectin ratio with measures of insulin resistance in non-diabetic individuals. *Diabetologia* **52**, 2345-9 (2009).
24. Estrada, K. *et al.* GRIMP: a web- and grid-based tool for high-speed analysis of large-scale genome-wide association using imputed data. *Bioinformatics* **25**, 2750-2 (2009).
25. Hofman, A. *et al.* The Rotterdam Study: 2012 objectives and design update. *Eur J Epidemiol* **26**, 657-86 (2011).
26. Hofman, A. *et al.* Determinants of disease and disability in the elderly: the Rotterdam Elderly Study. *Eur J Epidemiol* **7**, 403-22 (1991).
27. Moayyeri, A *et al.* The UK Adult Twin Registry. *Twin Res Hum Genet* **16**, 144-9 (2013).
28. Raitakari, O.T. *et al.* Cohort profile: the cardiovascular risk in Young Finns Study. *Int J Epidemiol* **37**, 1220-6 (2008).
29. Lawlor, D.A. *et al.* Leptin and coronary heart disease risk: prospective case control study of British women. *Obesity* **15**:1694-701 (2007).
30. Forouhi, N.G. *et al.* Incidence of type 2 diabetes in England and its association with baseline impaired fasting glucose: the Ely study 1990-2000. *Diabet Med* **24**, 200-7 (2007).
31. Riyazi, N. *et al.* Evidence for familial aggregation of hand, hip, and spine but not knee osteoarthritis in siblings with multiple joint involvement: the GARP study. *Ann Rheum Dis* **64**, 438-43 (2005).
32. Yusuf, E. *et al.* Association between leptin, adiponectin and resistin and long-term progression of hand osteoarthritis. *Ann Rheum Dis* **70**, 1282-4 (2011).
33. Renström, F. *et al.* Genetic predisposition to long-term nondiabetic deteriorations in glucose homeostasis: Ten-year follow-up of the GLACIER study. *Diabetes* **60**, 345-54 (2011).
34. Kurbasic, A. *et al.* Gene-lifestyle interactions in complex diseases: Design and description of the GLACIER and VIKING studies. *Curr Nutr Rep* **3**, 400-411 (2014).
35. Paternoster, L. *et al.* Genome-wide population-based association study of extremely overweight young adults—the GOYA study. *PLoS One* **6**, e24303 (2011).

36. Winkelmann, B.R. *et al.* Rationale and design of the LURIC study—a ressource for functional genomics, pharmacogenomics and long-term prognosis of cardiovascular disease. *Pharmacogenomics* **2(1 Suppl 1)**, S1-73 (2001).
37. Bild, D.E. *et al.* Multi-ethnic study of atherosclerosis: objectives and design. *Am J Epidemiol* **156**, 871-81 (2002).
38. Rasmussen-Torvik, L.J. *et al.* Associations of body mass index and insulin resistance with leptin, adiponectin, and the leptin-to-adiponectin ratio across ethnic groups: the Multi-Ethnic Study of Atherosclerosis (MESA). *Ann Epidemiol* **22**, 705-9 (2012).
39. Mellström D, *et al.* Free testosterone is an independent predictor of BMD and prevalent fractures in elderly men: MrOS Sweden. *J Bone Miner Res* **21**, 529-35 (2006).
40. Lind, L. *et al.* A comparison of three different methods to determine arterial compliance in the elderly: the Prospective Investigation of the Vasculature in Uppsala Seniors (PIVUS) study. *J Hypertens* **24**, 1075-82 (2006).
41. Welsh, P. *et al.* Unraveling the directional link between adiposity and inflammation: a bidirectional Mendelian randomization approach. *J Clin Endocrinol Metab* **95**, 93-9 (2010).
42. Locke, A.E. *et al.* Genetic studies of body mass index yield new insights for obesity biology. *Nature* **518**, 197-206 (2015)
43. Shungin, D. *et al.* New genetic loci link adipose and insulin biology to body fat distribution. *Nature* **518**, 187-96 (2015)
44. Wood, A.R. *et al.* Defining the role of common variation in the genomic and biological architecture of adult human height. *Nat Genet* **46**, 1173-86 (2014)
45. Lu Y., *et al.* New loci for body fat percentage reveal link between adiposity and cardiometabolic disease risk. *Nat Commun* [in press] (2015)
46. Manning, A.K. *et al.* A genome-wide approach accounting for body mass index identifies genetic variants influencing fasting glycemic traits and insulin resistance. *Nat Genet* **44**, 659-69 (2012)
47. Willer, C. J. *et al.* Discovery and refinement of loci associated with lipid levels. *Nat Genet* **45**, 1274-83 (2013)
48. Ehret, G.B. *et al.* Genetic variants in novel pathways influence blood pressure and cardiovascular disease risk. *Nature* **478**, 103-9 (2011)
49. Horikoshi, M. *et al.* New loci associated with birth weight identify genetic links between intrauterine growth and adult height and metabolism. *Nat Genet* **45**, 76-82 (2013)
50. Dastani, Z. *et al.* Novel loci for adiponectin levels and their influence on type 2 diabetes and metabolic traits: a multi-ethnic meta-analysis of 45,891 individuals. *PLoS Genet* **8**, e1002607 (2012)
51. Perry, J.R. *et al.* Parent-of-origin-specific allelic associations among 106 genomic loci for age at menarche. *Nature* **514**, 92-7 (2014)
52. Dehghan, A. *et al.* Meta-analysis of genome-wide association studies in >80,000 subjects identifies multiple loci for C-reactive protein levels. *Circulation* **123**, 731-8 (2011)
53. Tanaka, T. *et al.* Genome-wide meta-analysis of observational studies shows common genetic variants associated with macronutrient intake. *Am J Hum Genet* **97**, 1395-402 (2013).
54. Wheeler, E. *et al.* Genome-wide SNP and CNV analysis identifies common and low-frequency variants associated with severe early-onset obesity. *Nat Genet* **45**, 513-7 (2013).
55. Morris, A.P. *et al.* Large-scale association analysis provides insights into the genetic architecture and pathophysiology of type 2 diabetes. *Nat Genet* **44**, 981-90 (2012).

56. Zhong, H. *et al.* Integrating pathway analysis and genetics of gene expression for genome-wide association studies. Am J Hum Genet **86**, 581-91 (2010).
57. Myers, A.J. *et al.* A survey of genetic human cortical gene expression. *Nat Genet* **39**, 1494-9 (2007).
58. Min, J.L. *et al.* Coexpression network analysis in abdominal and gluteal adipose tissue reveals regulatory genetic loci for metabolic syndrome and related phenotypes. *PLoS Genet* **8**, e1002505 (2012).
59. Grundberg,, E. *et al.* Mapping cis- and trans-regulatory effects across multiple tissues in twins. *Nat Genet* **44**, 1084-9 (2012).
